# Major Components of *Dittrichia viscosa* (Asteraceae) as a Source of New Pesticides

**DOI:** 10.3390/molecules30193950

**Published:** 2025-10-01

**Authors:** María José Segura-Navarro, José Francisco Quílez del Moral, María Fe Andrés, Félix Valcárcel, Azucena González-Coloma, Diego O. Molina Inzunza, Alejandro F. Barrero

**Affiliations:** 1 Departamento de Química Orgánica, Instituto de Biotecnología, Universidad de Granada, 18071 Granada, Spain; mariajseguranavarro@gmail.com (M.J.S.-N.); diegomolina96@gmail.com (D.O.M.I.); 2 Instituto de Ciencias Agrarias, Centro Superior de Investigaciones Científicas, 28006 Madrid, Spain; mafay@ica.csic.es; 3 Instituto Nacional de Investigación y Tecnología Agraria y Alimentaria, Centro Superior de Investigaciones Científicas, 28040 Madrid, Spain; valcarcel.felix@inia.csic.es

**Keywords:** natural products, terpenes, organic synthesis, bioactive compounds, insecticidal, nematicidal

## Abstract

Ilicic acid, nerolidol, and 9-hydroxynerolidol are major components of the aerial parts of *Dittrichia viscosa*. These components were selectively isolated in multigram quantities and used as lead compounds to generate diversity in the search for new natural-product-derived pesticides. A total of 29 derivatives of these three molecules—some of which are known natural products—were generated by subjecting these natural products to different transformations. In order to explore potential applications in sustainable biocontrol, some of the compounds generated were evaluated for plant protection potential against insect pests (*Spodoptera littoralis*, *Myzus persicae*, *Rhopalosiphum padi*), against the nematode *Meloidogyne javanica*, and for their phytotoxic effects on ryegrass (*Lolium perenne*) and lettuce (*Lactuca sativa*). Additionally, their effects against the tick *Hyalomma lusitanicum* have been tested. Compound **11** was found to be antifeedant against *S. littoralis* and nematicidal. Compounds **3a** and **8** were potent antifeedants against *R. padi*. None of the tested compounds significantly inhibited lettuce growth, and compounds **17**, **3**, and **3a** even promoted root development. Conversely, compounds **3**, **4**, **11**, **17**, and **21a** exhibited strong herbicidal activity on ryegrass. In larvicidal assays against *H. lusitanicum*, compounds **3**, **3a**, **11**, **17**, **29**, and **33** were active, with compound **29** being six times more active than the positive control nootkatone.

## 1. Introduction

*Dittrichia viscosa* (L., 1753) Greuter, 1973 is an herbaceous perennial plant belonging to the Asteraceae family and the Asterales order, widely distributed throughout the Mediterranean basin. It thrives in environments modified by anthropogenic activities (roadsides, abandoned fields, or suburban areas) thanks to its remarkable colonization capacity. This species is also highly resistant to adverse conditions and exhibits excellent adaptability [1,2].

The native range of *D. viscosa* includes the coasts of southern Europe (France, Spain, Greece, Italy, Bulgaria, Croatia, and Turkey), the Middle East (Israel, Jordan, and Syria), as well as northern Africa (Algeria, Egypt, and Libya) [1]. In this sense, its very efficient seed production and dispersal, together with its adaptation to different ecosystems, has enabled its establishment in the Azores, Belgium, and Great Britain. Furthermore, it is now common also in other regions of Europe and North America. It is declared and listed on the Alert List of Environmental Weeds in Australia and North America [2].

Regarding the ecological role played by this species, it is worth mentioning that *D. viscosa* exudates show marked cytotoxic and genotoxic activity, resulting in allelopathic effects on seed germination and root growth of other plants [3]. Worth mentioning, *D. viscosa* has been identified as a host for natural enemies that target agricultural pests such as the olive fruit fly (*Bactrocera oleae* Gmel) [4]. With respect to its tolerance to polluted environments, the plant shows a remarkable capacity to grow in soils enriched with nickel, magnesium, and arsenic. For this reason, it has been suggested as both a bioindicator of these elements and a potential candidate for phytoextraction strategies [5]. Lastly, although *D. viscosa* has been suspected of causing health issues in grazing livestock, some reports indicate that its aerial parts are consumed by sheep and goats without evident negative effects [6].

Chemical analyses of plant extracts revealed the presence of several metabolites belonging to different classes of natural products such as sesquiterpenes, flavonoids, and caffeic acids. Comprehensive reviews summarizing these studies, along with data on the quantification of individual constituents in specific plant organs, have been published [7].

Since the ancient Roman era, *D. viscosa* has been valued for its medicinal uses. Its applications include anti-inflammatory, analgesic, antibacterial, antifungal, antispasmodic, sedative, antiseptic, antipyretic, antidiarrheal, antirheumatic, antidiabetic, and expectorant activities, among others [8,9]. Significant activities of *D. viscosa* secondary metabolites were reported, including antitumoral effects against a wide range of cell lines: such as melanoma, breast, colorectal, cervical, skin, and brain cancers [9]; and insecticidal and nematicidal properties against *Spodoptera littoralis* larvae, *Myzus persicae*, *Rhopalosiphum padi*, and *Meloidogyne javanica* [10]. Moreover, *D. viscosa* supports the population growth of *Macrolophus melanotoma*, a predator of various agricultural pests, thereby contributing to natural pest control in crops [11].

Given the significant biological activity of some major compounds from this plant species, namely ilicic acid, nerolidol, and 9-hydroxinerolidol, we anticipated that these three compounds would be promising lead compounds in the search for new biopesticides. Indeed, ilicic acid showed allelopathic activity against the larval stage of *Tribolium castaneum* [12] and an antifeedant effect against *Leptinotarsa decemlineata* [13]. Similarly, nerolidol proved to possess toxic activity against the cotton leafworm *S. littoralis* [14] and the nematode *M. javanica* [15], which encouraged us to use this compound as well as 9-hydroxynerolidol to generate molecular diversity. To sum up, the three major natural products of *D. viscosa* have been used as lead compounds to produce molecules with potential biopesticide activity against key agricultural pests such as the insects *S. littoralis*, *M. persicae*, and *R. padi*, the root-knot nematode *M. javanica*, and the tick *Hyalomma lusitanicum.* Additionally, phytotoxic activity has been found against the monocot *Lolium perenne* and the dicot *Lactuca sativa*.

With the ultimate aim of generating the intended molecular diversity, it is worth underscoring the versatility of the iodine molecule to undergo several chemical transformations, including dehydration, aromatization, rearrangements, and others [16,17].

## 2. Results

### 2.1. Extraction of Major Compounds from the Aerial Parts of D. viscosa

We started this approach by studying different ways of extraction from the aerial parts of the plant. After some experimentation using different solvents and conditions, we selected the maceration of the plant material twice for 30 min with *tert*-butyl methyl ether (MTBE). This procedure led to an extract yield of about 5% on dry plant weight or 0.76% of fresh plant.

**Scheme 1:**
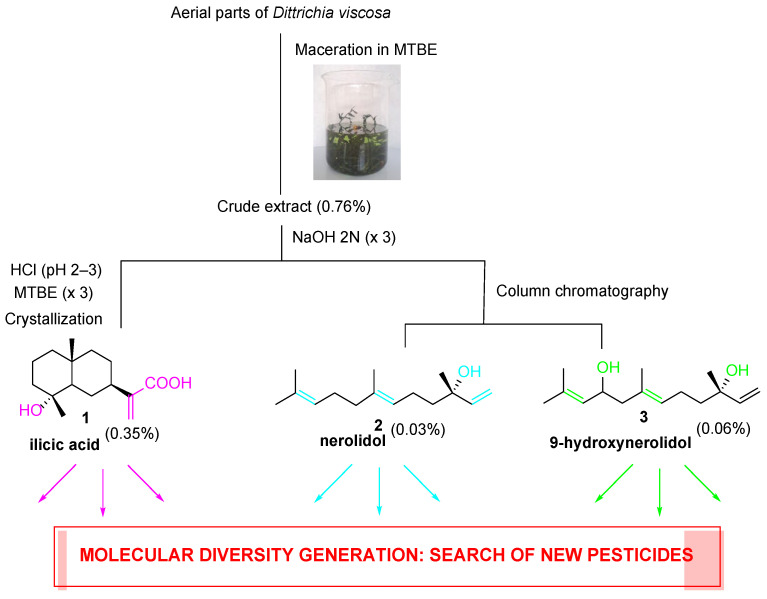
Obtention of ilicic acid, nerolidol, and 9-hydroxynerolidol from *D. viscosa* extract.

Fractionation of the extract obtained the from fresh plant with an aqueous solution of 2N NaOH led to the separation of an acid fraction (0.35%) containing, according to its ^1^H-NMR spectrum, ilicic acid together with flavones and other minor components, as well as a neutral fraction where nerolidol and 9-hydroxynerolidol were the main components (0.03% and 0.06%) of fresh plant, respectively. Gratifyingly, ilicic acid (**1**) was found to crystallize from the acid fraction using MTBE as solvent, while one flash chromatography was needed to obtain nerolidol (**2**) and 9-hydroxynerolidol (**3**) (Scheme 1).

### 2.2. Ilicic Acid as a Source of Molecular Diversity

As already mentioned, the previously reported pesticide activity of ilicic acid (**1**), along with the ability to obtain multigram quantities of this substance— following the protocol described above—makes this natural product a promising lead compound for exploring molecular diversity.

#### 2.2.1. Esterification of Ilicic Acid—Reactions with Iodine

To initiate this study, ester **4** was synthesized by treating ilicic acid with trimethylsilyldiazomethane. This compound has been previously identified in *Tithonia diversifolia* and some species of the *Artemisia* genus [18,19,20].

When ester **4** was reacted with iodine in benzene, the trisubstituted dehydration product **5** was obtained selectively. Higher reaction temperatures (using toluene as solvent) and longer reaction times continued to produce only the dehydration products **5**–**7** (Table 1, entry 2), all of which are known natural products [21]. The use of substechiometric quantities of iodine, with DMSO as regenerating agent (Table 1, entry 3) began to generate the desired molecular diversity, and compounds **8**–**10** were produced, although in very low yields. Notably, the acid derivative of ester **8** turned out to be a natural product with reported neuroprotective and pesticidal activities [22]. Most remarkably, compounds **9** and **10** present a fully conjugated structure, where up to six new unsaturations were formed, thus proving the high potentiality of this I_2_/DMSO system.

Structural assignment of both **9** and **10** was performed after analysis of their spectroscopical data. Thus, the molecular formula deduced for both substances (C_15_H_15_O_2_ and C_15_H_12_O_3_, from their HRMS ([M+H]^+^ at 227.1074, and [M+H]^+^ at 241.0861, respectively) confirmed the loss of one carbon atom. Their ^1^H NMR spectra, where six aromatic methines were observed in both cases, agreed with a structure of disubstituted naphthalene. One of these substituents was the conjugated methyl ester present in the starting material, while the second substituent was a methyl group in the case of **9** (^1^H: δ 2.71, 3H, s; ^13^C: δ 19.4) and an aldehyde group in the case of **10** (^1^H: δ 10.42, 1H, s; ^13^C: δ 193.5)

**Scheme 2:**
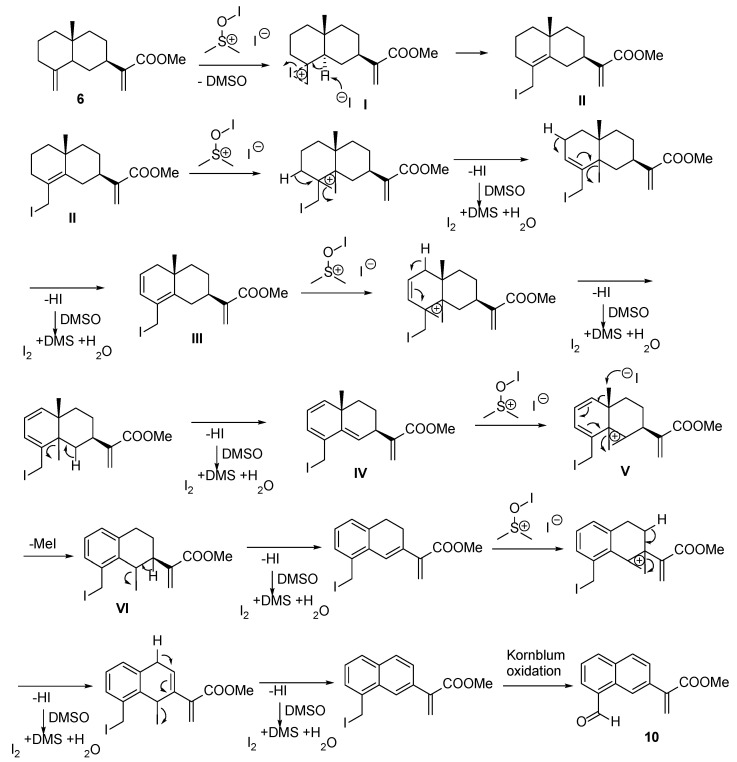
Proposed mechanism for the generation of aldehyde **10**.

A plausible mechanism that rationalizes the generation of the fully conjugated structure **10** is shown in Scheme 2. The process would start after the reaction of the methyl ester of β-costic acid **6** with I_2_/DMSO. This led, after formation of the exocyclic methylene iodonium **I** and elimination of H^+^, to the iododerivative **II**. From **II**, a reagent-mediated dehydrogenation should take place, leading to the conjugated diene **III**, which undergoes loss of HI to afford the triene **IV**. From this intermediate, a selective iodination occurs on the 5,6 double bond, giving rise to the iodonium **V**, which would evolve towards the aromatization of ring A via an S_N_2 process with iodide anion to generate intermediate **VI**. Final steps would include a loss of HI followed by further dehydrogenation, and a Kornblum oxidation, ultimately yielding compound **10** [16,17,23].

To improve the yields of the newly generated compounds, a solvent mixture of MeOH and toluene was tested as a solvent. In the event, after 6.5 h of reaction, 38% of transformed products were produced, while 45% of the dehydrated **5** remained unreacted. Together with **5**, the generation of compounds **12** and **13** was noticed, where the A-ring is aromatized. Additionally, the appearance of lactone **11** was also noticed. This compound, reported as a product of the reaction of ilicic acid with TsOH, presents moderate gastric cytoprotective activity [24]. Longer reaction times reduced the yield of the dehydration product **5** (Table 1, entries 5 and 6). Thus, when the reaction was prolonged up to 38 h, compound **5** was found in a 24% yield (Table 1, entry 6). Together with **5** and its free acid derivative **14**, it was noticed that the generation of compounds **15** and **17**, where now A and B, two rings are aromatized.

**Figure 1:**
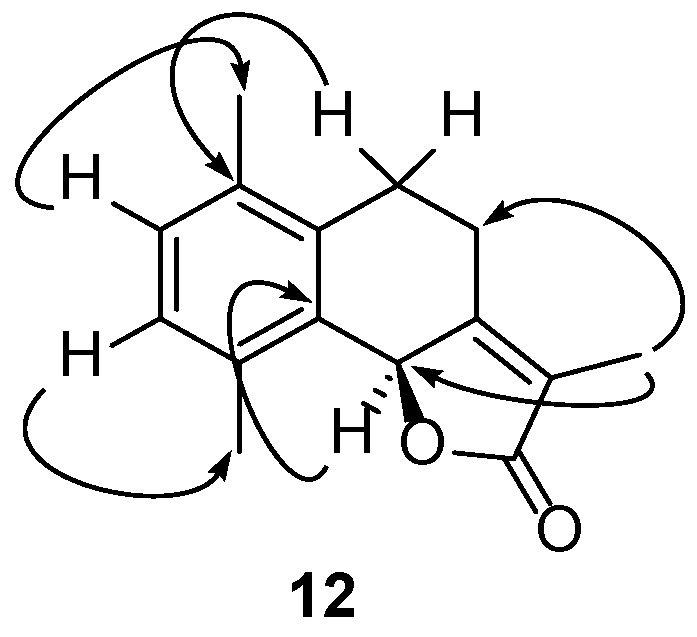
Key HMBC correlations for compound **12**.

The structural elucidation of compound **12** was realized after an exhaustive analysis of its spectroscopical data, including 2D NMR. Its ^1^H NMR spectrum showed signals corresponding to an aromatic AB system (A: 7.08 ppm, d, *J* = 7.8 Hz, 1H, B: 7.04 ppm, d, *J* = 7.8 Hz, 1H). No more aromatic protons were observed, which suggested that the aromatic ring is 1,2,3,4-tetrasubstituted. The presence of two singlet methyls at δ 2.61 and 2.21 indicated that these two methyl groups were located at positions 1 and 4 of the rearranged eudesmane A ring. The signal attributed to an oxygenated methine (^1^H NMR: δ 5.95, s, 1H), together with ^13^C NMR signals at δ 79.33 and 174.5, was compatible with the existence of a lactone moiety in the structure. Finally, the selected bidimensional correlations shown in Figure 1 confirmed the tricyclic core for compound **12**.

**Scheme 3:**
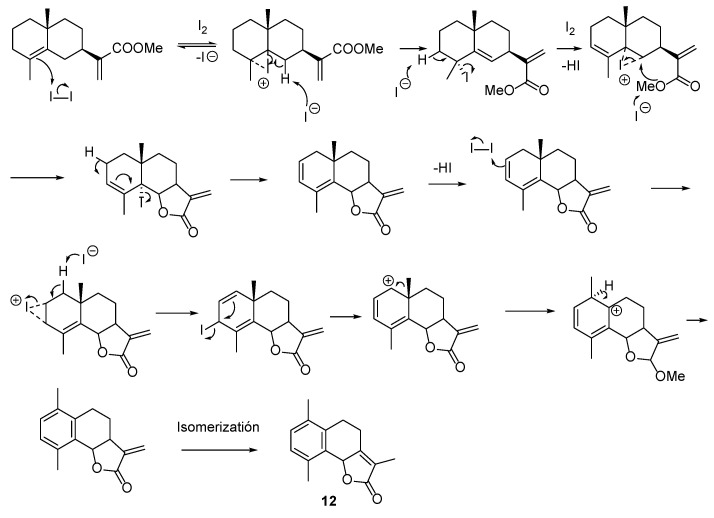
Proposed mechanism for the generation of compound **12**.

The proposed mechanism for the formation of **12** is shown in Scheme 3.

Compared with **12**, the NMR data of **13** showed signals compatible with the presence of a 2-methoxyfuran substructure (^1^H: δ 3.24, s, 3H; ^13^C: δ 50.1) as the most significant difference.

**Figure 2:**
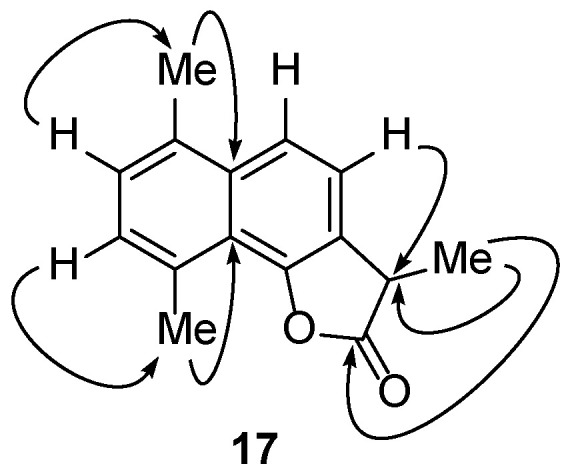
Key HMBC correlations for compound **17**.

The spectroscopical data of compound **15** also confirmed the aromatization of the B-ring in this rearranged eudesmane structure. Noticeably, the disubstituted double bond of the conjugated ester moiety, located at C7 in the starting material, appeared reduced in compound **15** [^1^H NMR; δ 3.96, (q, *J* = 7.2 Hz, 1H); 3.70 (s, 3H), 1.63 (d, *J* = 7.2 Hz, 3H)]. This double bond also appeared reduced in lactone **17**, whose structure was determined after the study of its 1D and 2D NMR spectra (Figure 2).

The structure of **13** was assigned based on the study of its one- and two-dimensional NMR spectra. Its ^1^H NMR spectrum shows the presence of two singlets at δ 6.11 and 5.55 ppm and a dd at 4.55. These signals, together with those found in its ^13^C NMR spectrum at δ 142.4 and 119.6 (corresponding to a methylene group), the signal at 76.90 ppm (assignable to an oxygenated methine), and the signal at 174.24 ppm, allow us to propose the presence in **13** of a γ-butyrolactone. The remaining NMR data of **16** confirmed that there is no additional function in this molecule and were compatible with an eudesmane skeleton, where the methyl at position 4 is reduced [0.97 (d, *J* = 6.0 Hz, 3H)]. The relative configuration of the chiral centers was proposed, on the one hand, considering that **16** maintained the stereochemistry at C1 and C7 of the starting ilicic acid. On the other hand, the value of the coupling constants of H6 (dd, *J* = 5.2, 3.6 Hz) is compatible with those described for other *cis*-eudesman-12,6-olides [25] with a *trans* interannular junction. Additionally, the value of the chemical shift of C-15 and C14, both below 20 ppm, led us to propose the β disposition of methyl at C4.

It is noteworthy that the toluene/MeOH solvent system promoted lactone formation, in contrast to the I_2_/DMSO protocol, which generated fully conjugated esters **9** and **10** (albeit in low yields and with loss of the C10 methyl group). Selective formation of A-ring or dual-ring aromatized compounds was dependent on reaction conditions. Interestingly, compound **15** shows structural resemblance to naproxen, a non-steroidal anti-inflammatory drug, prompting potential future studies into its anti-inflammatory properties.

#### 2.2.2. Reactions of γ-Costic Acid Methyl Ester (**5**) with Iodine

Noticing the intermediacy of compound **5** in the reaction of compound **4** with iodine and taking advantage of the fact that this compound can be selectively obtained from the methyl ester of ilicic acid, we decided to use this compound as starting material (Table 2).

When compound **5** was treated with 2 equiv of I_2_, it was observed that, after 13 h of reaction, the starting material disappeared and it was generated an acceptable 25% of compound **15**, where only one position remained unsaturated. If the reaction time is prolonged up to 32 h, the conjugation is extended throughout the entire molecule, and the fully conjugated product **18** is obtained as the main reaction product in 24% yield.

- Synthesis of 3-oxo-γ-costic acid methyl ester (**19**)

**Scheme 4:**
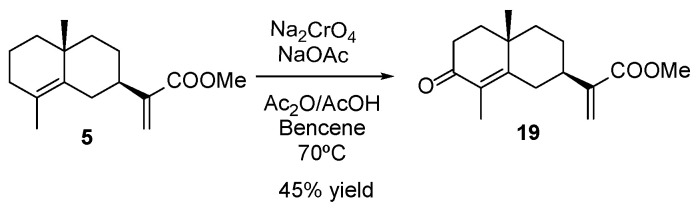
Synthesis of enone **19**.

Once we had in our hands a protocol for the selective dehydration of ilicic acid to generate γ-costic acid, we focused on the synthesis of 3-oxo-γ-costic acid methyl ester (**19**), a natural product used in folk medicine, for which interesting antimicrobial and antiproliferative activities against different human cell lines have been described [26,27]. The only required allylic oxygenation was achieved using Cr(VI) species as oxidant (Scheme 4).

- Epoxidation of **5**. Et_2_AlCl-mediated opening reactions of the corresponding epoxy derivatives

Our group recently reported that the use of alkylaluminium chlorides favoured the opening, cyclization, and rearrangement of epoxypolyprenes [28]. The rationale for this tandem process lies in the fact that once the Lewis acids mediate the oxirane opening, the thus-generated zwitterionic species is shown to possess a metalanion alkoxide, which bears the negative charge and helps the positive charge to persist long enough to evolve to the rearranged products if the process is energetically favourable.

The epoxidation of **5** with *m*CPBA yielded the mixture of epoxides **20**–**21**. The treatment of these epoxides with acids has been described previously by Zaki et al. [29]. When we treated the mixture of diastereomers **20** and **21** with Et_2_AlCl for 6 h at −60 °C, compound **22** was obtained as the major compound—this product was already described by Zaki *et al.*, along with minor quantities of monocyclic **23** (Table 3, entry 1).

**Figure 3:**
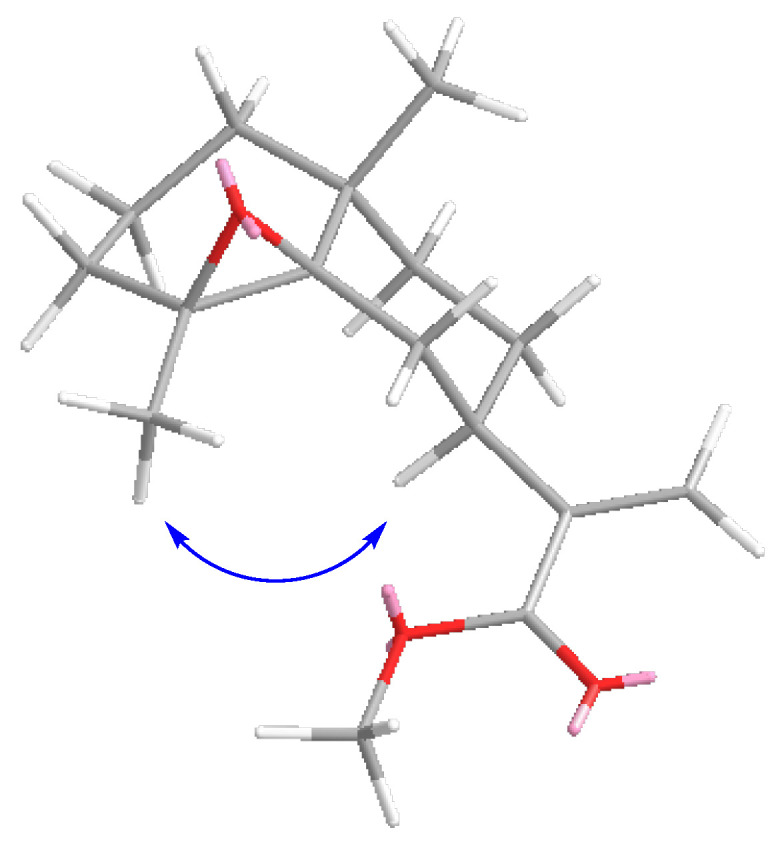
Selected 1D NOE correlation for compound **20**.

Under these experimental conditions, up to 38% of unaltered starting material was also observed. Most notably, this starting material corresponded to only one of the starting epoxides, **20**, as deduced from the NOE effect observed in Me-15 after irradiating H7 (Figure 3).

**Scheme 5:**
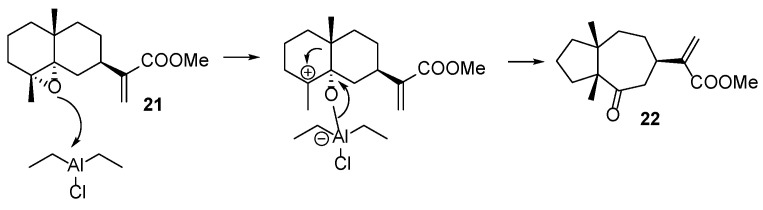
Proposed mechanism for the formation of compound **22**.

This selectivity, which allowed the isolation of one of these epimers for the first time, was rationalized considering the steric hindrance (caused by the methyl group at C10) experienced by the Lewis acid when approaching the β-epoxide **20**. Consequently, product **22** must derive from the α-epoxy derivative **21** (Scheme 5).

When **20** and **21** were treated with Me_2_AlCl for 6 h at −40 °C (3 equiv), both diastereomers reacted, likely due to the smaller size of the reagent (Table 3, entry 2). As a result, apart from **22** and **23**, compounds **24** and **25** were also obtained, the latter ones deriving now from the β-epoxy derivative **20**.

Compound **22** presented an unusual bicyclic structure with cycles of five- and seven-membered rings, bearing two methyl groups at the interannular junctions. This structural moiety is shared with some uncommon neomerane-type sesquiterpenoids [30].

**Figure 4:**
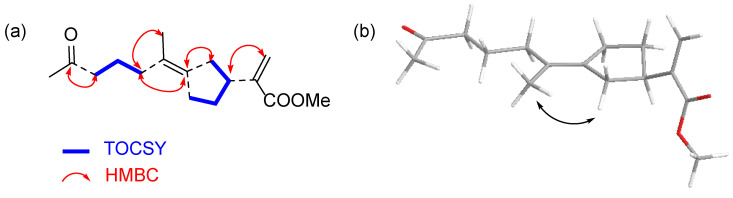
(**a**) Key 1D TOCSY and HMBC correlations for compound **23**. (**b**) Selected 1D NOE correlations for compound **23**.

The molecular formula of compound **23** was assigned as C_16_H_24_O_3_ from its HRMS ([M+H]^+^ at 264.1932), and its complete structure was elucidated using 1D and 2D NMR spectroscopy. These spectra revealed the persistence of a 2-methyl acrylate moiety. The presence of a methyl ketone (^1^H NMR, δ: 2.16, 3H, s; ^13^C NMR, δ: 209.22), and a tetrasubstituted double bond on which a methyl group is located (^1^H NMR, δ: 1.62, 3H, bs; ^13^C NMR, δ: 125.20, C; 135.46, C) was also confirmed. The final planar structure of **23** was deduced from the analysis of its 1D TOCSY spectra and the correlations observed in the HMBC spectrum (Figure 4). Finally, the *E* geometry of the trisubstituted double bond was confirmed by the observation of a NOE effect between the methyl group attached to the double bond and one of the protons on C6.

**Scheme 6:**
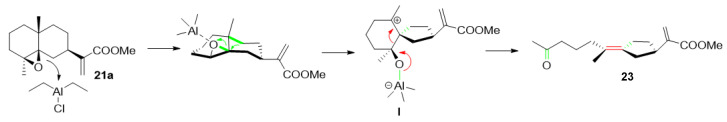
Proposed mechanism for the formation of compound **23**.

The reaction yields obtained (Table 3) suggest that compound **23** originated from epoxide **20**. The cascade process leading to **23**—involving ring opening, contraction of B ring, and subsequent opening of A ring—can be explained by the precise alignment of the C9–C10 bond with the activated C5–O bond (Scheme 6). This alignment facilitates the migration of the C9–C10 bond, triggering the displacement of the leaving group to obtain intermediate **I**. Subsequent cleavage of the O–Al bond, accompanied by the simultaneous migration of the C4–C5 bond, results in the formation of a C5–C10 double bond, ultimately yielding the final product **23**.

The structure of product **24** was also determined by ^1^H and ^13^C NMR spectroscopy. The spectroscopic data agree with the α-costic methyl ester structure (^13^C NMR, δ: 136.2; ^13^C NMR, δ: 126.6; ^1^H NMR, δ: 5.55, 3H, s), with the presence of an oxygenated quaternary carbon at C5 (^13^C NMR, δ: 73.8). The stereochemistry of the molecule could be confirmed by comparison of its NMR spectra with those already described by Zdero et al. [31] for a natural compound isolated from *Apalochlamys spectabilis*. This substance has also been identified by Ceccherelli et al. [32] as a product of the reaction of isoconic acid with SeO_2_. However, the spectroscopic data described by these authors do not agree with those described by Zdero and by us. We can therefore conclude that our obtaining of **24** represents the first synthesis of this natural product.

The NMR data of compound **25** were consistent with a γ-costic acid methyl ester structure possessing an oxygenated methine located at C3 (^1^H NMR, δ: 4.05, bt, *J* = 6.4 Hz); ^13^C NMR, δ: 71.5). The spectroscopical data of **25** matched with those reported by Bohlmann for a natural product isolated from two *Stevia* species [33].

**Scheme 7:**
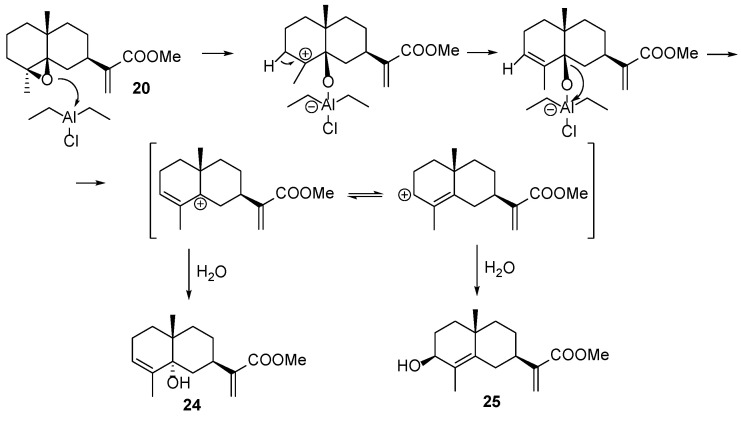
Proposed mechanism for the formation of compounds **24** and **25.**

Since compounds **24** and **25** were obtained only when the β-epoxy derivative **20** was transformed (Table 3 entry 2), the mechanism proposed for the production of these natural products involves the initial opening of this diastereoisomer (Scheme 7).

### 2.3. Nerolidol as a Source of Molecular Diversity

**Scheme 8:**
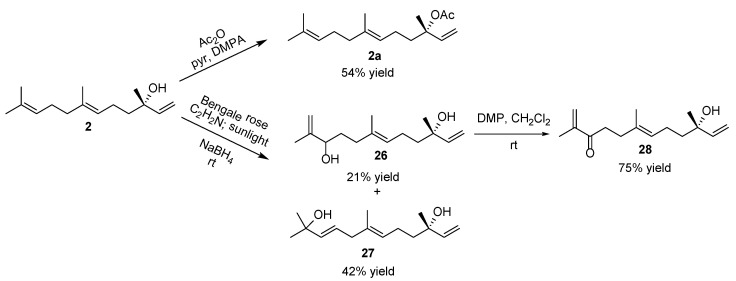
Generation of molecular diversity from nerolidol (**2**).

Nerolidol (**2**) was the second main component of the neutral fraction of *D. viscosa* collected in Granada, Spain. A number of studies have been reported highlighting that this compound may prove beneficial for human health, agriculture, and the food industry [34]. In order to expand structural diversity, we decided to study its acetylation to **2a** and the photosensitized oxidation of this compound using Rose Bengal to generate singlet oxygen (^1^O_2_). Thus, after NaBH_4_ reduction of the corresponding hydroperoxides, the photooxidation of **2** produced the natural diol **27**, together with secondary allylic alcohol **26**. Dess-Martin oxidation of compound **26** led to enone **28** in an acceptable yield (Scheme 8).

### 2.4. 9-Hydroxynerolidol as a Source of Molecular Diversity

9-Hydroxynerolidol (**3**) was the major component of the neutral fraction of *D. viscosa* collected in Granada, Spain. This compound exhibits interesting antifungal properties, and some of us reported that a mixture of **3** and its acetate shows moderate activity against the pest insect *S littoralis* [35]. Considering the availability of this compound on a multigram scale from its natural source and its reported activity, we decided to use 9-hydroxynerolidol as a lead compound in our search for biopesticide compounds.

#### Synthesis of 9-Oxonerolidol (**29**)—Reaction of **29** with Iodine to Produce Diversity

**Scheme 9:**
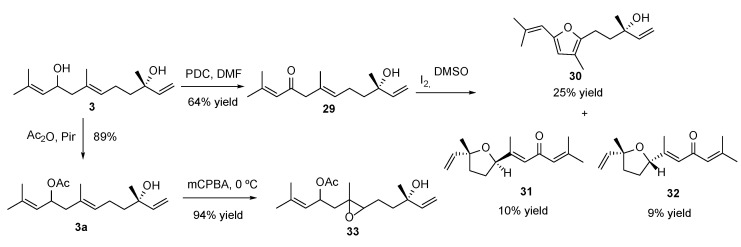
Generation of molecular diversity from 9-hydroxynerolidol (**3**).

9-Oxonerolidol (**29**) is a natural product reported to possess interesting biological activities. Thus, this compound showed activity against antibiotic-resistant *Gram*+ and *Gram*− bacteria, and it is reported to prevent oxidative damage in human lung epithelial cells [36]. 9-Oxonerolidol (**29**) was straightforwardly obtained from compound **2** by PDC-mediated oxidation (Scheme 9).

Reaction of enone **29** with the I_2_-DMSO system led to the generation of monocycles **30**–**32**. The structures of compounds **30**–**32** were elucidated by analysis of their 1D and 2D NMR spectra. The major compound (**30**) shows a molecular peak [M+H]^+^ at *m/z* 235.1691, corresponding to a molecular formula of C_15_H_22_O_2_, indicating an additional degree of unsaturation compared to the starting material. The presence of a disubstituted methylfuran ring was inferred from the analysis of its NMR spectra, which confirmed the presence of a methyl group on a double bond (^1^H: δ 1.95, s, 3H), an olefinic proton (^1^H: δ 5.92, bs) and three quaternary sp^2^ carbon (two of them bonded to the furane oxygen:^13^C: δ 150.9, 148.8, 115.10).

**Scheme 10:**
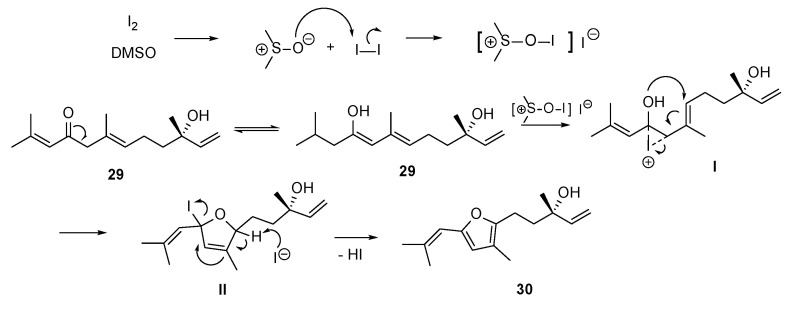
Proposed mechanism for the formation of compound **30.**

Considering our previously reported mechanism for transforming β-methyl enones into furan derivatives [21], we propose the pathway in Scheme 10 for the conversion of **29** into **30** using I_2_/DMSO.

**Figure 5:**
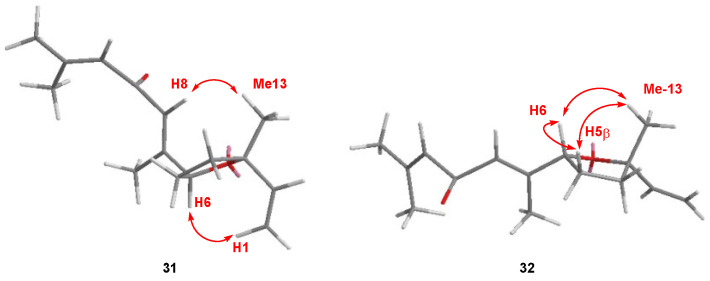
Selected 1D NOE correlations for compounds **31** and **32.**

Compounds **31** and **32** are isomers presenting the molecular formula C_15_H_22_O_2_, as deduced from their HRMS. This data implies again a new degree of unsaturation with respect to the starting material. The presence of two α-carbonyl protons and an oxygenated methine in both molecules supports these assignments (see experimental part). The relative configuration of the stereocenters at C3 and C6 of the tetrahydrofuran ring was determined by NOE experiments (Figure 5).

**Scheme 11:**
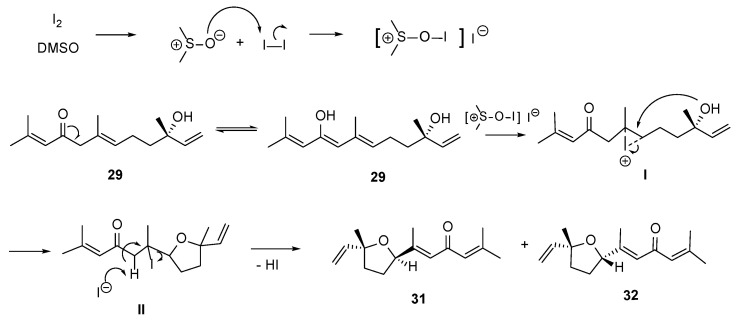
Proposed mechanism for the formation of compounds **31** and **32.**

The mechanism proposed for the formation of **31** and **32** is shown in Scheme 11. The process involves attacks of the iodoxydimethylsulfonium iodide on the 6–7 double bond, with concomitant addition of the tertiary hydroxyl group to the iodonium intermediate species **I**, thus generating the tetrahydrofuran **II**. Subsequent HI elimination produces a mixture of stereoisomers (Scheme 11).

### 2.5. Plant Protection Effects

The plant protection effects of a selection of the compounds originated in this study were tested against insect pests (*S. littoralis*, *M. persicae*, *R. padi*), the root-knot nematode *M. javanica*, and their phytotoxicity on the plants’ ryegrass and lettuce.

Among the compounds tested on insect pests, nerolidol derivatives **3a** (EC_50_ of 8 μg/cm^2^ on *R. padi*), **27** (EC_50_ of 10 and 20 μg/cm^2^ on *M. persicae* and *R. padi*), **28** (EC_50_ of 15 and 12 μg/cm^2^ on *M. persicae* and *R. padi*), and **29** (EC_50_ of 21 μg/cm^2^ on *R. padi*) were aphid antifeedants with some effective doses 2–1.5 times more active than the positive control thymol (**3a** and **28** on *R. padi*). Among ilicic acid derivatives, **11** (EC_50_ = 25 and 11 μg/cm^2^ on *S. littoralis* and *M. persicae*) was an effective antifeedant, with similar or 1.7 stronger potencies against *S. littoralis* and *R. padi*, followed by **4** (EC_50_ of 16 μg/cm^2^ on *R. padi*) (Table 4).

The tested compounds showed limited nematicidal effects against the root-knot nematode *M. javanica*, with compound **27** (a nerolidol derivative) and compound **11** (an ilicic acid derivative) being the most active (MLC of 0.5 and LD_50_,_90_ of 0.31 and 0.59 ug/uL respectively), followed by **4** which presented moderate-low effects (61% mortality at 1 ug/uL) (Table 5). The activity of **28** and **11** is two times below the positive control thymol.

**Figure 6:**
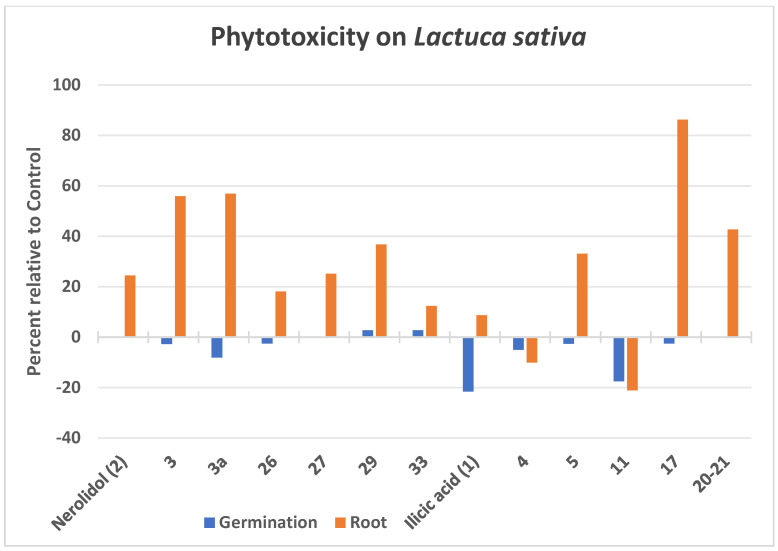
Phytotoxicity (% relative to the control) of the compounds tested (nerolidol (**2**), ilicic acid and derivatives **3**, **3a**, **26**–**29**, **33**, **4**, **5**, **11**, **17**, and **20**–**21**) against *Lactuca sativa*. The dose tested was 0.1 mg/mL, and lower (0.05) for the active compounds (until the effects are < 50%).

**Figure 7:**
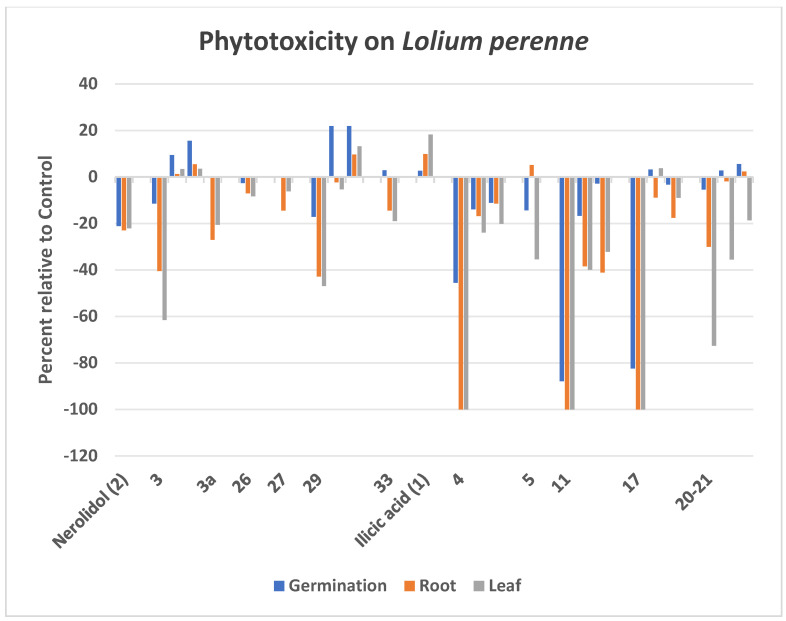
Phytotoxicity (% relative to the control) of the compounds tested (nerolidol (**2**), ilicic acid and derivatives **3**, **3a**, **26**–**29**, **33**, **4**, **5**, **11**, **17**, and **20**–**21**) against *Lolium perenne*. The doses tested were 0.1, 0.05, and 0.025 mg/mL for the active compounds.

These compounds were tested for phytotoxic effects against two model plant species, the monocotyledoneous ryegrass and the dycotiledoneous lettuce (*L. perenne* and *L. sativa*) to assess unwanted phytotoxicity (on lettuce) and potential herbicidal effects (on ryegrass) (Figure 6 and Figure 7, Table S1).

The phytotoxicity observed was selective. *L. sativa* was not affected significantly by any of them (% inhibition < 50% for all parameters), with ilicic acid **1** inhibiting root growth by 21%, while **17, 3**, and **3a** promoted root growth with respect to the control (86.3% and 55–57% promotion, respectively) (Figure 6).

Figure 7 shows the phytotoxic effects of the test compounds on ryegrass. Overall, the nerolidol derivatives were shown not to be phytotoxic to this plant except for **3** (61% leaf growth inhibition), while significant effects were observed for the ilicic acid-related compounds. Specifically, **4**, **11**, **17** (with >801% inhibition of all parameters at the maximum dose) were the most effective, followed by **20**–**21** that inhibited leaf growth (73%).

### 2.6. Ixodicidal Effects

Nerolidol (**2**), ilicic acid (**1**), and compounds **3**, **3a**, **29**, **33, 4**, **5**, **11**, and **17** were tested against larvae of the tick *Hyalomma lusitanicum.* All these compounds were active except **2, 1**, and **4**. Their lethal doses LD_50_ ranged between 0.62–2.61 μg/mg, all within the range of the positive control thymol, except **29**, the most active derivative (LD_50,90_ = 0.62, 1.09 μg/mg), five times over the positive control thymol (Table 6).

Nerolidol (**2**) was not antifeedant against the insect species tested; therefore, the chemical transformations carried out on this compound improved the activity of **3a** on *R. padi*, **27** on *M. persicae* and *R. padi*, **28** on *M. persicae* and *R. padi* and **29** on *R. padi*. In the case of illicic acid (**1**) derivatives, lactone **11** was active on all the targets (except *M. persicae*), followed by **4** and **5** with effects on *R. padi*, while **1** was inactive. Therefore, the synthetic strategy applied here yielded improved antifeedant nerolidol and ilicic acid derivatives. Only one active nematicidal derivative for each chemical class was obtained (**28** and **11**). Since both nerolidol and ilicic acid lacked nematicidal action, we can consider these molecules as potential nematicidal leads. The ilicic acid derivatives **4**, **11**, **17** showed strong herbicidal effects on *L. perenne*, while ilicic acid **1** was inactive, suggesting that these molecules could be herbicidal leads. All nerolidol derivatives tested were effective ixodicidal agents with a clear improvement in the case of 9-oxonerolidol (**29**), with an efficient dose—five times above the positive control thymol. Similarly, compounds **5**, **11**, and **17** showed improved acaricidal effects compared to the inactive ilicic acid **1**.

Nerolidol, a sesquiterpene alcohol, exhibits a range of effects on insects, acting as a repellent and even impacting their growth and development. Nerolidol has aphicidal effects against *Metopolodium dirhodum* [39], larvicidal action against *Plutella xylostella* [40], and impairs the normal growth, development, and reproduction of *Spodoptera exigua*, acting as an analog of juvenile hormone JH [41]. Similarly, nerolidol exhibited growth-inhibitory effects on larvae of the melon fruit fly (*Zeugodacus cucurbitae*) [42]. It was also shown to be an effective repellent of females of the mosquito *Aedes aegypti* [43]. In this work, we found moderate antifeedant effects of nerolidol on *M. persicae* (61% SI). Similarly, (*Z*)-nerolidol showed pre-ingestive, ingestive, and post-ingestive deterrent activities against *M. persicae* [44]. However, we did not find any antifeedant effects against *S. littoralis* or *R. padi*, suggesting species-dependent antifeedant effects for this compound. Nerolidol has been described as acaricidal against *Tetranychus urticae* [45] and reported as larvicidal to the tick *Rhipicephalus microplus* [46]. However, in this work, nerolidol was not larvicidal to the tick species *H. lusitanicum*.

Nerolidol has also been described as phytotoxic to *Arabidopsis thaliana*, causing alterations in root morphology, bringing changes in auxin balance, inducing changes in sugar, amino acid, and carboxylic acid profiles, and increasing the levels of H_2_O_2_ and MDA in root tissues in a dose-dependent manner [47]. However, in this work, nerolidol did not affect *L. sativa* and showed low negative effects on *L. perenne*

A similar case was observed with ilicic acid, a compound reported as antifeedant to the stored-product pests *Tenebrio molitor* [48] and the beetle *Leptinotarsa decemlineata*. However, it proved to be ineffective against *S. littoralis* [9] or the aphids tested here. There are no reports on the acaricidal effects of ilicic acid.

Ilicic acid has reported phytotoxic effects on the roots of cauliflower, cress, and radish at 0.025 mg/mL [49]. Here, we found moderate negative effects on *L. sativa* germination (22% inhibition at 0.1 mg/mL) while being inactive on the ryegrass.

When compared with existing commercial biopesticides (e.g., azadirachtin, pyrethrins, spinosad, and essential-oil-based formulations), the synthetic derivatives reported here represent an advantage. Unlike many natural biopesticides that often suffer from narrow-spectrum efficacy, the nerolidol and ilicic acid derivatives studied here demonstrate multi-target activity across insects, nematodes, ticks, and weeds, while also showing potential as herbicidal leads. This versatility is particularly valuable in the context of Integrated Pest Management (IPM), where combining bioactive compounds that act at different trophic levels can reduce reliance on synthetic pesticides.

From the perspective of sustainable agriculture, the direct derivatization of abundant natural products such as nerolidol and ilicic acid offers a dual advantage: valorization of renewable plant resources like *D. viscosa* and generation of molecules with enhanced potency and selectivity, which can be more efficiently integrated into eco-friendly crop protection strategies.

## 3. Materials and Methods

### 3.1. Instruments and Chemicals

Column chromatography was carried out with silica gel SDS 60 (35−70 μm). Thin-layer chromatography on 0.25 mm E. Merck silica gel plates (60F-254) (Sigma Aldrich, St. Louis, MO, USA) was employed to monitor reactions, the fractionation of *D. viscosa* MTBE extract, and the isolation of natural products by chromatography using UV light and a solution of phosphomolybdic acid in ethanol as the visualizing agent. Semipreparative HPLC separation was carried out on a column (5 μm Silica, 10 × 250 mm^2^) at a flow rate of 4.0 mL/min in an Agilent Series 1100 instrument. NMR spectra were recorded with BRUKER Avance NEO (^1^H NMR 600 MHz/^13^C NMR 150 MHz), Varian Direct Drive (^1^H NMR 500 MHz/^13^C NMR 125 MHz), and BRUKER Nanobay Avance III HD (^1^H NMR 400 MHz) spectrometers (Bruker, Billerica, MA, USA. The signals multiplicity is indicated by the following abbreviations: s = singlet, d = doublet, t = triplet, q = quartet, quint = quintuplet, hex = hexuplet, hep = heptuplet, bs = broad singlet, bd = broad doublet, bt = broad triplet, dd = doublet of doublets, dt = doublet of triplets, dq = doublet of quartets, dquint = doublet of quintets, td = triplet of doublets, ddd = doublet of doublets of doublets, m = multiplet. High-resolution mass spectra (HRMS) were determined on a BRUKER Autoflex mass spectrometer (Bruker, Billerica, MA, USA).

### 3.2. Plant Material, Extraction, and Isolation of Natural Products

Specimens of *Dittrichia viscosa* (GDA54164) were collected in Granada (37.208108, −3.622045, Spain) in April 2023. Aerial parts of *D. viscosa* (2600 g) were extracted by maceration two times for 30 min each, using MTBE as solvent, and finally obtained 19.8 g of extract (0.76% yield from fresh plant).

MTBE extract was then fractionated using 2N NaOH as a base. The process began by dissolving 10 g of the extract in 200 mL of MTBE, then the solution was extracted with 100 mL 2N NaOH solution three times in a separating funnel with vigorous shaking. Afterward, the combined aqueous phases were separated from the organic phase. The aqueous phase was then acidified to pH 2 using 2N HCl and re-extracted three times for three minutes each with 100 mL of TBME to yield 6.75 g. This residue was crystallized from MTBE to give 4.65 g of ilicic acid (**1**). The organic phase was also chromatographed employing mixtures of hexane (H) and ethyl acetate (EtOAc) of increasing polarity to give 364 mg of nerolidol (**2**) (H:EtOAc, 10:1) and 775 mg of 9-hydroxinerolidol (**3**) (H:EtOAc, 2:1).

We started this approach by studying different ways of extracting the aerial parts of the plant. After some experimentation using different solvents and conditions, we selected the maceration of the plant material twice for 30 min with *tert*-butylmethylether (MTBE). This procedure led to an extract yield of about 5% on dry plant weight or 0.76% of fresh plant.

### 3.3. Synthesis

#### 3.3.1. Esterification of Ilicic Acid

To a solution of ilicic acid (**1**) (1500 mg, 5.94 mmol) in a mixture of benzene:MeOH in a 4:1 ratio (30 mL), TMSCHN_2_ (3.6 mL of a 2M solution in diethylether, 7.13 mmol) was added under an argon atmosphere at 0 °C. This mixture was stirred for 30 min until the starting material was consumed, at which point the mixture was evaporated under vacuum. Product **4** was purified by flash chromatography (H:MTBE, 1:1) to obtain 1510 mg (95% yield).

#### 3.3.2. Dehydration of Compound **4**

To a solution of **2** (500 mg, 1.88 mmol) in toluene (35 mL), I_2_ (483 mg, 1.88 mmol) was added under an argon atmosphere at 111 °C (reflux). This mixture was stirred for 10 h. After completing the reaction, 80 mL of 5% NaHSO_3_ was added and stirred for an additional 10 min. Subsequently, 100 mL of MTBE was added, and the mixture was washed with water and then with brine, dried over anhydrous Na_2_SO_4_, and evaporated under reduced pressure. Finally, products **5**, **6**, and **7** were obtained with a 75% yield in a 5:1:2 ratio by silica gel impregnated with 20% AgNO_3_ column chromatography.

#### 3.3.3. Selective Dehydration of Compound **4**

To a solution of **4** (1485 mg, 5.57 mmol) in benzene (105 mL), I_2_ (1440 mg, 5.61 mmol) was added under an argon atmosphere at room temperature. This mixture was stirred for 4 h 30 min. Then, 80 mL of 5% NaHSO_3_ was added and stirred for an additional 10 min. Subsequently, 100 mL of MTBE was added, and the mixture was washed with water and then with brine, dried over anhydrous Na_2_SO_4_, and evaporated under reduced pressure. Finally, product **5** was obtained with an 85% yield.

#### 3.3.4. Reaction of Compound **4** with I_2_ and DMSO

To a solution of 4 (763 mg, 2.87 mmol) in toluene (30 mL), I_2_ (153 mg, 0.60 mmol) and DMSO (3.5 mL, 49 mmol) were added under an argon atmosphere at reflux. This mixture was stirred for 18 h. After completing the reaction, 100 mL of MTBE was added, and the mixture was washed with a solution of saturated Na_2_SO_3_ (3 × 40 mL) and brine (2 × 40 mL). Later, it was dried over anhydrous Na_2_SO_4_ and evaporated under reduced pressure. Finally, products **5**–**10** were obtained. Column chromatography technique was employed using as eluent a mixture of H:MTBE in different proportions to obtain 500 mg (60% yield) of a mixture composed of compounds **5**–**7** and 160 mg of compounds **8**–**10** in a 2:2:3 ratio. Eventually, compounds **8**–**10** were isolated by HPLC using H:Et_2_O (9:1) as eluent, with retention times of 14, 15, and 18 min, respectively.

Compound **9**. ^1^H NMR: (400 MHz, CDCl_3_): δ 8.04 (s, 1H), 7.82 (d, *J* = 8.5 Hz, 1H), 7.70 (d, *J* = 8.3 Hz, 1H), 7.52 (bd, *J* = 8.3, 1H), 7.36 (dd, *J* = 8.5, 7.1 Hz, 1H), 7.33 (d, *J* = 7.1 Hz, 1H), 6.46 (bs, *J* = 1.2 Hz, 1H), 6.02 (bs, 1H), 3.86 (s, 3H), 2.70 (s, 3H). ^13^C NMR (100 MHz, CDCl_3_): δ 167.4 (C), 141.7 (C), 134.6 (C), 134.1 (C), 133.1 (C), 132.3 (C), 128.2 (CH), 127.2 (CH), 127.0 (CH), 126.1 (CH), 126.1 (CH), 125.8 (CH), 123.8 (CH_2_), 52.3 (CH_3_), 19.4 (CH_3_). HRMS TOF (ES^+^) *m/z* calculated for C_15_H_15_O_2_ [M+H]^+^ 227.1072, found 227.1074.

Compound **10**. 1H NMR: (400 MHz, CDCl_3_): δ 10.39 (s, 1H), 9.33 (bs, 1H), 8.10 (d, *J* = 8.1 Hz, 1H), 8.01 (dd, *J* = 7.1, 1.3 Hz, 1H), 7.91 (d, *J* = 8.4 Hz, 1H), 7.66–7.63 (m 2H), 6.52 (d, *J* = 1.1 Hz, 1H), 6.10 (d, *J* = 1.2 Hz, 1H), 3.87 (s, 3H). ^13^C NMR (100 MHz, CDCl_3_): δ 193.5 (C), 167.2 (C), 145.0 (C), 141.2 (C), 137.2 (CH), 134.9 (CH), 133.6 (C), 133.3 (C), 128.5 (CH_2_), 128.1 (CH), 127.5 (CH), 126.3 (C), 125.3 (CH), 124.6 (CH), 52.4 (CH_3_). HRMS TOF (ES^+^) *m/z* calculated for C_15_H_13_O_3_ [M+H]^+^ 241.0865, found 241.0861.

#### 3.3.5. Reaction of Compound **4** with I_2_ and Toluene: MeOH (16 h)

To a solution of compound **4** (600 mg, 2.25 mmol) in a mixture of toluene:MeOH in a 9:1 ratio (20 mL), I_2_ (1144 mg, 4.51 mmol) was added under an argon atmosphere, and the mixture was refluxed. The reaction was stirred for 16 h and then evaporated under vacuum. The reaction crude was purified by silica gel impregnated with 20% AgNO_3_ column chromatography (H:MTBE, 95:5) to obtain 220 mg (40%) of **5**, 40 mg (9%) of **11**, 90 mg (19%) of **12**, and 90 mg (17%) of **13**.

#### 3.3.6. Reaction of Compound **4** with I_2_ and Toluene: MeOH (38 h)

To a solution of compound **4** (500 mg, 1.88 mmol) in a mixture of toluene: MeOH 9:1 (10 mL), I_2_ (950 mg, 3.75 mmol) was added under an argon atmosphere at reflux. This mixture was stirred for 38 h and then evaporated under vacuum. Products **5**, **8**, **11**, **12**, **13** and **14** were purified by column chromatography using as eluent a mixture of H:MTBE of increasing polarity to obtain 110 mg (24%) of **5**, 40 mg (10%) of **11**, 100 mg (22%) of **14**, 45 mg (10%) of **15**, 30 mg (7%) of **16** and 50 mg (12%) of **17**.

Compound **12**. ^1^H NMR (400 MHz, CDCl_3_): δ 7.06 (d, *J* = 7.6 Hz, 1H), 7.02 (d, *J* = 7.6 Hz, 1H), 5.92 (s, 1H), 3.09 (m, 1H), 3.06 (m, 1H), 2.74 (m, 1H), 2.60 (m, 1H), 2.51 (s, 3H), 2.20 (s, 3H), 1.90 (s, 3H). ^13^C NMR (100 MHz, CDCl_3_): δ 174.6 (C), 161.0 (C), 136.9 (C), 135.0 (C), 133.7 (C), 132.4 (C), 130.3 (CH), 129.0 (CH), 120.7 (C), 79.4 (CH), 30.1 (CH2), 23.8 (CH2), 20.6 (CH_3_), 20.1 (CH_3_), 8.4 (CH_3_). HRMS TOF (ES^+^) *m/z* calculated for C_15_H_17_O_2_ 229.1229 [M+H]^+^, found 229.1233.

Compound **13**. ^1^H NMR: (400 MHz, CDCl_3_): δ 7.08 (d, *J* = 7.9 Hz, 1H), 7.04 (d, *J* = 7.9 Hz, 1H), 3.24 (s, 3H), 3.12 (ddd, *J* = 16.4, 7.3, 1.9 Hz, 1H), 2.98 (ddd, *J* = 12.9, 6.6, 2.0 Hz, 1H), 2.78 (m, 1H), 2.66 (m, 1H), 2.61 (s, 3H), 2.21 (s, 3H), 1.96 (s, 3H). ^13^C NMR (100 MHz, CDCl_3_): δ δ 171.8 (C), 158.3 (C), 136.6 (C), 134.5 (C), 133.5 (C), 133.3 (C), 130.9 (CH), 130.3 (CH), 123.9 (C), 105.5 (C), 50.1 (CH_3_), 29.3 (CH_2_), 21.7 (CH_2_), 20.6 (CH_3_), 20.0 (CH_3_), 8.3 (CH_3_). HRMS TOF (ES^+^) *m/z* calculated for C_16_H_19_O_2_ [M+H]^+^ 243.1385, found 243.1383.

Compound **15**. ^1^H NMR: (400 MHz, CDCl_3_): δ 7.98 (d, *J* = 8.7 Hz, 1H), 7.88 (d, *J* = 1.9 Hz, 1H), 7.49 (dd, *J* = 8.7, 1.9 Hz, 1H), 7.21 (d, *J* = 7.3 Hz, 1H), 7.18 (d, *J* = 7.3 Hz, 1H), 3.93 (q, *J* = 7.2 Hz, 1H), 3.67 (s, 3H), 2.66 (s, 3H), 2.64 (s, 3H), 1.61 (d, *J* = 7.2 Hz, 3H). ^13^C NMR (100 MHz, CDCl_3_): δ 175.2 (C), 137.6 (C), 132.87 (C), 132.4 (C), 132.3 (C), 132.0 (C), 126.8 (CH), 126.3 (CH), 125.3 (CH), 125.1 (CH), 123.3 (CH), 52.2 (CH_3_), 46.0 (CH), 19.5 (CH_3_), 19.5 (CH_3_), 18.9 (CH_3_). HRMS TOF (ES^+^) *m/z* calculated for C_16_H_19_O_2_ [M+H]^+^ 243.1385, found 243.1382.

Compound **16**. ^1^H NMR: (400 MHz, CDCl_3_): δ 6.09 (s, 1H), 5.53 (s, 1H), 4.53 (dd, *J* = 5.2, 3.6 Hz, 1H), 2.87 (m, 1H), 1.87–1.79 (m, 2H), 1.74–1.48 (m, 4H), 1.40 (dt, *J* = 13.3, 3.5 Hz, 1H), 1.33 (bd, *J* = 13.1 Hz, 1H), 1.17 (td, *J* = 13.5, 3.7 Hz, 1H), 1.08 (td, *J* = 13.3, 4.2 Hz, 1H), 1.00 (qd, *J* = 12.7, 4.6 Hz, 1H), 0.95 (s, 3H), 0.94 (d, *J* = 5.9 Hz, 1H), 0.93 (m, 1H). ^13^C NMR (100 MHz, CDCl_3_): δ 170.6 (C), 142.4 (C), 119.6 (CH2), 76.9 (CH), 51.5 (CH), 42.4 (CH_2_), 40.4 (CH), 39.4 (CH_2_), 37.0 (CH2), 32.4 (C), 28.5 (CH), 24.6 (CH_2_), 21.4 (CH_2_), 19.5 (CH_3_), 18.3 (CH_3_). HRMS TOF (ES^+^) *m/z* calculated for C_15_H_23_O_2_ [M+H]^+^ 235.1698, found 235.1702.

Compound **17**. ^1^H NMR: (400 MHz, CDCl_3_): δ 7.81 (d, *J* = 8.5 Hz, 1H), 7.37 (d, *J* = 8.5 Hz, 1H), 7.22 (d, *J* = 7.2 Hz, 1H), 7.18 (d, *J* = 7.4 Hz, 1H), 3.85 (q, *J* = 7.6 Hz, 1H), 2.86 (s, 3H), 2.64 (s, 3H), 1.65 (d, *J* = 7.6 Hz, 3H). ^13^C NMR (100 MHz, CDCl_3_): δ 178.9 (C), 150.5 (C), 134.3 (C), 132.3 (C), 131.5 (C), 128.4 (CH), 127.4 (CH), 124.0 (C), 121.3 (CH), 120.4 (C), 120.3 (CH), 38.6 (CH), 22.9 (CH_3_), 20.1 (CH_3_), 16.2 (CH_3_). HRMS TOF (ES+) *m/z* calculated for C_15_H_15_O_2_ [M+H]^+^ 227.1072, found 227.1076.

#### 3.3.7. Reaction of Compound **5** with I_2_ Using Toluene: MeOH (13 h)

To a solution of product **5** (232 mg, 0.935 mmol) in a mixture of toluene:MeOH 9:1 (10 mL), I_2_ (237 mg, 0.94 mmol) was added under an argon atmosphere at reflux. This mixture was stirred for 13 h. After finishing the reaction, 50 mL of 5% NaHSO_3_ was added and stirred for an additional 10 min. Later, 50 mL of MTBE was added, and the mixture was washed with water and then with brine, dried using anhydrous Na_2_SO_4,_ and evaporated under vacuum. Products **11**, **12**, **15**, and **17** were purified by silica gel impregnated with 20% AgNO_3_ column chromatography (H:MTBE, 95:5) to obtain 20 mg (9%) of **11**, 50 mg (25%) of **12**, 30 mg (14%) of **15**, and 20 mg (10%) of **17**.

#### 3.3.8. Reaction of Compound **5** with I_2_ Using Toluene: MeOH (32 h)

To a solution of product **5** (1150 mg, 4.63 mmol) in a mixture of toluene:MeOH 9:1 (50 mL), I_2_ (2350 mg, 9.27 mmol) was added under an argon atmosphere. The resulting mixture was heated at reflux for 32 h. After finishing the reaction, 250 mL of 5% NaHSO_3_ was added and stirred for an additional 10 min. Then, 250 mL of MTBE was added, and the mixture was washed with water and then with brine, dried using anhydrous Na_2_SO_4_, and evaporated under vacuum. Products **11**, **12**, **13**, and **18** were purified by silica gel impregnated with 20% AgNO3 column chromatography (H:MTBE, 95:5) to obtain 60 mg (6%) of **11**, 80 mg (8%) of **12**, 80 mg (7%) of **13**, 260 mg (24%) of **18**.

Compound **18**. ^1^H NMR: (400 MHz, CDCl_3_): δ 8.05 (s, 1H), 7.99 (d, *J* = 8.7 Hz, 1H), 7.56 (d, *J* = 8.7 Hz, 1H), 7.21 (s, 2H), 6.45 (s, 1H), 6.02 (s, 1H), 3.86 (s, 3H), 2.67 (s, 3H, 2.66 (s, 3H). ^13^C NMR (100 MHz, CDCl_3_): δ 167.5 (C), 141.7 (C), 133.7 (C), 132.7 (C), 132.4 (C), 132.3 (C), 132.2 (C), 126.7 (CH), 126.7 (CH), 125.5 (CH), 124.4 (CH), 124.4 (CH), 122.2 (CH2), 52.2 (CH_3_), 19.4 (CH_3_), 19.3 (CH_3_). HRMS TOF (ES^+^) *m/z* calculated for C_16_H_17_O_2_ [M+H]^+^ 241.1229, found 241.1231.

#### 3.3.9. Oxidation of Compound **5**

To a solution of **5** (148 mg, 0.60 mmol) in benzene (14 mL), Na_2_CrO_4_ (290 mg, 1.79 mmol), NaOAc (147 mg, 1.79 mmol), Ac_2_O (1 mL, 0.019 mmol), and AcOH (2 mL, 0.023 mmol) were added under an argon atmosphere at 70 °C. This mixture was stirred for 8 h. After completing the reaction, 100 mL of MTBE was added, and the mixture was washed with distilled water (3 × 30 mL) and brine (3 × 30 mL). Finally, the mixture was dried over Na_2_SO_4_ and evaporated under reduced pressure. Compound **19** (70 mg, 45% yield) was obtained by column chromatography using as eluent H:MTBE (4:1).

#### 3.3.10. Epoxidation of Compound **5**

To a solution of compound **5** (925 mg, 3.72 mmol) in DCM (70 mL), *m*-chloroperbenzoic acid (868 mg, 5.0 mmol) was added under an argon atmosphere at room temperature. This mixture was stirred for 15 min After this period, the reaction mixture was washed with 50 mL of saturated Na_2_SO_3_ solution, and then with saturated Na_2_S_2_O_5_ (2 × 50 mL) and finally with a saturated NaHCO_3_ solution (3 × 50 mL). The organic phase was subsequently dried over anhydrous Na_2_SO_4_, and the solvents were removed under vacuum. Compounds **20** and **21** were obtained in a 4:3 ratio (732 mg, 75%).

#### 3.3.11. Reaction of Epoxides **20** and **21** with Et_2_AlCl

To a solution of a mixture composed of **20** and **21** (100 mg, 0.38 mmol) in DCM (38 mL), Et_2_AlCl (0.75 mL, 0.75 mmol) was gradually added, dissolved in DCM (12 mL) under argon atmosphere at −70 °C. This mixture was stirred for 6 h. After this period, 2.2 mL of a solution composed of 1.5 mL of Et_3_N and 0.7 mL of a MeOH:H_2_O (4:1) mixture was added to the reaction. Then 50 mL of DCM was added, and the reaction mixture was washed three times with 60 mL of a saturated solution of NH_4_Cl (3 × 50 mL), and brine (3 × 50 mL). The organic phase was subsequently dried over anhydrous Na_2_SO_4_, and the solvents were removed under vacuum. Products **20**, **22**, and **23** were purified by column chromatography using as eluent H:Et_2_O (5:1) to obtain 38 mg of **20**, 39 mg (39%) of **22**, and 4 mg (4%) of **23**.

Compound **20**. ^1^H NMR (400 MHz, CDCl_3_): δ 6.16 (s, 1H), 5.55 (s, 1H), 3.74 (s, 3H), 2.53 (t, *J* = 12.4 Hz, 1H), 1.87–1.66 (m, 5H), 1.58–1.27 (m, 6H), 1.34 (s, 3H), 1.05 (s, 3H), 1.04–1.98 (m, 1H). ^13^C NMR (100 MHz, CDCl_3_): δ 167.5 (C), 144.5 (C), 122.8 (CH_2_), 68.5 (C), 64.2 (C), 51.8 (CH_3_), 38.7 (CH), 36.0 (CH_2_), 35.5 (CH_2_), 33.8 (C), 32.4 (CH_2_), 31.2 (CH_2_), 27.5 (CH_2_), 23.0 (CH_3_), 21.4 (CH_2_), 16.7 (CH_2_).

Compound **22**. ^1^H NMR (400 MHz, CDCl_3_): δ 6.15 (s, 1H), 5.55 (s, 1H), 3.77 (s, 3H), 2.95 (*p*, *J* = 9.2 Hz, 1H), 2.59 (dd, *J* = 15.9, 7.3 Hz, 1H), 2.39 (t, *J* = 7.4 Hz, 2H), 2.42–2.33 (m, 1H), 2.24–2.11 (m, 1H), 2.13 (s, 3H), 2.06–1.95 (m, 1H), 1.99 (t, *J* = 7.4 Hz, 2H), 1.66 (*p*, *J* = 7.4 Hz, 2H), 1.56 (s, 3H), 1.48–1.55 (m, 1H). ^13^C NMR (100 MHz, CDCl_3_): δ 209.2 (C), 167.9 (C), 143.6 (C), 135.5 (C), 125.2 (C), 122.4 (CH_2_), 51.8 (CH_3_), 43.3 (CH_2_), 41.0 (CH), 36.8 (CH_2_), 34.3 (CH_2_), 31.9 (CH_2_), 29.9 (CH_3_), 29.5 (CH_2_), 21.8 (CH_2_), 18.5 (CH_3_). HRMS TOF (ES+) *m/z* calculated for C_16_H_25_O_3_ [M+H]^+^ 265.1804, found 265.1801.

Compound **23**. ^1^H NMR (400 MHz, CDCl_3_): δ 6.15 (s, 1H), 5.55 (s, 1H), 3.77 (s, 3H), 2.95 (*p*, *J* = 9.2 Hz, 1H), 2.59 (dd, *J* = 15.9, 7.3 Hz, 1H), 2.39 (t, *J* = 7.4 Hz, 2H), 2.42–2.33 (m, 1H), 2.24–2.11 (m, 1H), 2.13 (s, 3H), 2.06–1.95 (m, 1H), 1.99 (t, *J* = 7.4 Hz, 2H), 1.66 (*p*, *J* = 7.4 Hz, 2H), 1.56 (s, 3H), 1.48–1.55 (m, 1H). ^13^C NMR (100 MHz, CDCl_3_): δ 209.2 (C), 167.94 (C), 143.6 (C), 135.5 (C), 125.2 (C), 122.4 (CH_2_), 51.8 (CH_3_), 43.3 (CH_2_), 41.0 (CH), 36.8 (CH_2_), 34.3 (CH_2_), 31.9 (CH_2_), 29.9 (CH_3_), 29.5 (CH_2_), 21.8 (CH_2_), 18.5 (CH_3_). HRMS TOF (ES^+^) *m/z* calculated for C_16_H_25_O_3_ [M+H]^+^ 265.1804, found 265.1806.

#### 3.3.12. Reaction of Epoxides **20** and **21** with Me_2_AlCl

To a solution of a mixture composed of **20** and **21** (110 mg, 0.42 mmol) in DCM (40 mL), Me_2_AlCl (0.83 mL, 0.83 mmol) was gradually added, dissolved in DCM (10 mL) under argon atmosphere at −40 °C. This mixture was stirred for 6 h. After this period, 2.2 mL of a solution composed of 1.5 mL of Et_3_N and 0.7 mL of a MeOH:H_2_O (4:1) mixture was added to the reaction. Then 50 mL of DCM was added, and the reaction mixture was washed three times with 60 mL of a saturated solution of NH_4_Cl (3 × 50 mL), and brine (3 × 50 mL). The organic phase was subsequently dried over anhydrous Na_2_SO_4_, and the solvents were removed under vacuum. Products **22**, **23**, **24**, and **25** were isolated by column chromatography using as eluent H:Et_2_O (5:1) to obtain 45 mg (41%) of **22**, 2 mg (2%) of **23**, 27 mg (24%) of **24**, and 13 mg (12%) of **25**.

#### 3.3.13. Acetylation of **2**

To a solution of **2** (190 mg, 0.85 mmol) in pyridine (5 mL) and DMPA (10 mg), Ac_2_O (1 mL, 10.6 mmol) was added under an argon atmosphere, and the resulting mixture was heated at reflux for 12 h. After completing the reaction, 20 mL of ice was added, and then 30 mL of MTBE. When the ice melted, the organic phase was washed with HCl 2N (4 × 15 mL), a saturated solution of NaHCO_3_ (3 × 15 mL), followed by distilled water (2 × 15 mL) and brine (3 × 15 mL). Finally, the mixture was dried over Na_2_SO_4_ and evaporated under reduced pressure. Compound **2a** (123 mg, 54% yield) was obtained by column chromatography using as eluent H:MTBE (9:1).

#### 3.3.14. Photooxygenation of Compound **2**

To a solution of **2** (100 mg, 0.45 mmol) in acetonitrile (9 mL) placed in a crystallizer, Bengal Rose (61 mg, 0.06 mmol) was added. The reaction mixture was stirred and allowed to react in sunlight at room temperature. After 15 min, the solvent was evaporated. The reaction crude was dissolved in MeOH (6 mL), and NaBH_4_ (44 mg) was added. The reaction mixture was stirred for 1 h at room temperature. After completing the reduction, MeOH was evaporated, and 20 mL of distilled water was added. Finally, the reaction products were extracted from the aqueous phase using TBME (3 × 20 mL). Compounds **26** (22 mg, 21%) and 27 (44 mg, 42%) were obtained by column chromatography using as eluent H:AcOEt (6:1).

Compound **26** (isolated as a 1:1 mixture of diastereomers). ^1^H NMR (400 MHz, CDCl_3_): δ 5.84 (dd, *J* = 17.3, 10.7 Hz, 1H), 5.15 (dd, *J* = 17.3, 1.3 Hz, 1H), 5.12 (bt, *J* = 7.1 Hz, 1H), 4.99 (dd, *J* = 10.7, 1.3 Hz, 1H), 4.87 (bs, 1H), 4.77 (t, *J* = 1.6 Hz, 3H), 2.05–1.87 (m, 4H), 1.66 (s, 3H), 1.63–1.47 (m, 4H), 1.55 (s, 3H), 1.21 (s, 3H). ^13^C NMR (100 MHz, CDCl_3_): δ 147.5 (2C), 145.0 (2CH), 135.3 (2C), 124.7 (CH), 124.7 (CH), 111.7 (2CH_2_), 111.0 (2CH_2_), 75.7 (CH), 75.7 (CH), 73.5 (2C), 42.0 (2CH_2_), 42.0 (2CH_2_), 35.7 (2CH_2_), 33.1 (2CH_2_), 27.9 (CH_3_), 27.9 (CH_3_), 22.7 (2CH_2_), 17.6 (2CH_3_), 16.0 (2CH_3_).

#### 3.3.15. Oxidation of Compound **26**

To a solution of **26** (32 mg, 0.13 mmol) in dry DCM (1 mL), Dess-Martin periodinane (114 mg, 0.27 mmol) was added under an argon atmosphere at r t. This mixture was stirred for 1 h and 30 min. After completing the reaction, 20 mL of MTBE was added, and the mixture was washed with a saturated solution of Na_2_S_2_O_3_ (3 × 10 mL), water (3 × 10 mL), and brine (3 × 10 mL). Finally, the mixture was dried over Na_2_SO_4_ and evaporated under reduced pressure. Compound **28** (24 mg, 75% yield) was obtained by column chromatography using as eluent H:MTBE (4:1).

Compound **28**. [α]_D_ = −14.3 (c = 1, DCM). ^1^H NMR (400 MHz, CDCl_3_): δ 5.95 (s, 1H), 5.90 (dd, *J* = 17.3, 10.7 Hz, 1H), 5.76 (bs, *J* = 1.6, 0.8 Hz, 1H), 5.21 (dd, *J* = 17.4, 1.3 Hz, 1H), 5.14 (tq, *J* = 7.2, 1.3 Hz, 1H), 2.80–2.69 (m, 2H), 2.30–2.22 (m, 2H), 2.11–1.94 (m, 2H), 1.86 (t, *J* = 1.2 Hz, 3H), 1.61 (d, *J* = 1.3 Hz, 3H), 1.55 (dt, *J* = 10.4, 6.5 Hz, 2H), 1.27 (s, 3H). ^13^C NMR (100 MHz, CDCl_3_): δ 201.9 (C), 145.0 (CH), 144.5 (C), 134.4 (C), 124.8 (CH), 124.4 (CH_2_), 111.7 (CH_2_), 73.4 (C), 42.0 (CH_2_), 36.2 (CH_2_), 27.9 (CH_3_), 22.7 (CH_2_), 17.7 (CH_3_), 16.1. HRMS TOF (ES^+^) *m/z* calculated for C_15_H_25_O_2_ [M+H]^+^ 237.1855, found 237.1857.

#### 3.3.16. Oxidation of Compound **3**

To a solution of **3** (200 mg, 0.85 mmol) in DMF (6 mL), PDC (943 mg, 2.5 mmol) was added under an argon atmosphere at room temperature. This mixture was stirred for 53 h. After completing the reaction, 20 mL of water was added, and the reaction product was extracted using MTBE (2 × 50 mL) and EtOAc (1 × 50 mL). Finally, the organic phase was dried over Na_2_SO_4_ and evaporated under reduced pressure. Compound **29** (128 mg, 64% yield) was obtained by column chromatography using as eluent H:MTBE (7:3).

#### 3.3.17. Reaction of Compound **29** with I_2_ and DMSO

To a solution of **29** (127 mg, 0.532 mmol) in hexane (5 mL), DMSO (0.19 mL), and I_2_ (28 mg, 0.11 mmol) were added under an argon atmosphere at 90 °C. This mixture was stirred for 1 h. After completing the reaction, 120 mL of MTBE was added, and the mixture was washed with a saturated solution of Na_2_S_2_O_3_ (2 × 50 mL), distilled water (3 × 50 mL), and brine (3 × 50 mL). Finally, the organic phase was dried over Na_2_SO4 and evaporated under reduced pressure. Compounds **30** (31 mg, 25% yield), **31** (12 mg, 10% yield), and **32** (10 mg, 9% yield) were obtained by column chromatography using as eluent H:MTBE (10:1).

Compound **30**. [α]_D_ = −14.3 (*c* = 1, DCM). ^1^H NMR (500 MHz, CDCl_3_): δ 5.95 (bs, 1H), 5.92 (bs, 1H), 5.93 (dd, *J* = 17.4, 10.7 Hz, 1H), 5.24 (dd, *J* = 17.4, 1.5 Hz, 1H), 5.08 (dd, *J* = 10.7, 1.5 Hz, 1H), 2.65–2.53 (m, 2H), 1.94 (s, 3H), 1.91 (s, 3H), 1.89–1.79 (m, 2H), 1.85 (s, 3H), 1.31 (s, 3H). ^13^C NMR (100 MHz, CDCl_3_): δ 150.9 (C), 148.8 (C), 144.6 (CH), 133.4 (C), 115.1 (C), 114.4 (CH), 112.0 (CH_2_), 110.6 (CH), 73.0 (C), 40.3 (CH_2_), 28.0 (CH_3_), 26.9 (CH_3_), 20.6 (CH_2_), 20.1 (CH_3_), 9.8 (CH_3_). HRMS TOF (ES^+^) *m/z* calculated for C_15_H_23_O_2_ [M+H]^+^ 235.1698, found 235.1691.

Compound **31**. [α]_D_ = −34.3 (*c* = 1, DCM). 1H NMR (500 MHz, CDCl_3_): δ 6.38 (t, *J* = 1.4 Hz, 1H), 6.12 (*p*, *J* = 1.3 Hz, 1H), 5.88 (dd, *J* = 17.3, 10.7 Hz, 1H), 5.21 (dd, *J* = 17.3, 1.5 Hz, 1H), 5.03 (dd, *J* = 10.7, 1.5 Hz), 4.40 (dd, *J* = 7.7, 5.9 Hz, 1H), 2.22–2.14 (m, 1H), 2.17 (d, *J* = 1.3 Hz, 3H), 2.09 (d, *J* = 1.3 Hz, 3H), 1.91–1.86 (m, 1H), 1.89 (d, *J* = 1.3 Hz, 3H), 1.78–1.71 (m, 2H), 1.39 (s, 3H). ^13^C NMR (100 MHz, CDCl_3_): δ 192.0 (C), 156.4 (C), 154.6 (C), 143.4 (CH), 126.4 (CH), 123.1 (CH), 111.8 (CH_2_), 84.0 (C), 82.4 (CH), 36.6 (CH_2_), 31.0 (CH_2_), 27.8 (CH_3_), 26.7 (CH_3_), 20.6 (CH_3_), 15.9 (CH_3_). HRMS TOF (ES^+^) *m/z* calculated for C_15_H_23_O_2_ [M+H]^+^ 235.1695, found 235.1698.

Compound **32.** [α]_D_ = +17.0 (*c* = 1, DCM). ^1^H NMR (500 MHz, CDCl_3_): δ 6.42 (*p*, *J* = 1.3 Hz, 1H), 6.11 (*p*, *J* = 1.3 Hz, 1H), 5.99 (dd, *J* = 17.3, 10.8 Hz, 1H), 5.24 (dd, *J* = 17.3, 1.3 Hz, 1H), 5.03 (dd, *J* = 10.8, 1.3 Hz), 4.44 (bt, *J* = 7.8 Hz, 1H), 2.24–2.13 (m, 1H), 2.17 (d, *J* = 1.3 Hz, 3H), 2.09 (d, *J* = 1.3 Hz, 3H), 1.99–1.93 (m, 1H), 1.90 (s, 3H), 1.85–1.69 (m, 2H), 1.36 (s, 3H). ^13^C NMR (100 MHz, CDCl_3_): δ 192.0 (C), 155.9 (C), 154.6 (C), 144.4 (CH), 126.4 (CH), 123.2 (CH), 111.9 (CH_2_), 83.6 (C), 82.6 (CH), 37.5 (CH_2_), 31.7 (CH_2_), 27.8 (CH_3_), 26.3 (CH_3_), 20.6 (CH_3_), 15.8 (CH_3_). HRMS TOF (ES^+^) *m/z* calculated for C_15_H_23_O_2_ [M+H]^+^ 235.1694, found 235.1698.

#### 3.3.18. Acetylation of Compound **3**

To a solution of **3** (500 mg, 2.08 mmol) in pyridine (3 mL), Ac_2_O (2.5 mL) was added under an argon atmosphere at room temperature. This mixture was stirred for 5 h. After completing the reaction, 100 mL of ice was added, and then 40 mL of MTBE. When the ice melted, the organic phase was washed with HCl 2N (4 × 20 mL), then a saturated solution of NaHCO_3_ (3 × 20 mL), followed by distilled water (2 × 20 mL) and brine (3 × 15 mL). Finally, the mixture was dried over Na_2_SO_4_ and evaporated under reduced pressure. Compound **3a** (523 mg, 89% yield) was obtained by column chromatography using as eluent H:MTBE (2:1).

#### 3.3.19. Epoxidation of Compound **3a**

To a solution of **3a** (280 mg, 1 mmol) in dichloromethane (17 mL), *m-*chloroperbenzoic acid (252 mg, 1.45 mmol) was added under an argon atmosphere at 0 °C. This mixture was stirred for 1 h. After completing the reaction, 50 mL of MTBE was added to the mixture, and it was washed with a saturated solution of Na_2_S_2_O_3_ (3 × 15 mL), then a saturated solution of NaHCO_3_ (3 × 15 mL), and finally brine. The mixture was dried over Na_2_SO_4_ and evaporated under reduced pressure. Compound **33** (264 mg, 94% yield) was obtained by column chromatography using as eluent H:MTBE (2:1).

**Compound 33.** ^1^H NMR (400 MHz, Acetone-d6) δ 5.96 (dd, *J* = 17.2, 10.8 Hz, 1H), 5.71–5.65 (m, 1H), 5.24 (dd, *J* = 17.3, 1.5 Hz, 1H), 5.15 (t, 1H), 5.00 (dd, *J* = 10.7, 1.7 Hz, 1H), 2.70 (t, 1H), 2.11 (d, 2H), 2.08–2.06 (m, 2H), 1.99 (s, 3H), 1.76 (s, 3H), 1.74 (s, 3H), 1.71 (s, 3H), 1.60–1.56 (m, 2H), 1.26 (s, 3H). ^13^C NMR (100 MHz, Acetone-d6) δ 169.3 (C), 145.8 (CH), 136.0 (C), 124.1 (CH), 110.7 (CH_2_), 71.7 (C), 68.3 (CH), 63.1 (CH), 57.8 (C), 44.5 (CH_2_), 38.6 (CH_2_), 29.0 (CH_3_), 24.8 (CH_3_), 23.4 (CH_2_), 20.4 (CH_3_), 17.5 (CH_3_), 16.1 (CH_3_).

### 3.4. Antifeedant Bioassay

*Spodoptera littoralis* Boisduval, 1833 (Lepidoptera, Noctuidae) colonies were reared on an artificial diet [50], while *Myzus persicae* Sulzer, 1776 (Homoptera, Aphididae), and *Rhopalosiphum padi* L., 1758 (Hemiptera, Aphididae) colonies were maintained on bell pepper (*Capsicum annuum*) and barley (*Hordeum vulgare*) plants, respectively. The plants were grown from seeds in pots with commercial substrate and regularly infected with aphid feeding (bell pepper plants were infected at a 4-leaf stage, and barley plants when they reached approximately a length of 10 cm). Both the insect colonies and their host plants were maintained in a growth chamber at 22 ± 1 °C, >70% relative humidity, with a 16:8 h light photoperiod.

Bioassays were conducted with 1.0 cm^2^ leaf disks/fragments of *C. annuum* (*M. persicae, S. littoralis*) or *H. vulgare* (*R. padi*) as described previously [51]. The tests (10 μL of the solution in EtOH) were applied at initial doses of 10 or 5 mg/mL (extract or compound) to the upper surface of the leaf fragments. Two sixth-instar larvae (>24 h after molting) of *S. littoralis* were placed in 6 Petri dishes (9 cm in diameter) with 2 leaf disks (treatment disk with the test solution and control disk with solvent) and allowed to feed at room temperature until 75% larval consumption of the paired control or treatment disks. The leaf disk surface consumption was measured using ImageJ (Version 1.51, 23 April 2018) (http://imagej.nih.gov/ij/, accessed on 18 March 2025) [52]. In the case of aphids, twenty (2 × 2 cm) ventilated plastic boxes containing 10 apterous aphid adults (24–48 h old) were used. The aphids were allowed to feed in a growth chamber under the described environmental conditions for 24 h. Settling was quantified by counting the number of aphids settled on each leaf fragment. All the experiments were repeated twice (SE < 10%).

The feeding or settling inhibition (%FI or %SI) was calculated using the formula [1 − (T/C) × 100], where T is the treated leaf fragment and C is the control, respectively. The effects (%SI/%FI) were analyzed by the nonparametric Wilcoxon Signed-Rank Test. Compounds with an effect ≥70% were tested in dose–response experiments (3–5 serial dilutions) to calculate their EC_50_ (the effective dose causing a 50% settling/feeding reduction) with linear regression models (%FI/SI on Log-dose). The positive control was thymol (Sigma Aldrich, St. Louis, MO, USA).

### 3.5. Nematicidal Bioassay

The *Meloidogyne javanica* Treub, 1885 (Tylenchida, Heteroderidae) population was maintained on tomato plants (*Solanum lycopersicum* var. Marmande) cultivated in pot cultures and kept in environmentally controlled growth chambers (at 25 ± 1 °C, >70% relative humidity). Egg masses of *M. javanica* were handpicked from the infected tomato roots two months after seedling inoculation. Second-stage juveniles (J2) were obtained by incubating egg masses in a water suspension at 25 °C for 24 h. The tests were carried out in 96-well plates (BD Falcon, San Jose, CA, USA), and the extract and compounds were dissolved in distilled water containing 5% of a DMSO-Tween solution (0.2% Tween 20 in DMSO) according to Andrés et al. [53]. The initial concentrations tested were 0.5 mg/mL. For each test, four replicates were carried out with 100 J2 wells. Water containing 5% of a DMSO-Tween solution (0.2% Tween 20 in DMSO) was used for the negative control. The mortality rates after 72 h of incubation are presented as a percentage of dead J2 corrected according to Scheider-Orelli’s formula. The result values were analyzed by ANOVA, and the means were compared by LSD at *p* < 0.05. Serial dilutions were used to calculate the effective lethal doses (LD_50_) of the active compound by Probit Analysis (STATGRAPHICS Centurion XVI, version 16.1.03). Thymol (Sigma Aldrich, St. Louis, MO, USA) was used as a positive control.

### 3.6. Phytotoxic Bioassay

The phytotoxic test was conducted with *Lolium perenne* L., 1753 (Poales, Poaceae) and *Lactuca sativa* L., 1753 (Asterales, Asteraceae) seeds placed in 12-well microplates (40 seeds for the test), as described in [54]. The extract or compounds dissolved in EtOH (negative control) were tested at concentrations of 0.4 or 0.2 mg/mL (final concentration in the well) and diluted serially if needed. Juglone (Sigma, St. Louis, MO, USA) was used as a positive control (0.1 mg/mL), resulting in 100% germination inhibition. Briefly, the test solution (20 μL) and 300 μL of H_2_O were added to a 2.5 cm diameter filter paper in each well plate. The seeds (10/5 of *L. sativa*/*L. perenne* soaked in distilled water for 8 h) were placed in every well, and the parafilm-sealed plates were incubated in a plant growth chamber (25 °C, 70% RH, 16:8 L:D). Germination was monitored for 7 days, and leaf length (for *L. perenne*) and root length (for both species) were measured at the end of the experiment on 25 randomly selected digitalized seedlings with the ImageJ application (http://rsb.info.nih.gov/ij/, accessed on 29 May 2025) [55]. A nonparametric Kruskal—Wallis ANOVA analysis of variance was performed using the STATGRAPHICS statistical analysis software (Centurion XVI, version 16.1.03) on root/leaf length data, and the means were compared by the Mann–Whitney U test at *p* < 0.05. The positive control used is juglone (Sigma, St. Louis, MO, USA), with 100% effectivity in all tests.

### 3.7. Ixodicidal Activity

*Hyalomma lusitanicum* engorged females were collected from red deer in Ciudad Real (Central Spain) and maintained under laboratory conditions [22–24°C and 80% relative humidity (RH)] until oviposition and egg hatching.

Tick bioassays were performed according to Valcarcel et al. et al. [37]. Briefly, 50 µL of the test solution was added to 25 mg of powdered cellulose at different concentrations (initial concentration of 20 µg/mg for pure compounds), and the solvent was evaporated. The ticks and cellulose were then placed in laboratory glass tubes and carefully mixed by rotating the glass several times to ensure full tick–cellulose contact. After mixing, the tubes were kept under the same conditions as above for 24 h. For each test, three replicates were carried out with 20 active larvae older than 6 weeks each. To validate the tests, three replicates of negative (cellulose, 25 mg) and positive (thymol, 20 µg/mg) controls were also used. Ticks were considered dead when they could not move from one place to another. Dead ticks were counted after 24 h of contact with the treated cellulose under the laboratory conditions described, using a binocular magnifying glass. The ixocidal activity data are presented as percent mortality corrected according to Schneider–Orelli’s formula [56]. Effective lethal doses (LC_50_ and LC_90_) were calculated by Probit analysis (1:2 serial dilutions to cover a range of activities between 100 and <50% mortality with a minimum of three doses) (STATGRAPHICS Centurion XVI, version 16.1.02).

## 4. Conclusions

This study confirms that *Dittrichia viscosa* is a valuable source of natural compounds for sustainable plant protection. Three major metabolites—ilicic acid, nerolidol, and 9-hydroxynerolidol—were isolated and transformed into 29 derivatives using methods such as aromatization, epoxidation, and cyclization. Their biological evaluation revealed selective and target-specific bioactivity: Compound **11**, a lactone derivative of ilicic acid, showed broad-spectrum activity against pests like *Rhopalosiphum padi* and *Meloidogyne javanica*, as well as ryegrass and ticks. However, it was ineffective against *Spodoptera littoralis* and *Myzus persicae*. This cross-target effectiveness makes it particularly promising as a versatile biopesticide lead. Compound **29** (9-oxonerolidol), derived in a single step from 9-hydroxynerolidol, displayed potent acaricidal activity against *H. lusitanicum*, achieving an LD_50_ six times more effective than the benchmark compound nootkatone. Several compounds (e.g., **3, 4, 11, 17**, and **20**–**21**) exhibited strong herbicidal activity on ryegrass without compromising lettuce growth, with some even stimulating root development, highlighting their selectivity and application potential. Remarkably, both compounds **11** and **29** can be synthesized via single-step transformations from natural precursors, making them cost-effective and scalable candidates for further development. These findings highlight the potential of these compounds as cost-effective biocontrol agents for integrated pest management.

## Figures and Tables

**Table 1:** Generation of molecular diversity from ester **4.**

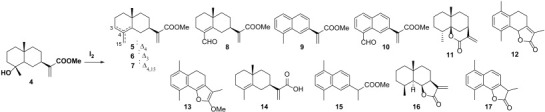				
Entry	I_2_ (Equiv)	Solvent	Time (h)	Compound (Yield)
entry 1	1	benzene	5	**5** (85%)
entry 2	1	toluene	10	**5** (48%), **6** (9%), **7** (18%)
entry 3	0.25	toluene:DMSO (8:1)	18	**5** (37%), **6** (15%), **7** (8%), **8** (4%), **9** (4%), **10** (6%)
entry 4	2	toluene:MeOH (9:1)	6.5	**5** (45%), **11** (6%), **12** (19%), **13** (9%)
entry 5	2	toluene:MeOH (9:1)	16	**5** (40%), **11** (9%), **12** (19%), **13** (17%).
entry 6	2	toluene:MeOH (9:1)	38	**5** (24%), **11** (10%), **14** (22%), **15**(10%), **16** (7%), **17** (12%)

**Table 2:** Generation of molecular diversity from ester **5**.

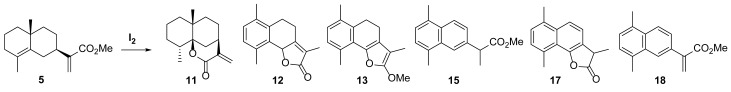				
Entry	I_2_ (Equiv)	Solvent	Time (h)	Compound (Yield)
entry 1	2	toluene:MeOH (9:1)	13	**11** (9%), **12** (25%), **15** (14%), **17** (10%)
entry 2	2	toluene:MeOH (9:1)	32	**11** (6%), **12** (8%), **13** (7%), **18** (24%)

**Table 3:** Generation of molecular diversity from the epoxides **20** and **21.**

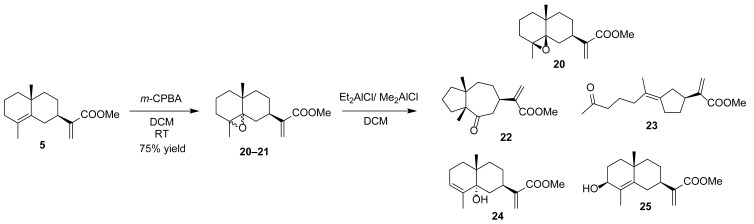				
**Entry**	**Reactant**	**Temperature**	**Time (h)**	**Compound (Yield)**
entry 1	Et_2_AlCl (2 equiv)	−60 °C	6	**20** (38%), **22** (39%), **23** (4%)
entry 2	Me_2_AlCl (3 equiv)	−40 °C	6	**20** (2%), **22** (41%), **23** (2%), **24** (24%), **25** (12%)

**Table 4:** Insect antifeedant effects of nerolidol (**2**), ilicic acid (**1**), and derivatives **3**, **3a**, **26**–**29**, **33**, **4**, **5**, **11**, **17**, and **20**–**21** against *Spodoptera littoralis*, *Myzus persicae*, and *Rhopalosiohum padi* (data are expressed as average ± standard error).

Compound	μg/cm^2^	*S. littoralis*	*M. persicae*	*R. padi*
		%FI ^b^ (n = 6–10)	%SI ^b^ (n = 20)	
Nerolidol (**2**)	50	25.91 ± 16.44	60.94 ± 10.32 *	54.02 ± 8.34 *
	EC_50_ ^a^	>50	≅50	≥50
**3**	50	31.10 ± 9.70	29.21 ± 8.55	69.30 ± 6.20 *
	EC_50_	>50	>50	25–50
**3a**	50	48.70 ± 15.02	56.80 ± 7.38 *	88.32 ± 4.12 *
	EC_50_	>50	≥50	8.34 (6.3–11.1)
**26**	50	24.63 ± 14.52	83.55 ± 5.97 *	67.08 ± 6.85 *
	EC_50_	>50	18.91 (12.71–28.12)	≅50
**27**	50	29.14 ± 15.85	100 *	80.16 ± 5.36 *
	EC_50_	>50	9.97 (5.85–16.98)	19.97 (15.66–25.47)
**28**	50	28.78 ± 15.35	70.87 ± 5.22 *	78.49 ± 5.29 *
	EC_50_	>50	15.11 (8.05–28.37)	11.95 (6.23–22.92)
**29**	50	50.19 ± 8.67 *	66.10 ± 7.36 *	76.37 ± 7.76 *
	EC_50_	≥50	≅50	21.3 (16.2–25.3)
**33**	50	34.43 ± 14.66	23.70 ± 7.14	44.24 ± 6.88
	EC_50_	>50	>50	>50
Ilicic acid (**1**)	50	51.77 ± 9.50 *	39.05 ± 8.16	27.34 ± 7.83
	EC_50_	≥50	>50	>50
**4**	50	40.45 ± 12.96	51.01 ± 7.56 *	96.05 ± 2.15 *
	EC_50_	>50	≥50	15.90 (13.04–19.26)
**5**	50	nt	61.62 ± 8.35 *	92.32 ± 4.03 *
	EC_50_		≅50	22.6 (20.3–25.1)
**11**	50	76.09 ± 11.94 *	54.34 ± 8.69 *	89.72 ± 5.07 *
	EC_50_	25.28 (17.96–35.57)	≥50	11.11 (8.81–14.01)
**17**	50	nt	28.90 ± 8.27	34.18 ± 8.04
	EC_50_		>50	>50
**20**–**21**	50	41.70 ± 8.91	57.65 ± 8.49 *	56.53 ± 8.03 *
	EC_50_	>50	≥50	≥50
Thymol ^c^	50	52.4 ± 10.1 *	81.8 ± 7.7 *	92.1 ± 2.6 *
	EC_50_	≥50	7.6 (4.1–8.7)	18.6 (4.1–23.3.5)

^a^ EC_50_: Effective dose to give 50% effect. ^b^ %FI/SI = [1 − (consumption/settling on treated disk/consumption/settling on control disk)] × 100. ^c^ From Valcarcel et al. [37]. * Values with asterisk indicate significant differences between treatment and control according to Wilcoxon paired rank test (*p* < 0.05).

**Table 5:** In vitro nematicidal effects of compounds nerolidol (**2**), ilicic acid (**1**), and derivatives **3**, **3a**, **26**–**29**, **33**, **4**, **5**, **11**, and **20**–**21** against *Meloidogyne javanica*.

Compound	Mortality (%)	Lethal Dose (ug/uL) ^a^	
	(1 ug/uL)	LD_50_	LD_90_
Nerolidol (**2**)	10.97 ± 2.01		
**3**	3.63 ± 0.73		
**3a**	0.13 ± 0.71		
**26**	17.41± 3.50		
**27**	14.47 ± 2.87		
**28**	100 ± 0	MLC ^b^ = 0.5	
**29**	2.89 ± 0.97		
**33**	10.97 ± 1.35		
Ilicic acid (**1**)	19.74 ±1.80		
**4**	61.11 ± 5.60		
**5**	1.49 ± 1.21		
**11**	93.24 ± 3.15	0.31 (0.29–0.33)	0.59 (0.55–0.65)
**20**–**21**	5.98 ± 2.57		
Thymol ^c^	100 ± 0	0.14 (0.13–0.14)	0.22 (0.2 1–0.25)

^a^ Lethal dose to give 50 and 90% mortality (LD_50_,→LD_90_); ^b^ Minimum lethal concentration (MLC); ^c^ From González-López et al. [38].

**Table 6:** Acaricidal activity of the compounds nerolidol (**2**), ilicic acid **(1**), and derivatives **3**, **3a**, **29**, **33**, **4**, **5**, **11**, and **17** against *Hyalomma lusitanicum* larvae.

Compound	% Mortality ^a^ (20 μg/μL)	Effective Concentration (μg/mg) ^b^	
		LD_50_	LD_90_
Nerolidol (**2**)	10. 97 ± 2.01	>20	>20
**3**	100 ± 0	2.30 (2.07–2.58)	3.44 (3.05–4.14)
**3a**	100 ± 0	2.58 (2.29–2.92)	5.25 (4.62–6.21)
**29**	100 ±0	0.62 (0.55–0.70)	1.09 (0.97–1.26)
**33**	100 ± 0	Nc	MLD ^c^ = 5
Ilicic acid (**1**)	8 47 ± 4.61	>20	>20
4	25.8 ± 1.43	>20	>20
**5**	98.4 ± 0.6	1.71 (1.52–1.87)	2.78 (2.54–3.15)
**11**	100 ± 0	2.61 (2.39–2.88)	3.69 (3.35–4.18)
**17**	90.3 ± 3.8	2.36 (1.29–3.18)	8.26 (6.96–10.49)
Thymol ^d^	100 ± 0	2.94 (2.08–3.54)	6.16 (5.30–7.84)

^a^ Mortality data corrected according to Schenider Orelli’s formula. ^b^ Doses needed to give 50 and 90% mortality (95% Confidence Limits). ^c^ Minimum Lethal Dose to give 100% mortality. ^d^ From Valcarcel et al. [37]. Nc not calculated.

## Data Availability

The original contributions presented in this study are included in the article/Supplementary Materials. Further inquiries can be directed to the corresponding authors.

## Supplementary Materials

The following supporting information can be downloaded at: https://www.mdpi.com/article/10.3390/molecules30193950/s1. Table S1: Phytotoxicity (%) of the compounds tested ilicic acid (**1**), (nerolidol (**2**), and derivatives **3**, **3a**, **26**–**29**, **33**, **4**, **5**, **11**, **17**, and **20**–**21** against *Lolium perenne* and *Lactuca sativa*. Data is expressed as average ± standard error (n = 5 for germination and 25 for leaf and root growth measurements). Figures S1–S53: NMR spectra.
