# Supplementary material for: Major Components of Dittrichia viscosa (Asteraceae) as a Source of New Pesticides
Source: Molecules. 2025 Oct 1;30(19):3950. doi: 10.3390/molecules30193950 (PMC12526312; doi:10.3390/molecules30193950)
Supplement: Supplementary file 1 [file molecules-30-03950-s001.zip › molecules-3847243-supplementary.pdf]

Major Components of *Dittrichia viscosa* [Asteraceae] as Source of new Pesticides

María José Segura-Navarro [1], José Francisco Quílez del Moral [1]*, María Fe Andrés [2], Félix Valcárcel [3], Azucena González-Coloma [2]*, Diego O. Molina Inzunza [1], Alejandro Fernández Barrero [1]*

[1] Department of Organic Chemistry, Institute of Biotechnology, University of Granada, 18071 Granada, Spain.

[2] Institute of Agricultural Sciences, CSIC, 28006, Madrid, Spain.

[3] Instituto Nacional de Investigación y Tecnologías Agrarias y Alimentarias, INIA-CSIC, 28040 Madrid, Spain.

* Corresponding authors:

E-mail address: jfquilez@ugr.es; afbarre@ugr.es; azu@ica.csic.es.

List of contents

Table S1	3
NMR spectra	4-33

Table S1. Phytotoxicity (%) of the compounds tested ilicic acid (**1**), (nerolidol (**2**) and derivatives **3**, **3a**, **26-29**, **33**, **4**, **5**, **11**, **17** and **20-21**) against *Lolium perenne* and *Lactuca sativa.* Data is expressed as average ± standard error (n = 5 for germination and 25 for leaf and root growth).measurements).

Compound	mg/ml	*Lolium perenne*			*Lactuca sativa*	
		Germination	Root	Leaf	Germination	Root
**Ilicic acid (1)**	0.1	102.6 ± 5.4	109.87± 57.3	118.26± 6.5	78.4± 5.5	108.7± 8.0
**4**	0.1	54.5 ± 6.8	0	0	95.0 ± 5.0	89.92 ± 6.3
	0.05	86.1 ± 9.5	83.2 ± 15.0	76.1 ± 14.1		
**5**	0.1	85.7± 5.4	105.1± 6.5	64.6± 57.3	97.4 ± 4.8	133.1 ± 5.7
**8**	0.1	12.1 ± 5.0	0	0	82.5 ± 7.5	78.95 ± 2.8
	0.05	83.3 ± 3.2	61.62 ± 9.1	60.08 ± 11.2		
**11**	0.1	12.1 ± 5.7	0	0	82.5± 57.5	78.95± 9.0
	0.05	83.3 ± 7.3	61.6 ± 6.5	60.1 ± 5.8		
**17**	0.1	17.6 ± 7.7	0	0	97.5 ± 2.5	186.3 ± 10.4
	0.05	103.2 ± 6.2	91.2 ± 10.5	103.7 ± 8.3		
**20-21**	0.1	94.6 ± 11.3	70.0 ± 7.9	27.3 ± 4.7	100	142.7 ± 7.44
	0.05	102.8 ± 7.2	98.1 ± 11.5	64.5 ± 7.2		
**Nerolidol (2)**	0.1	78.9 ± 6.7	77.1 ± 3.9	77.9 ± 6.1	100	124.4 ± 4.7
**26**	0.1	97.4 ± 7.8	92.9 ± 3.7	91.7 ± 5.5	97.5± 7.3	118.1 ± 7.7
**27**	0.1	100	85.6 ± 4.5	93.8 ± 6.1	100	125.2 ± 6.2
**3**	0.1	88.6 ± 9.4	59.6 ± 8.0	38.5 ± 6.9	97.3 ± 6.7	155.86 ± 6.5
	0.05	109.4 ± 13.8	101.29 ± 9.3	103.4 ± 8.3		
**3a**	0.1	100	73.0 ± 8.22	79.46 ± 8.9	91.9 ± 8.4	156.9 ± 6.9
**29**	0.1	82.9 ± 9.0	57.12 ± 7.8	53.12 ± 9.2	102.7 ± 6.2	136.8 ± 5.8
	0.05	121.9 ± 14.3	97.7 ± 9.2	94.70 ± 7.6		
**33**	0.1	102.9 ± 10.0	85.6 ± 9.7	81.02 ± 8.1	102.7 ± 6.2	112.33 ± 4.7

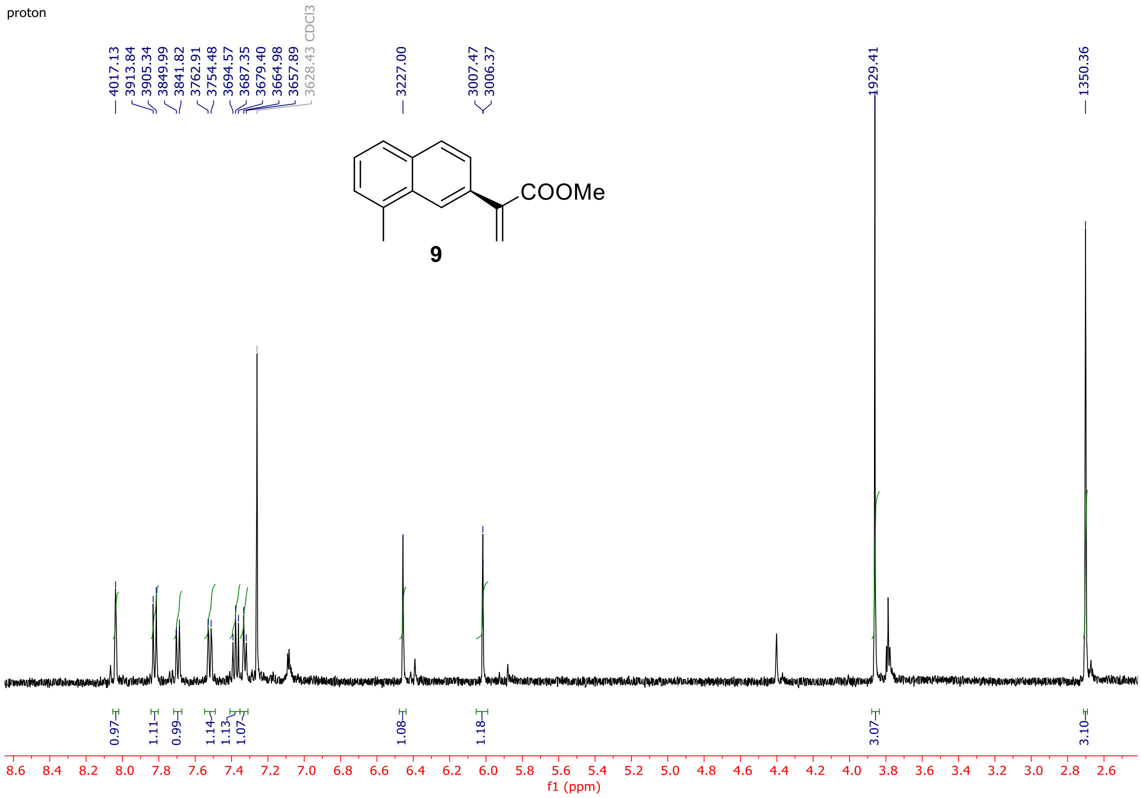

Figure S1. ^{1}H-NMR ($CDCl_3$, 400 MHz) spectrum of compound **9**

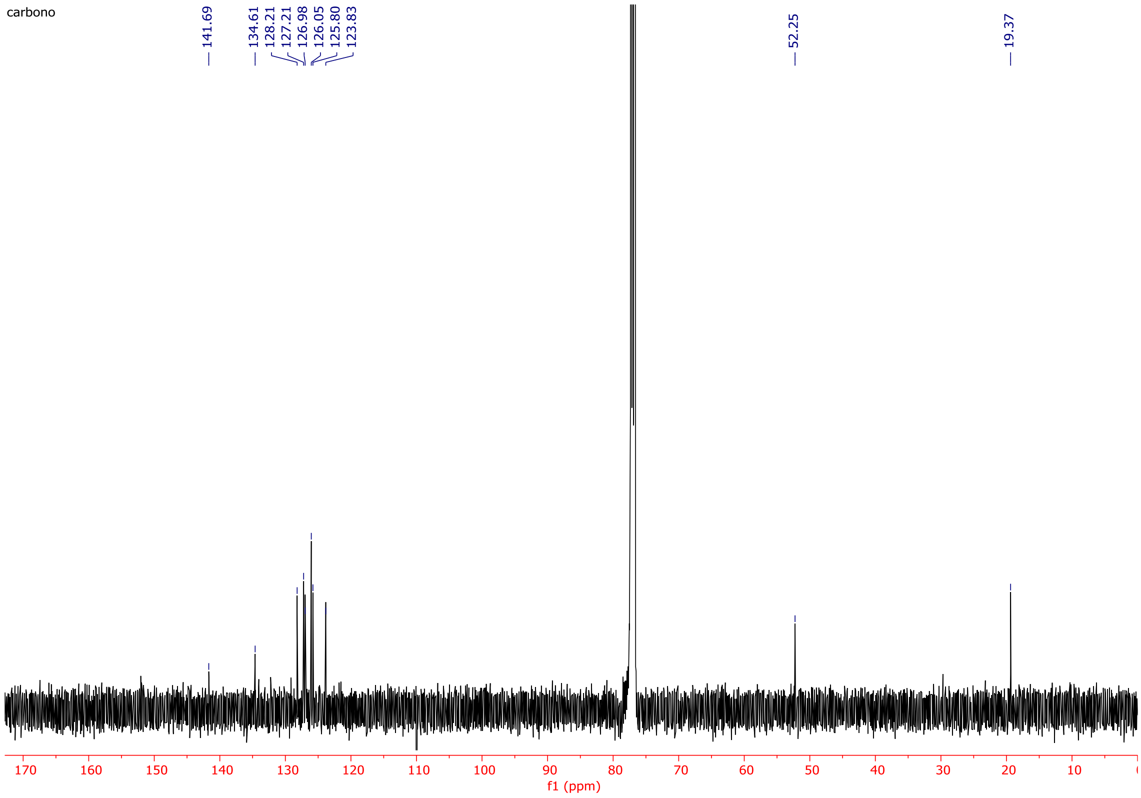

Figure S2. ^{13}C-NMR ($CDCl_3$, 100 MHz) spectrum of compound **9**

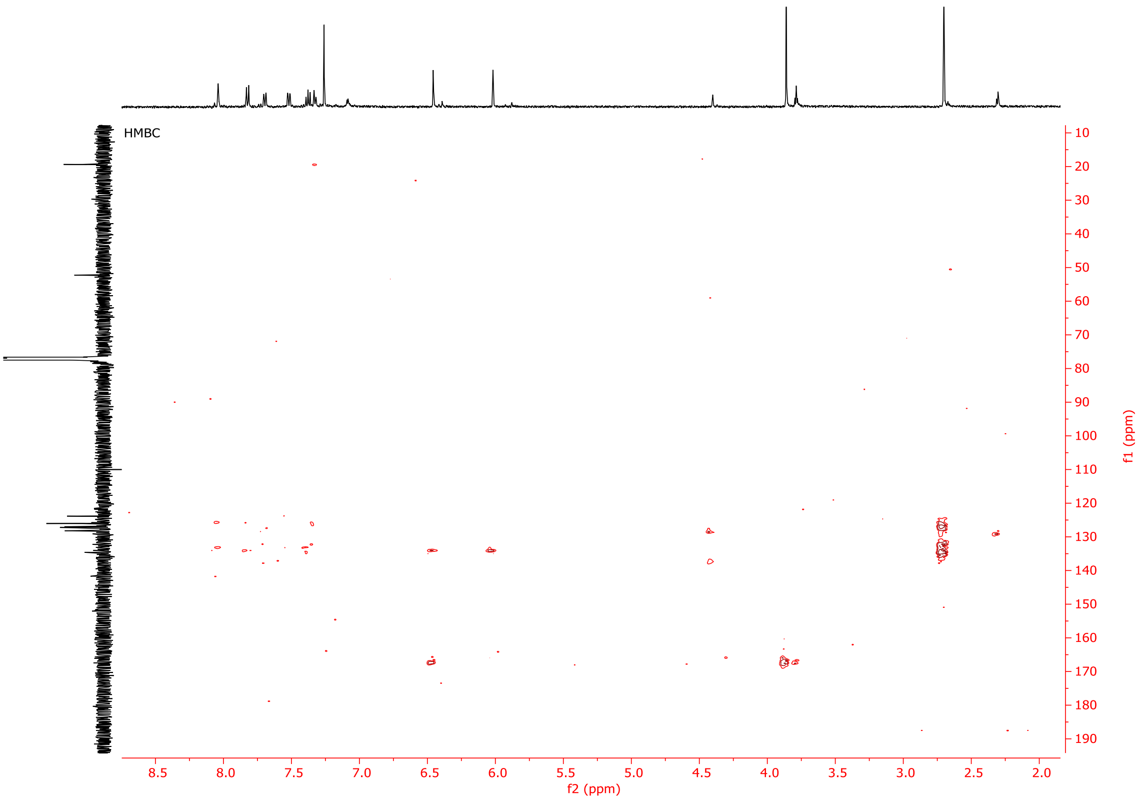

Figure S3. HMBC ($CDCl_3$, 400/100 MHz) of compound **9**

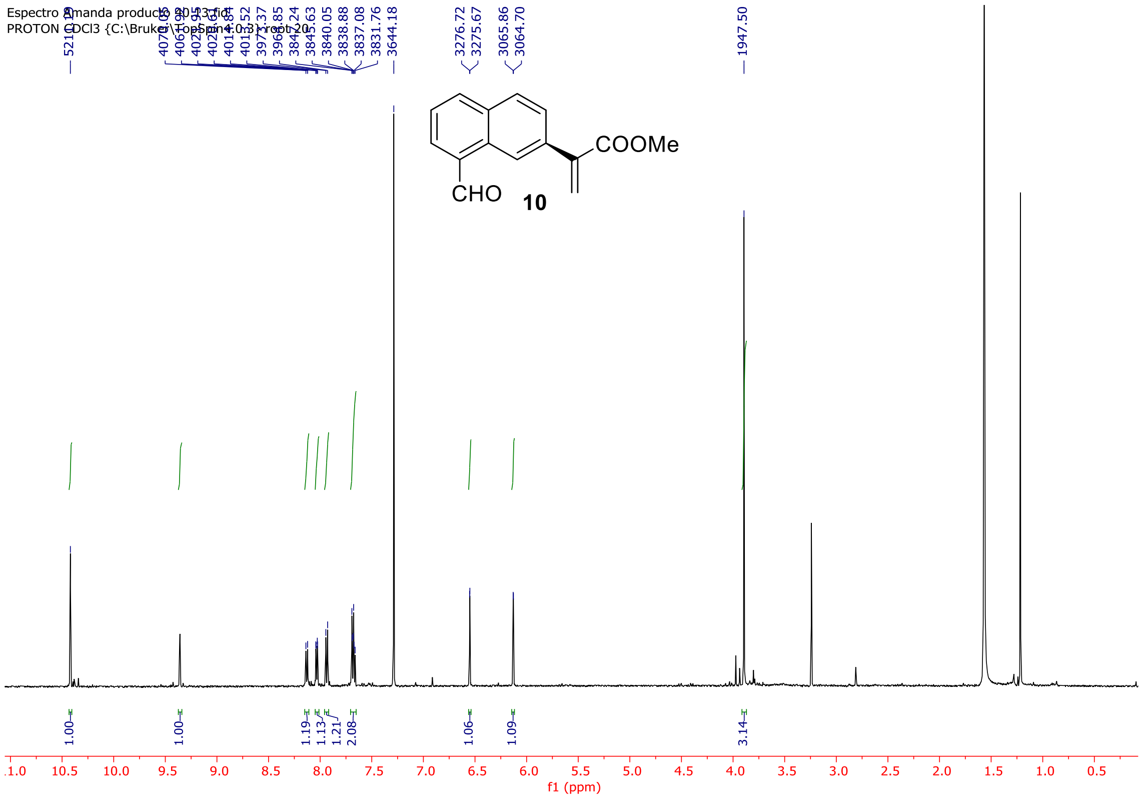

Figure S4. ^{1}H-NMR ($CDCl_3$, 400 MHz) spectrum of compound **10**

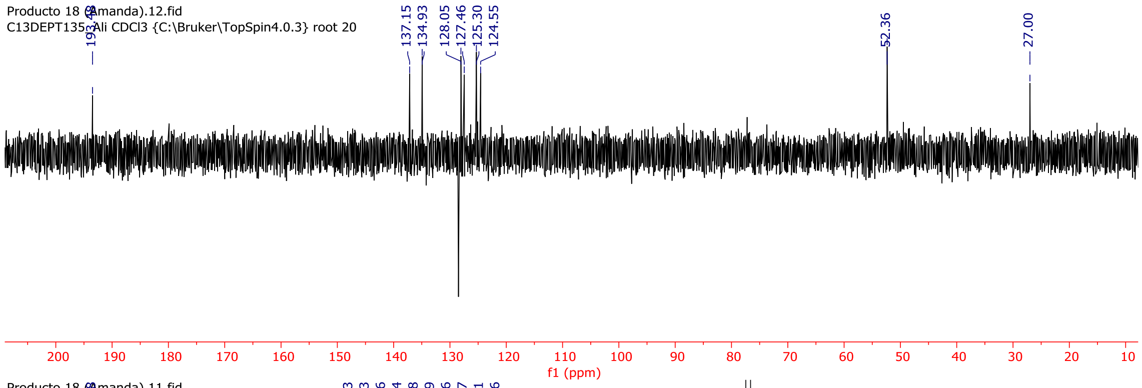

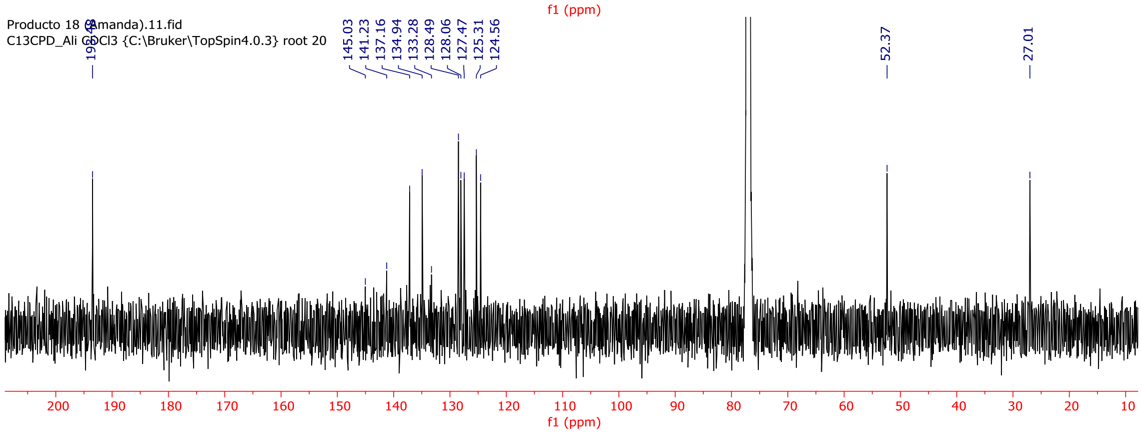

Figure S5. ^{13}C-NMR ($CDCl_3$, 100 MHz) spectrum of compound **10**

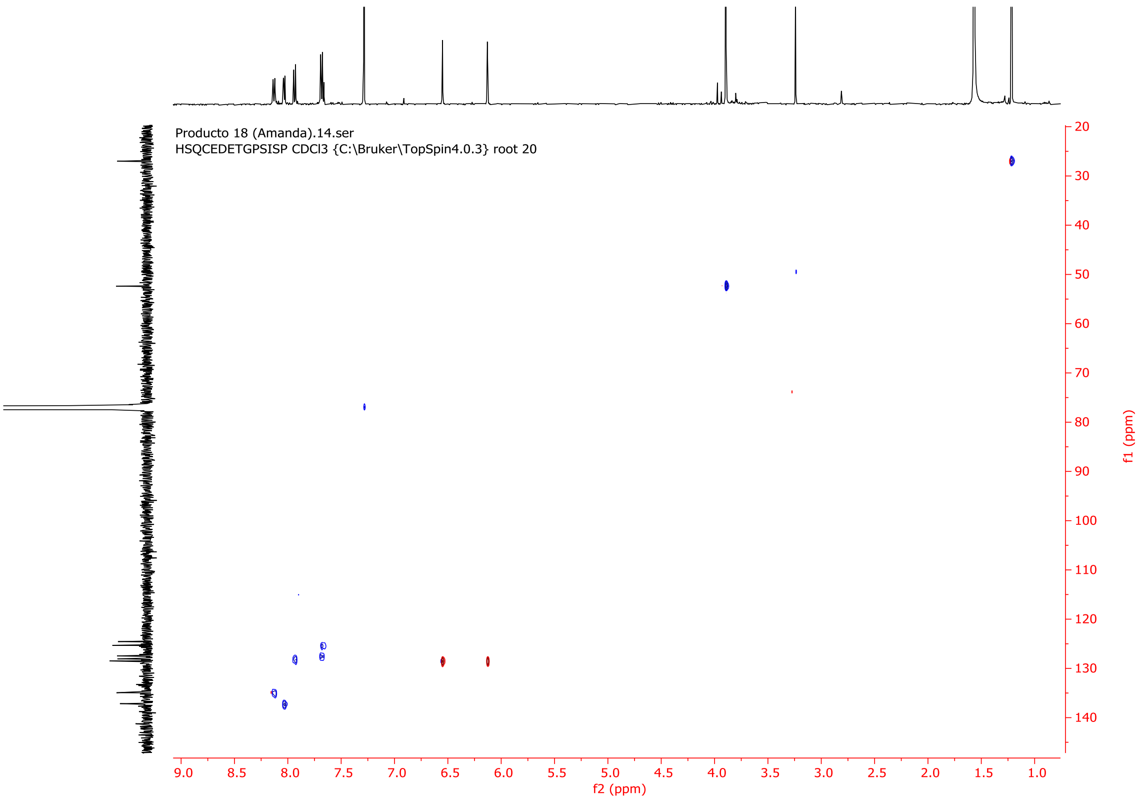

Figure S6. HSQC ($CDCl_3$, 400/100 MHz) of compound **10**

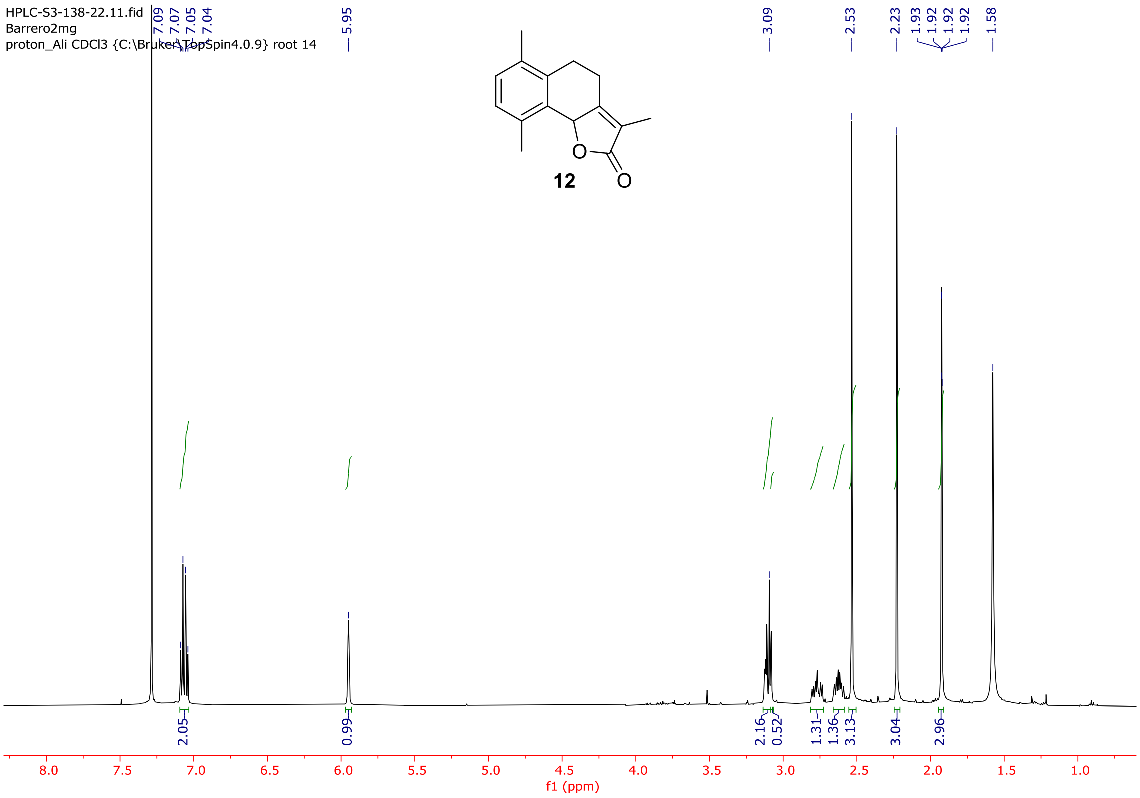

Figure S7. ^{1}H-NMR ($CDCl_3$, 400 MHz) spectrum of compound **12**

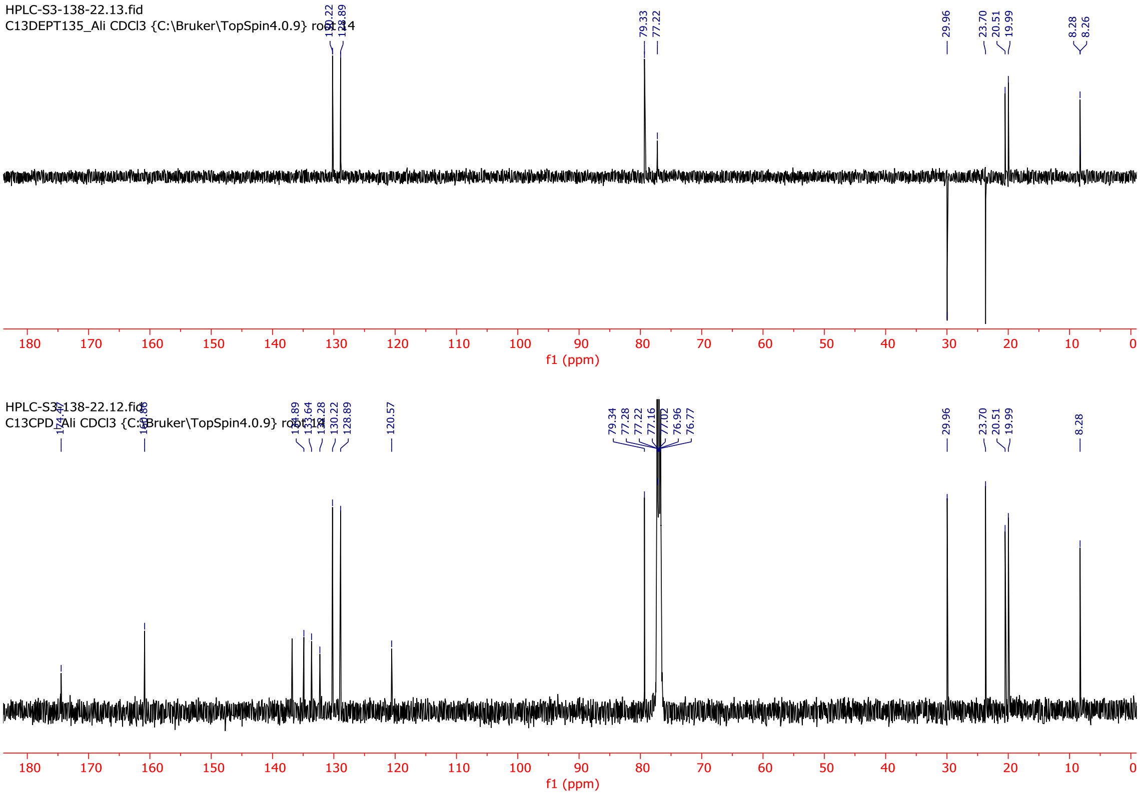

Figure S8. ^{13}C-NMR ($CDCl_3$, 100 MHz) spectrum of compound **12**

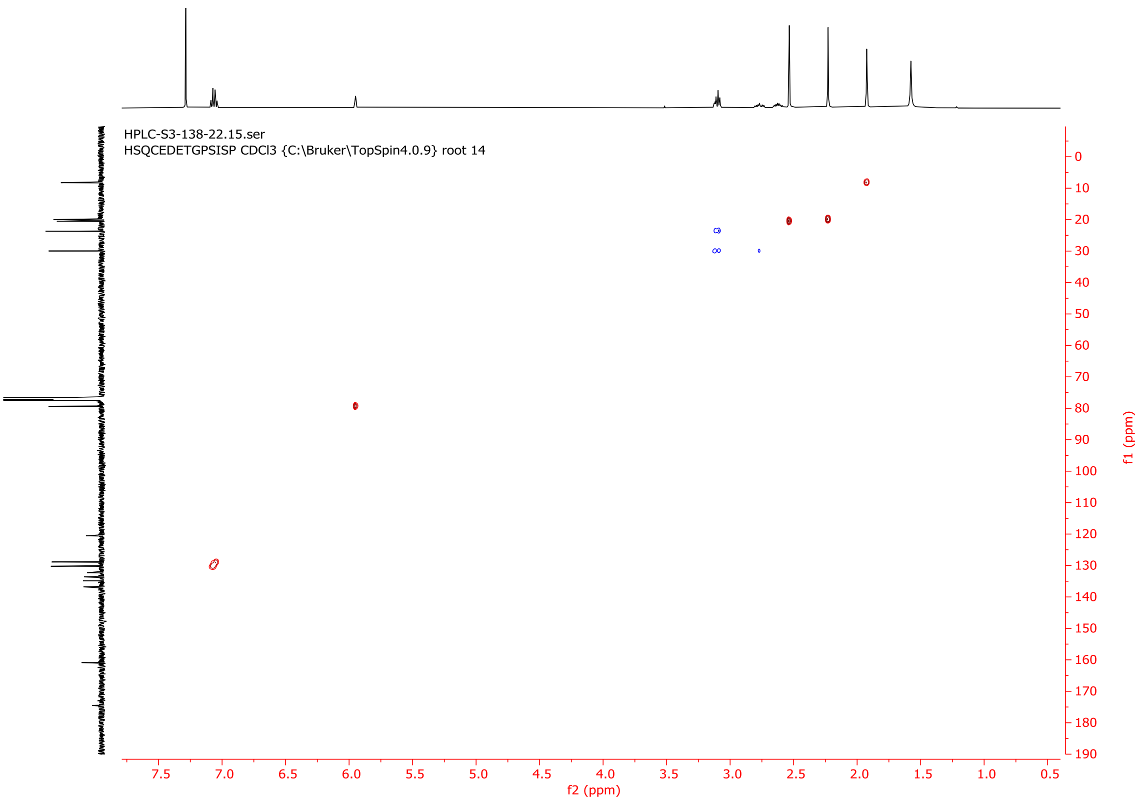

Figure S9. HSQC ($CDCl_3$, 400/100 MHz) of compound **12**

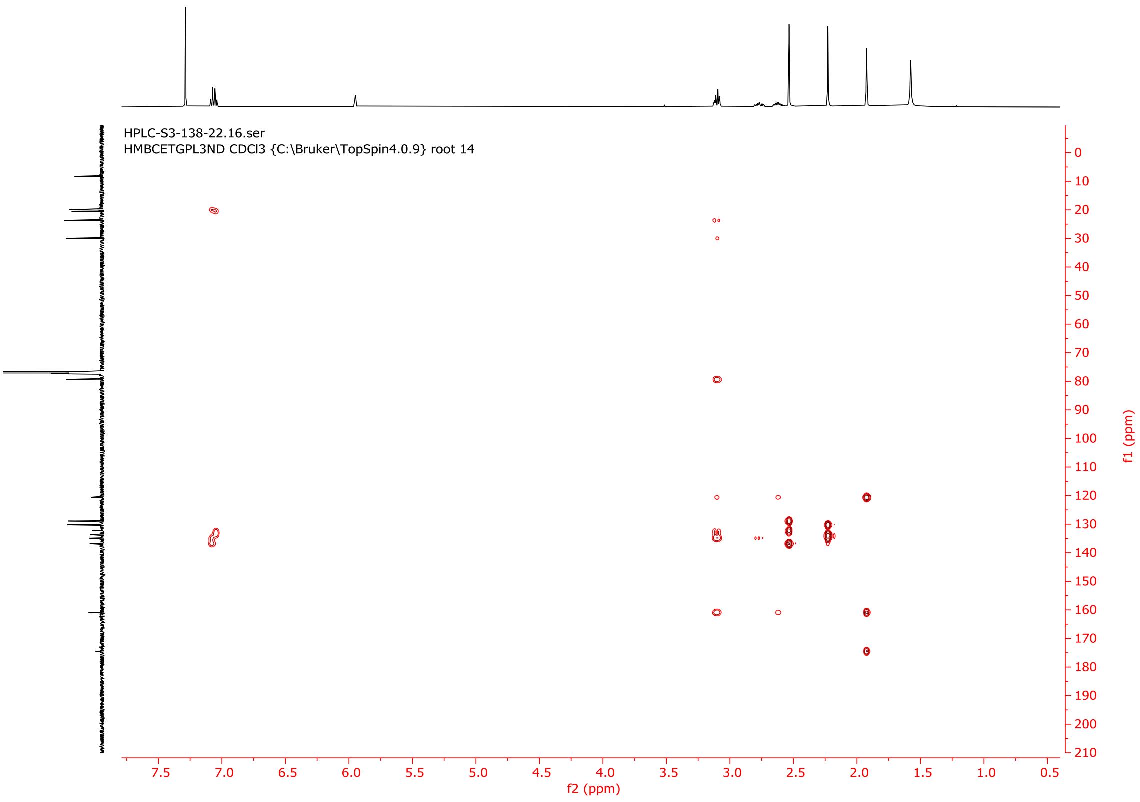

Figure S10. HMBC ($CDCl_3$, 400/100 MHz) of compound **12**

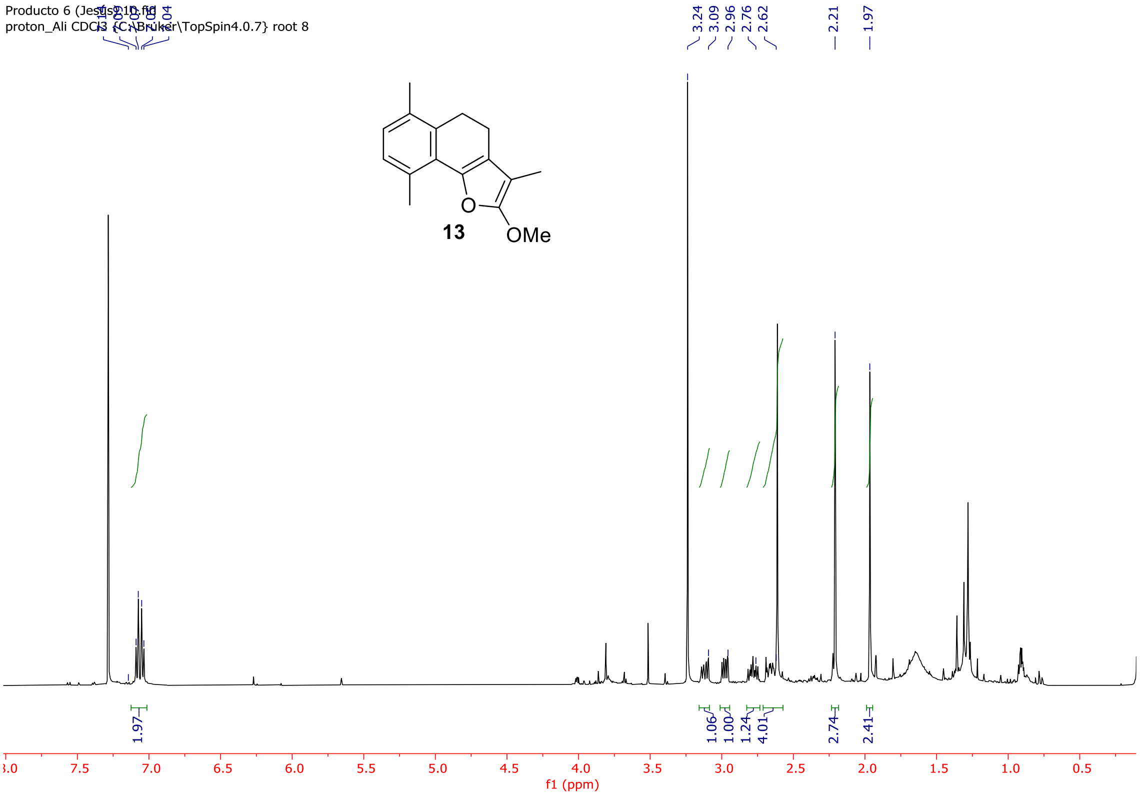

Figure S11. ^{1}H-NMR ($CDCl_3$, 400 MHz) spectrum of compound **13**

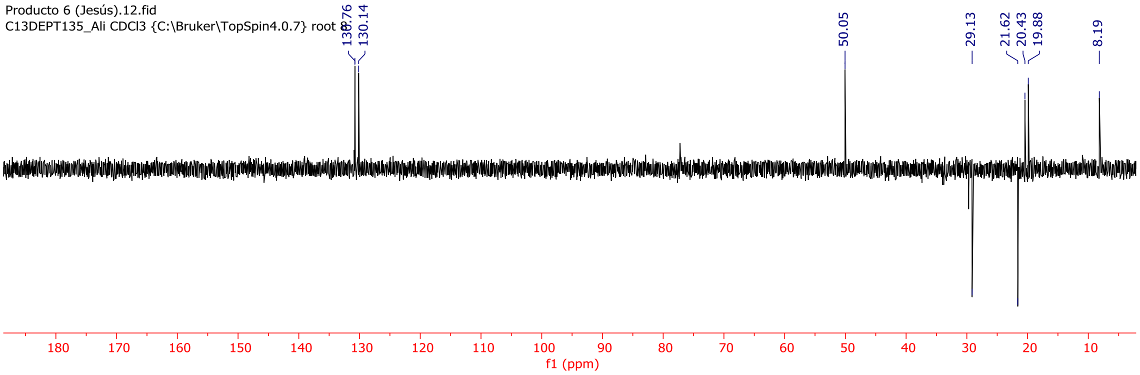

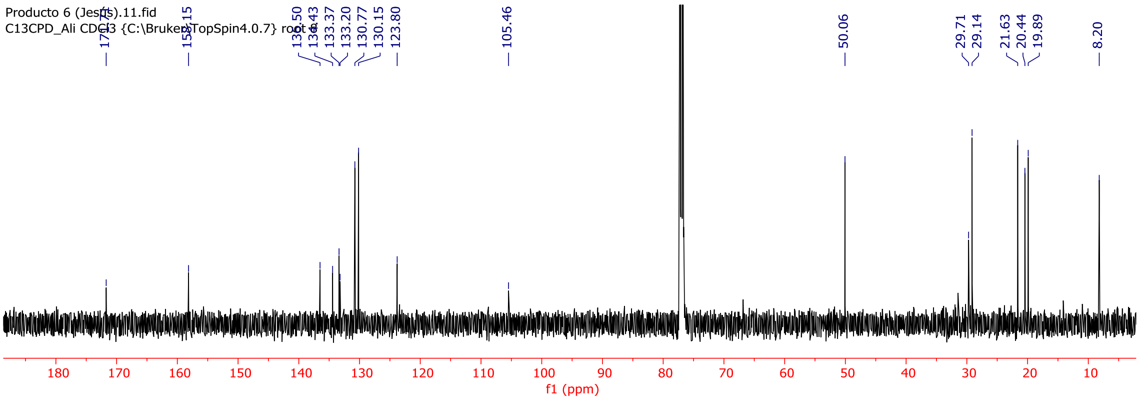

Figure S12. ^{13}C-NMR ($CDCl_3$, 100 MHz) spectrum of compound **13**

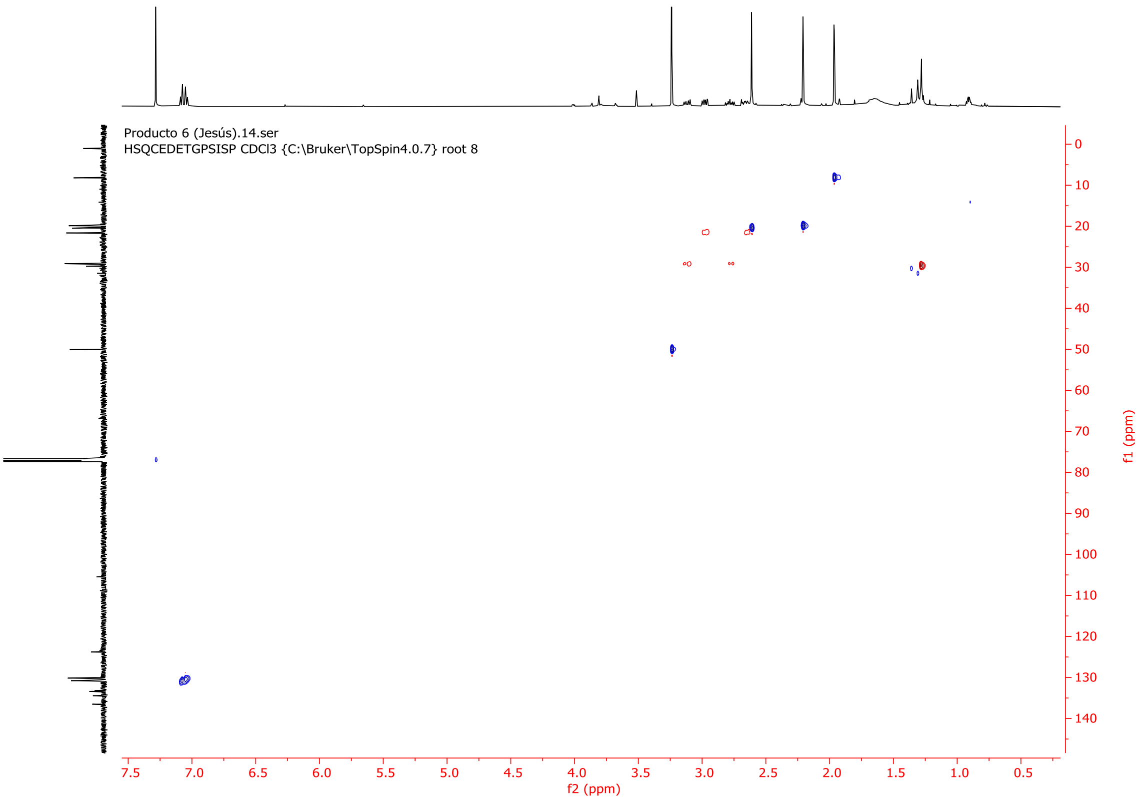

Figure S13. HSQC ($CDCl_3$, 400/100 MHz) of compound **13**

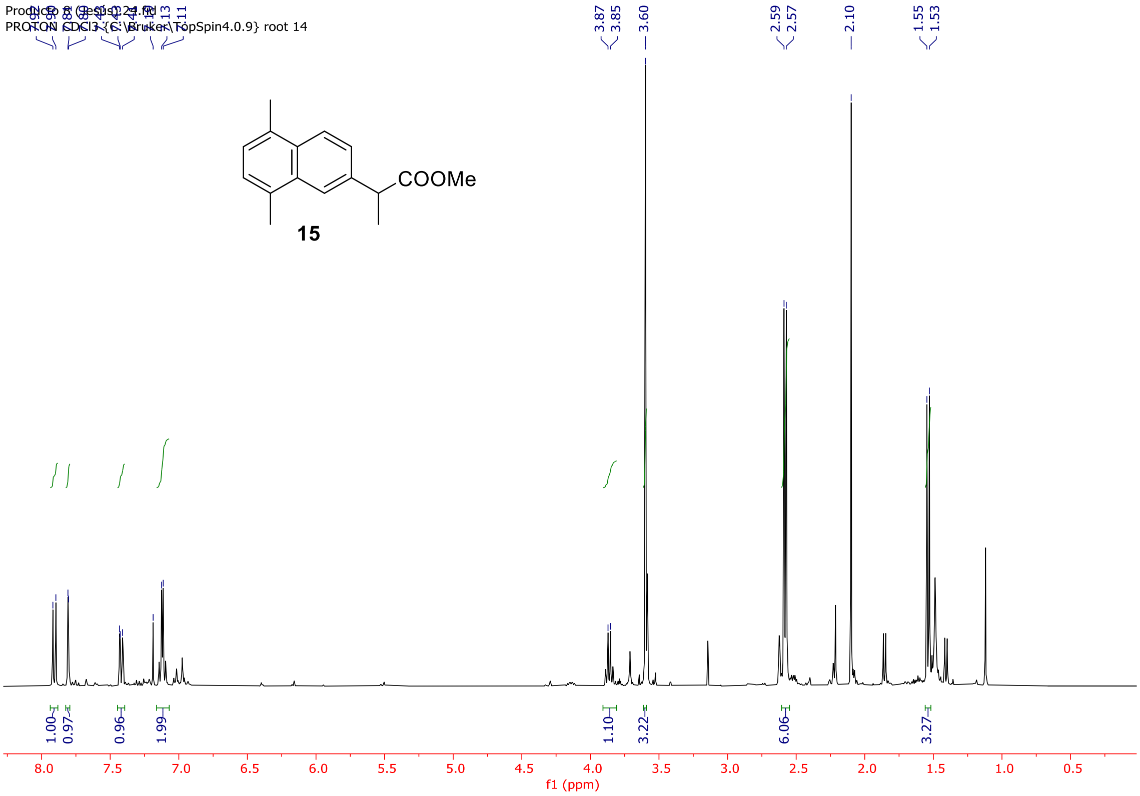

Figure S14. ^{1}H-NMR ($CDCl_3$, 400 MHz) spectrum of compound **15**

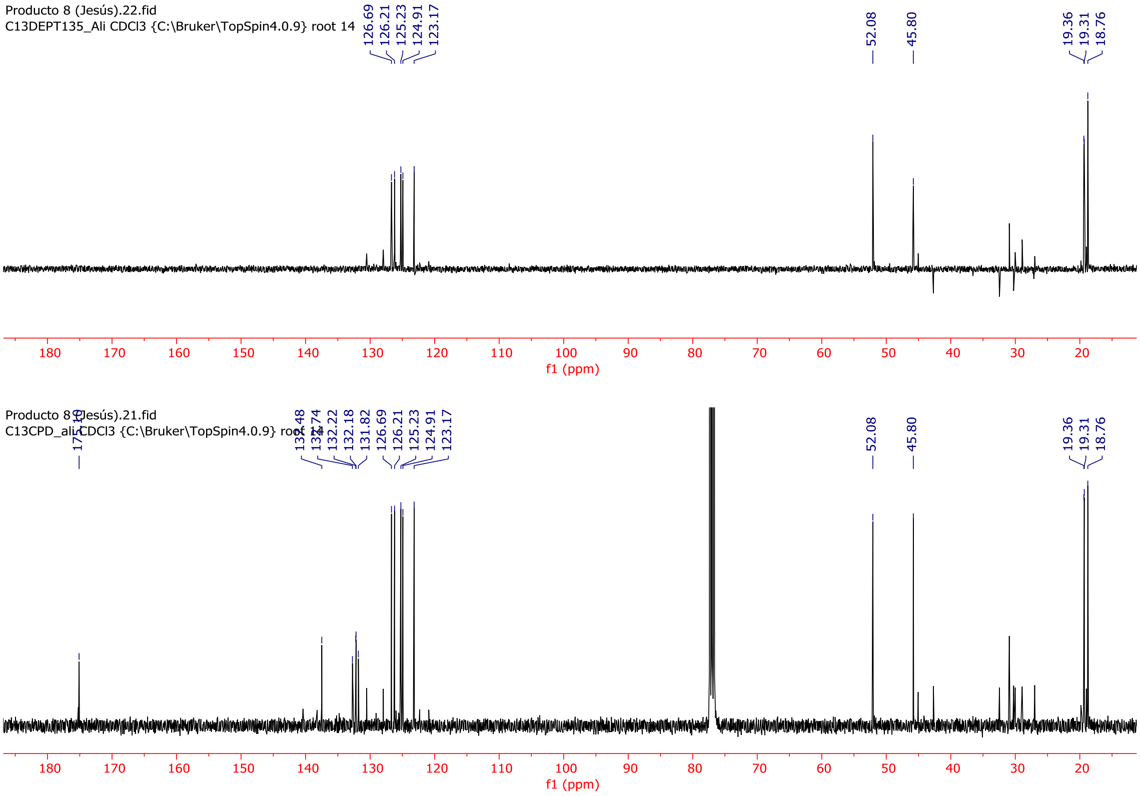

Figure S15. ^{13}C-NMR ($CDCl_3$, 100 MHz) spectrum of compound **15**

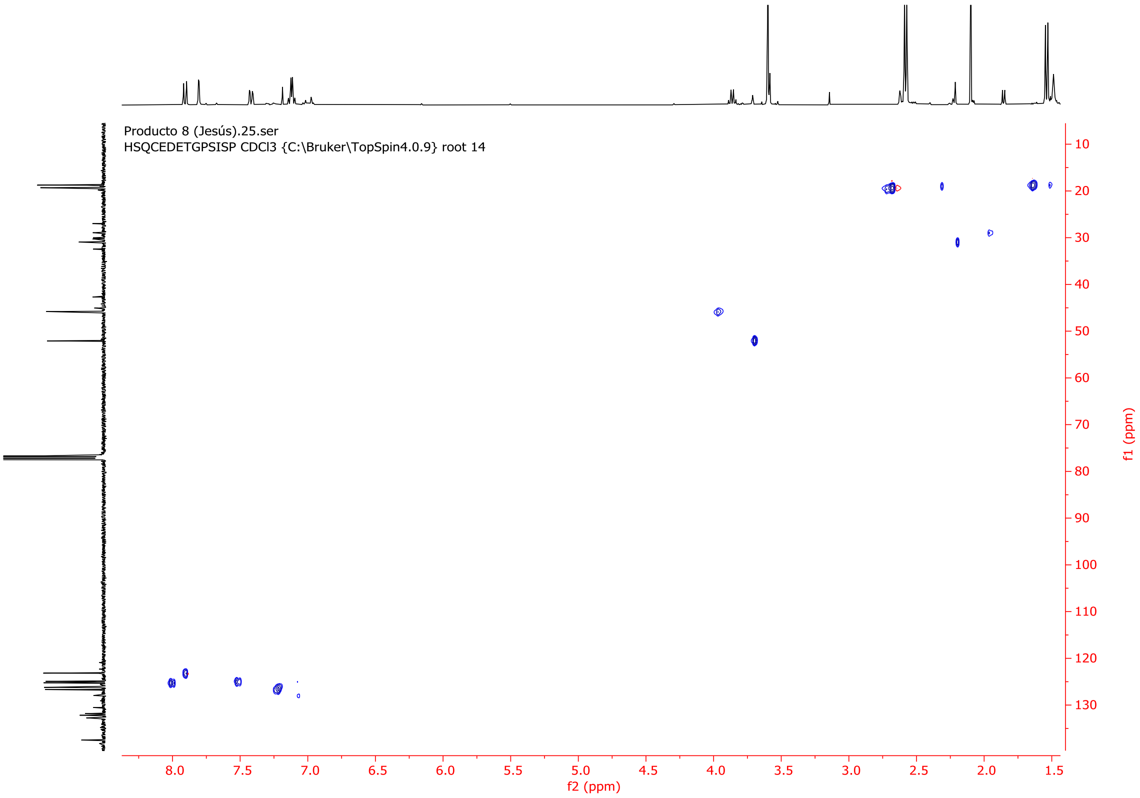

Figure S16. HSQC ($CDCl_3$, 400/100 MHz) of compound **15**

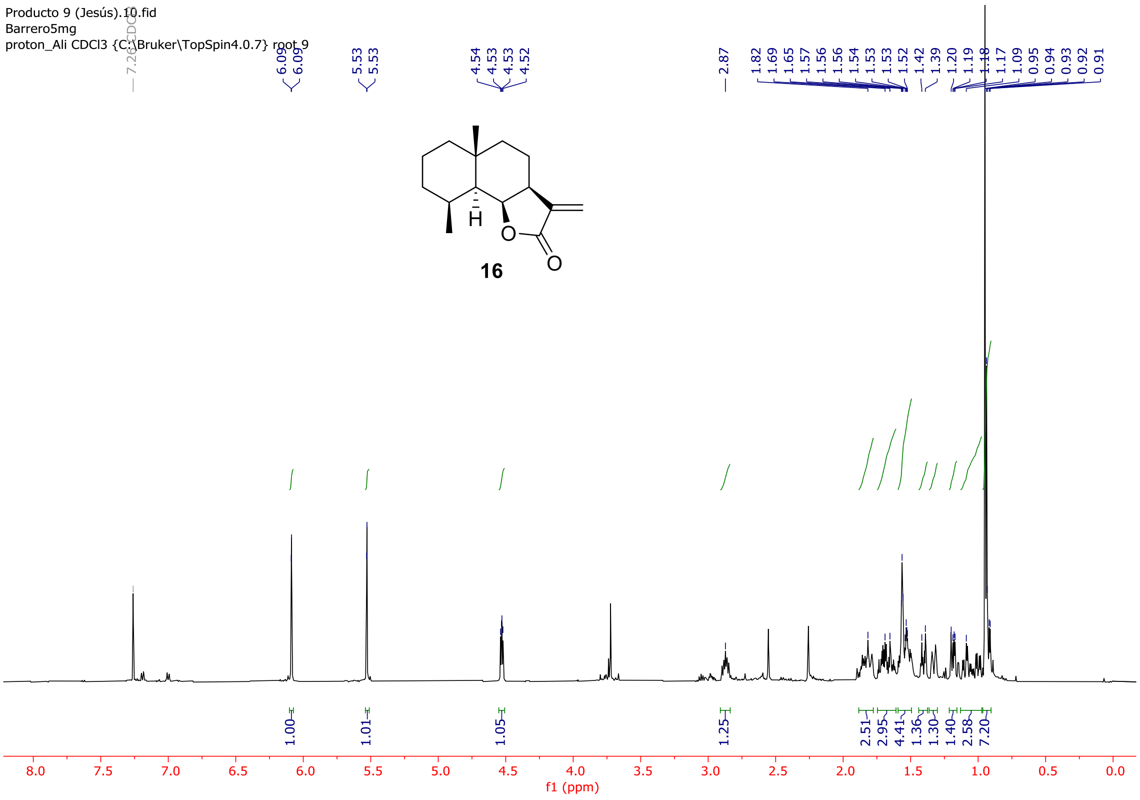

Figure S17. ^{1}H-NMR ($CDCl_3$, 400 MHz) spectrum of compound **16**

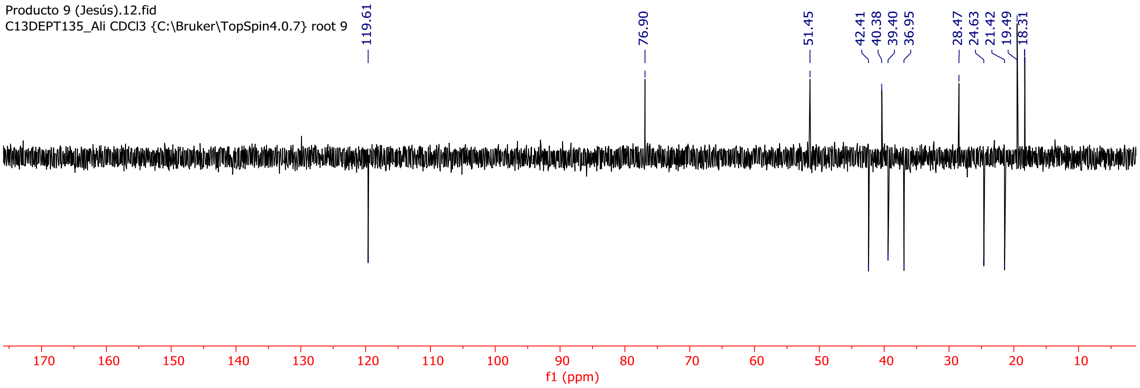

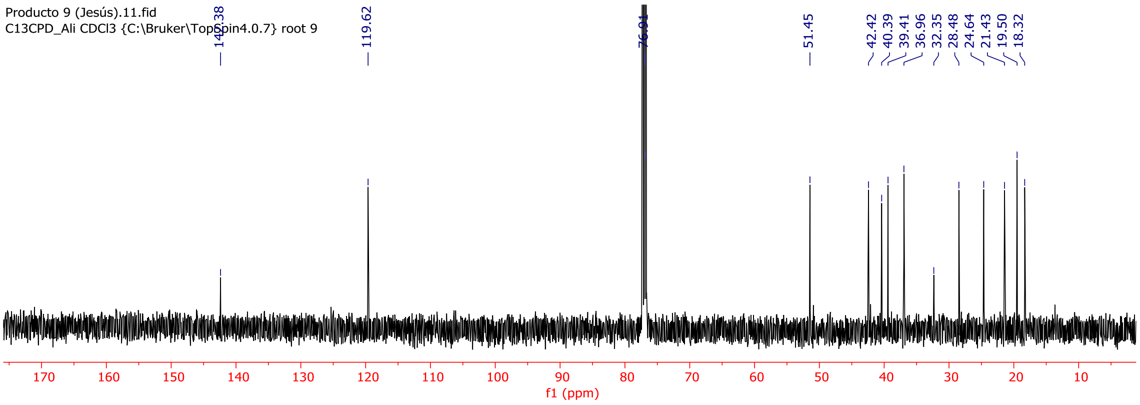

Figure S18. ^{13}C-NMR ($CDCl_3$, 100 MHz) spectrum of compound **16**

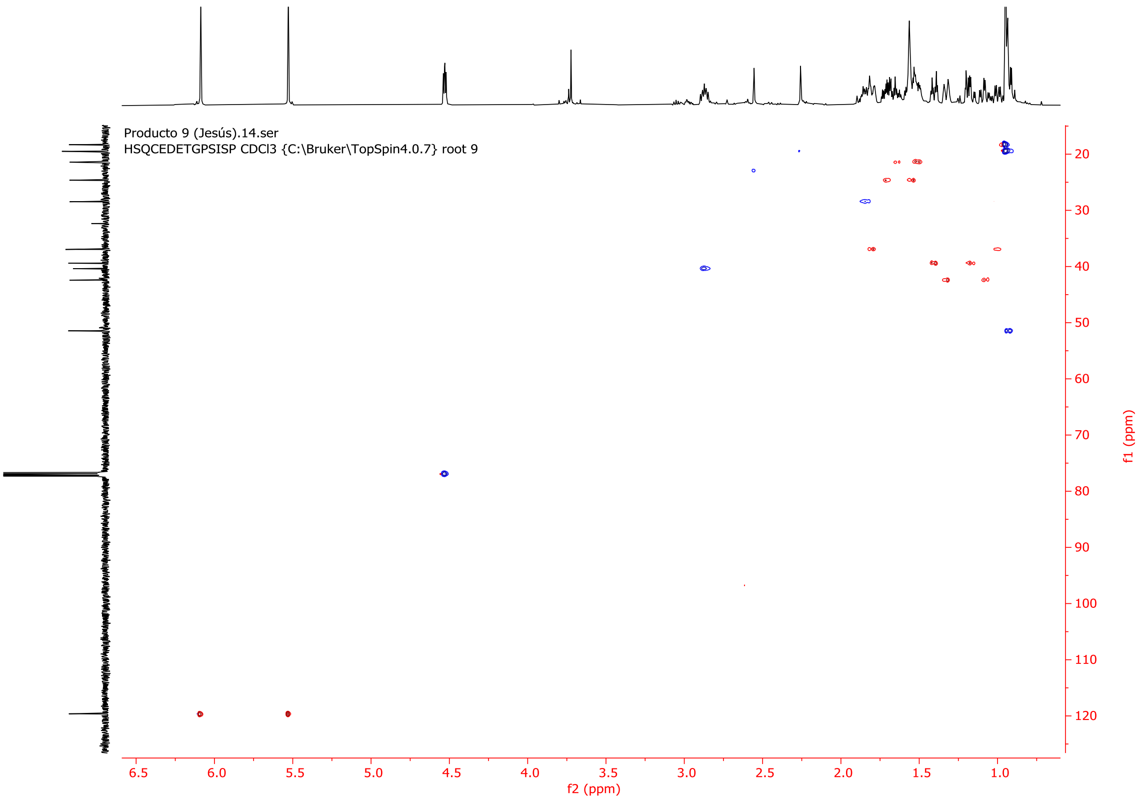

Figure S19. HSQC ($CDCl_3$, 400/100 MHz) of compound **16**

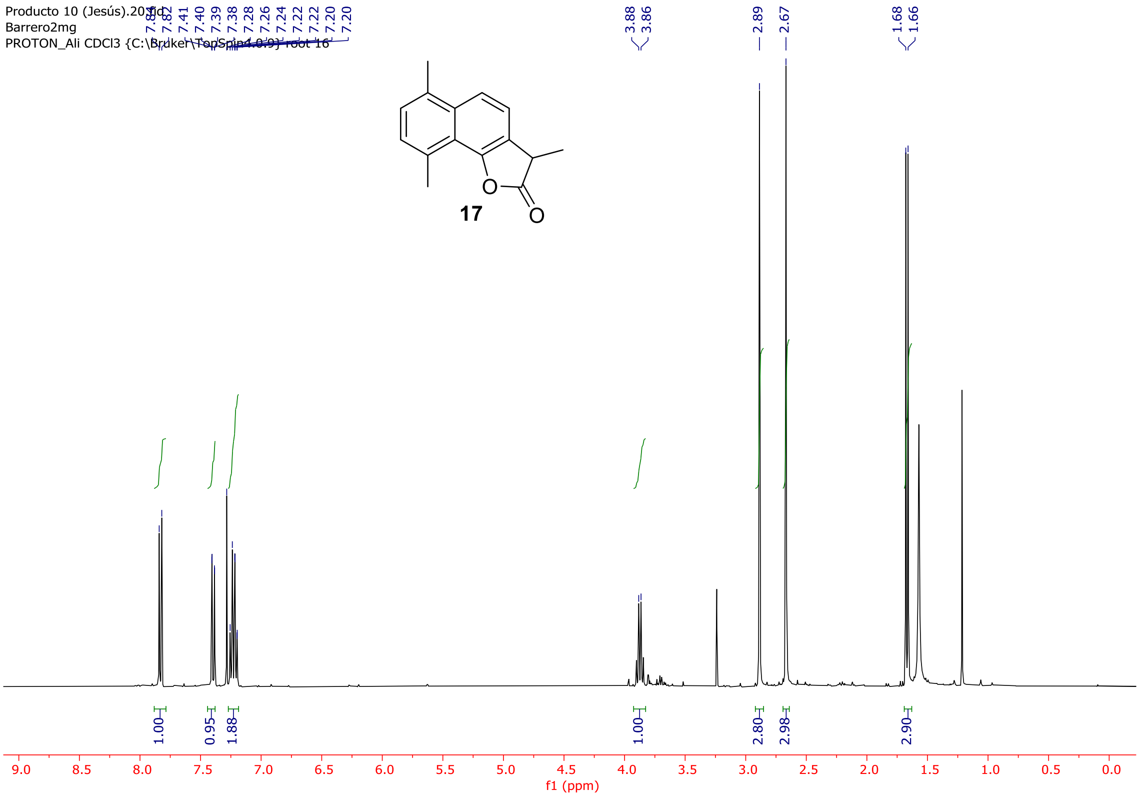

Figure S20. ^{1}H-NMR ($CDCl_3$, 400 MHz) spectrum of compound **17**

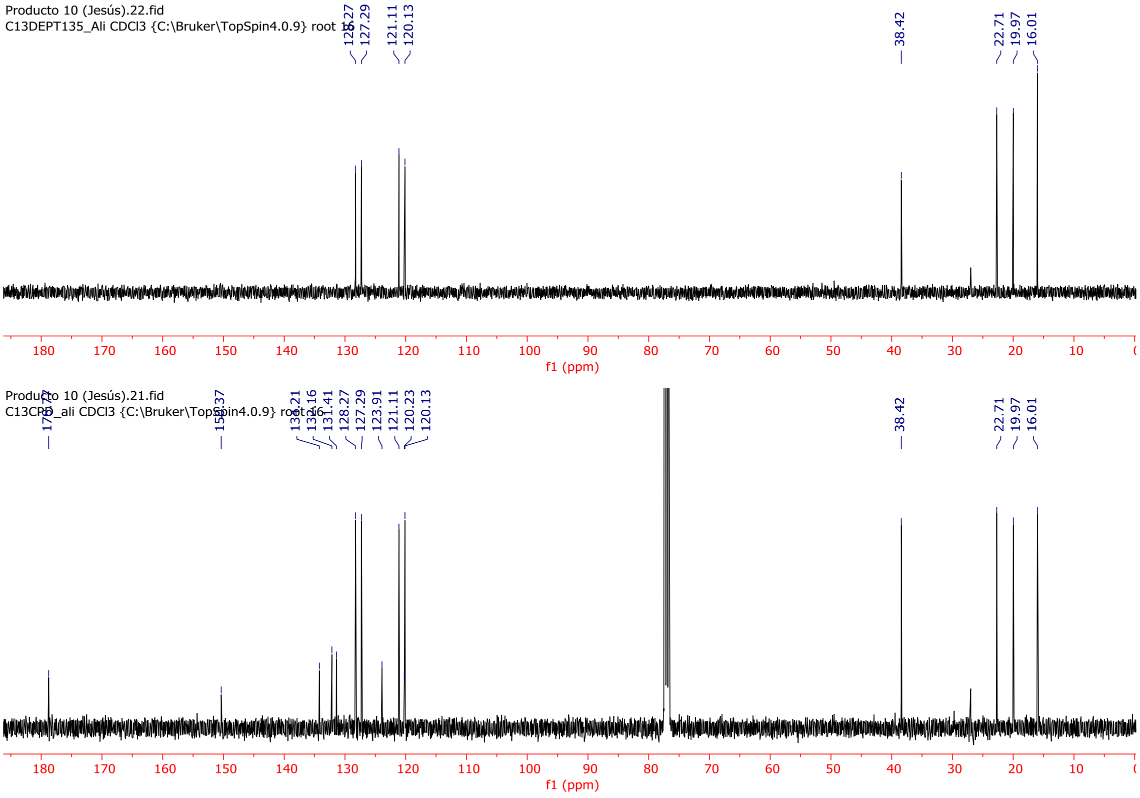

Figure S21. ^{13}C-NMR ($CDCl_3$, 100 MHz) spectrum of compound **17**

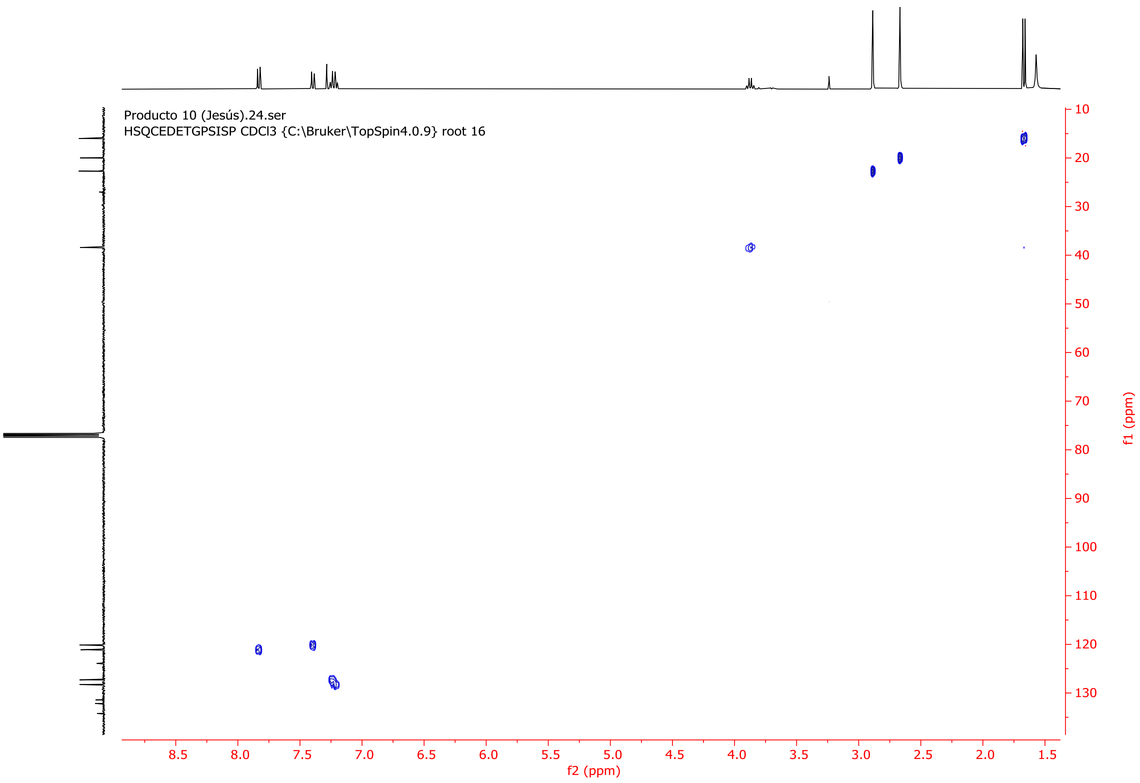

Figure S22. HSQC ($CDCl_3$, 400/100 MHz) of compound **17**

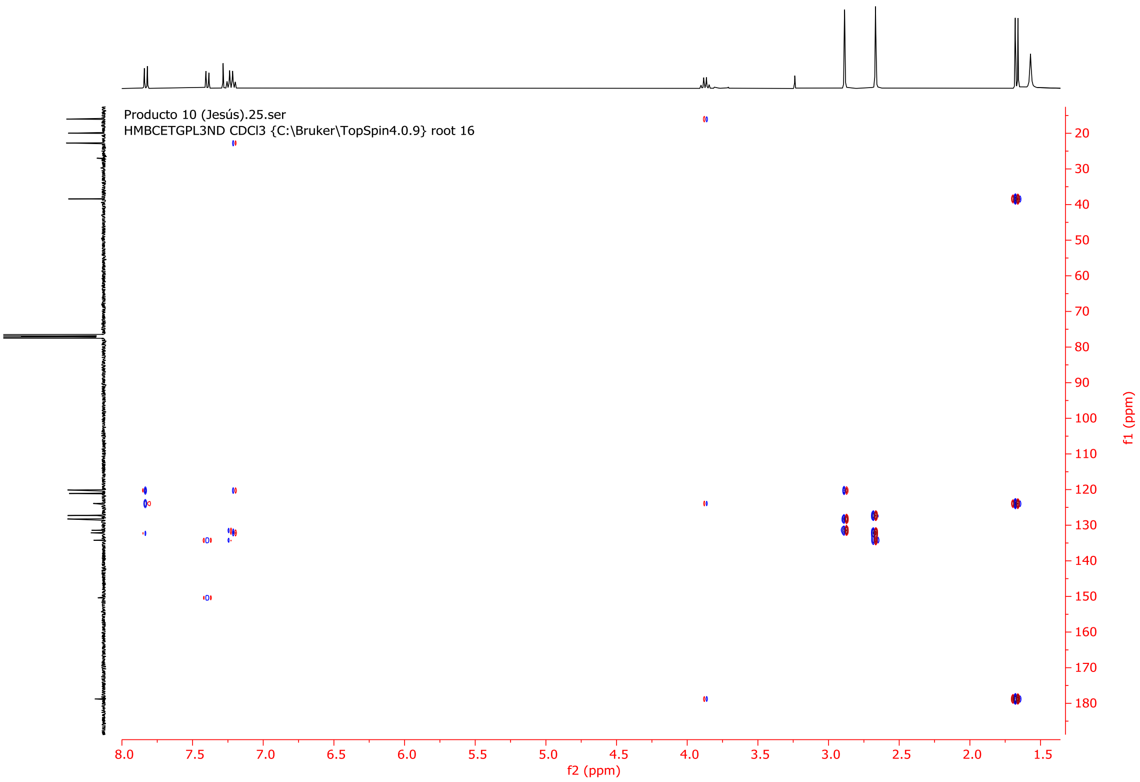

Figure S23. HMBC ($CDCl_3$, 400/100 MHz) of compound **17**

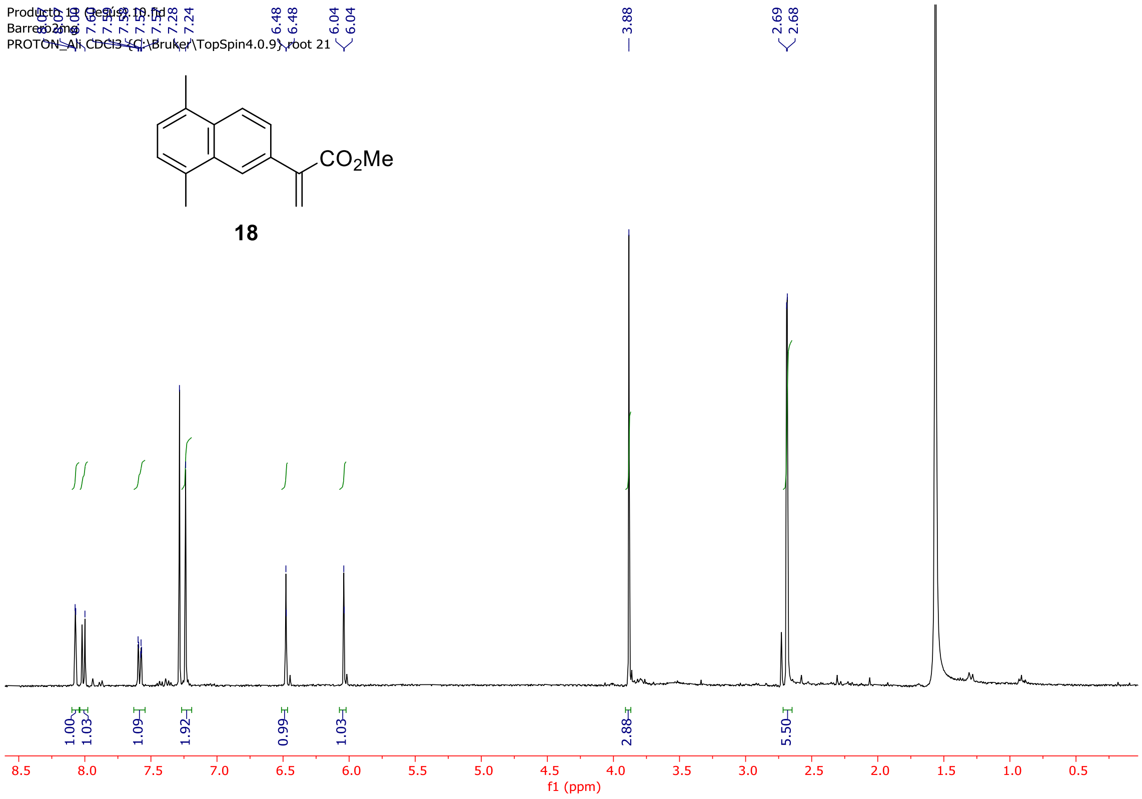

Figure S24. ^{1}H-NMR ($CDCl_3$, 400 MHz) spectrum of compound **18**

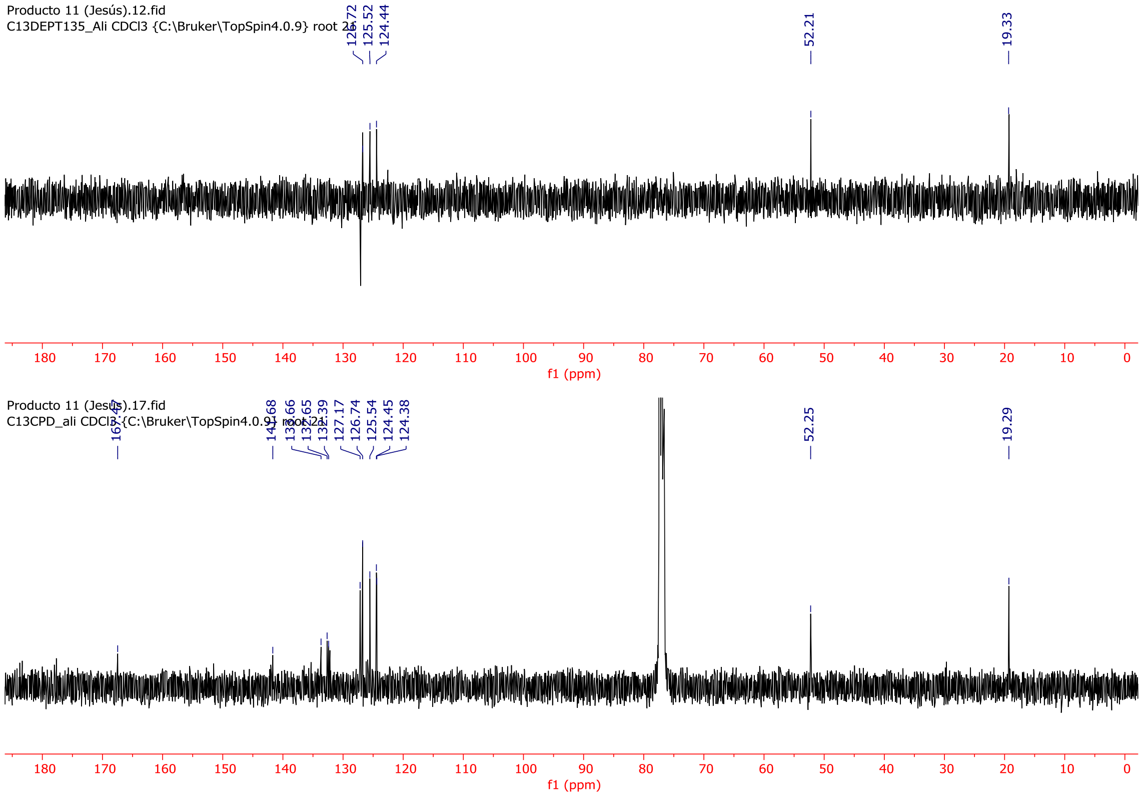

Figure S25. ^{13}C-NMR ($CDCl_3$, 100 MHz) spectrum of compound **18**

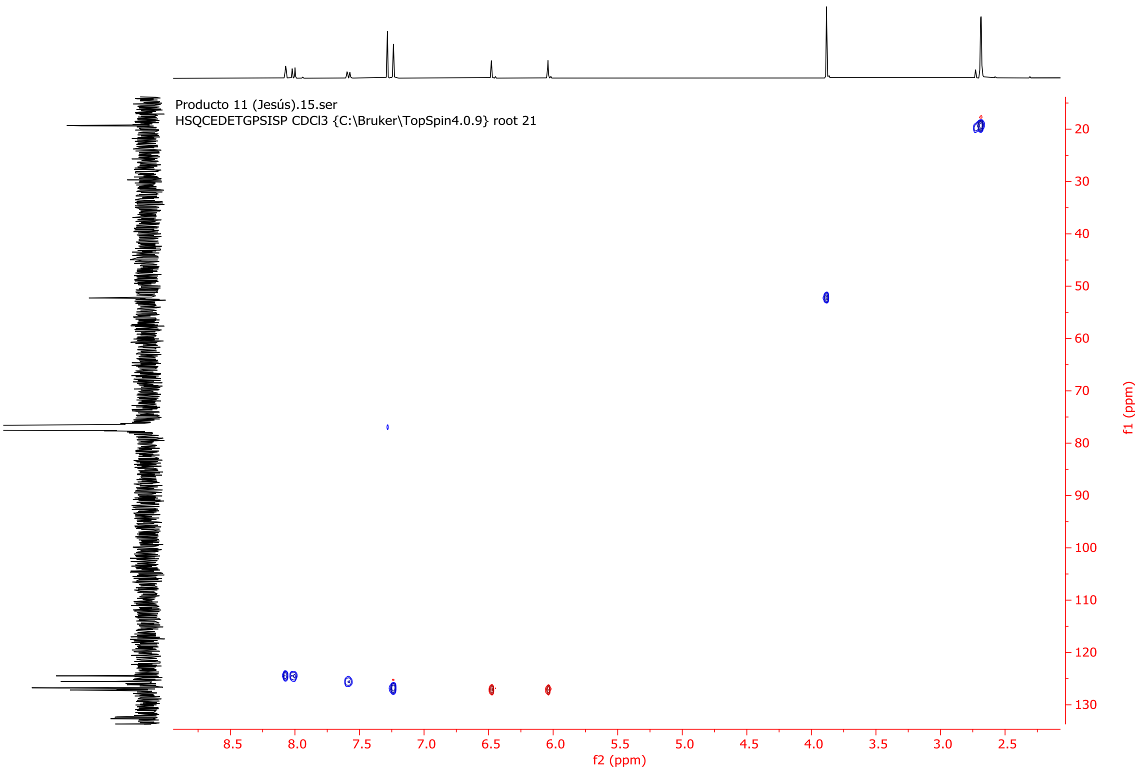

Figure 26. HSQC ($CDCl_3$, 400/100 MHz) of compound **18**

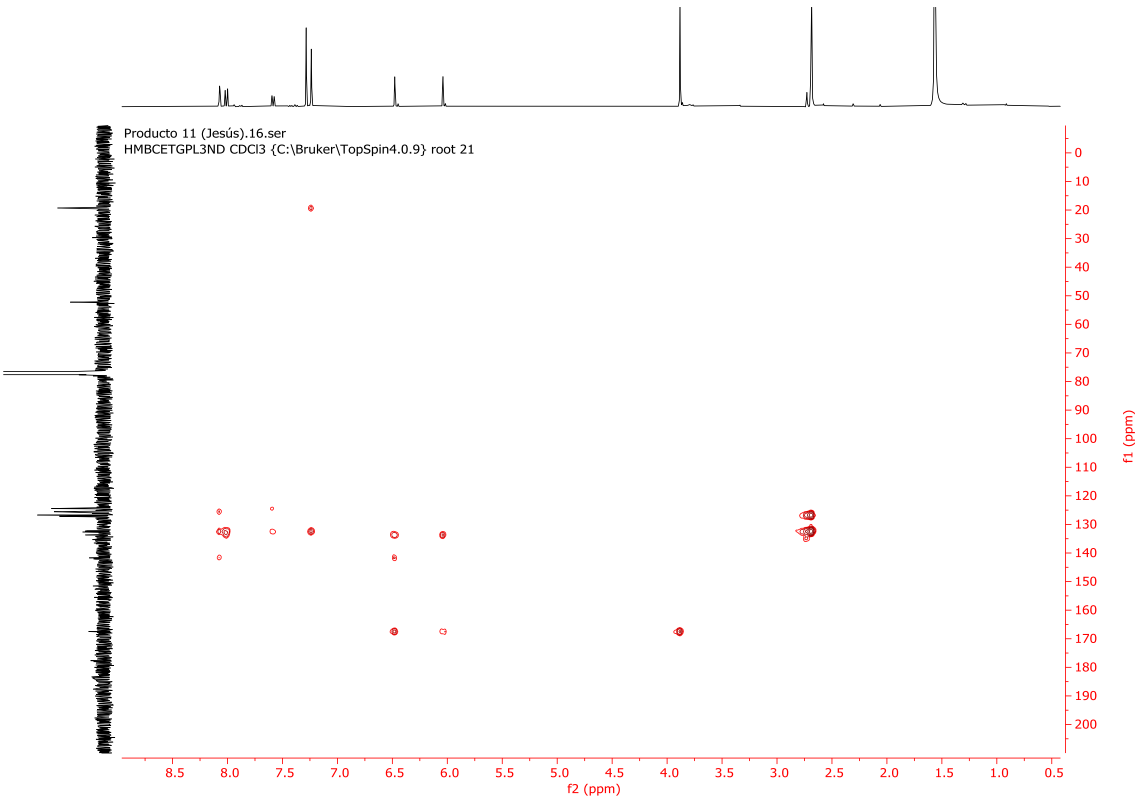

Figure 27. HMBC ($CDCl_3$, 400/100 MHz) of compound **18**

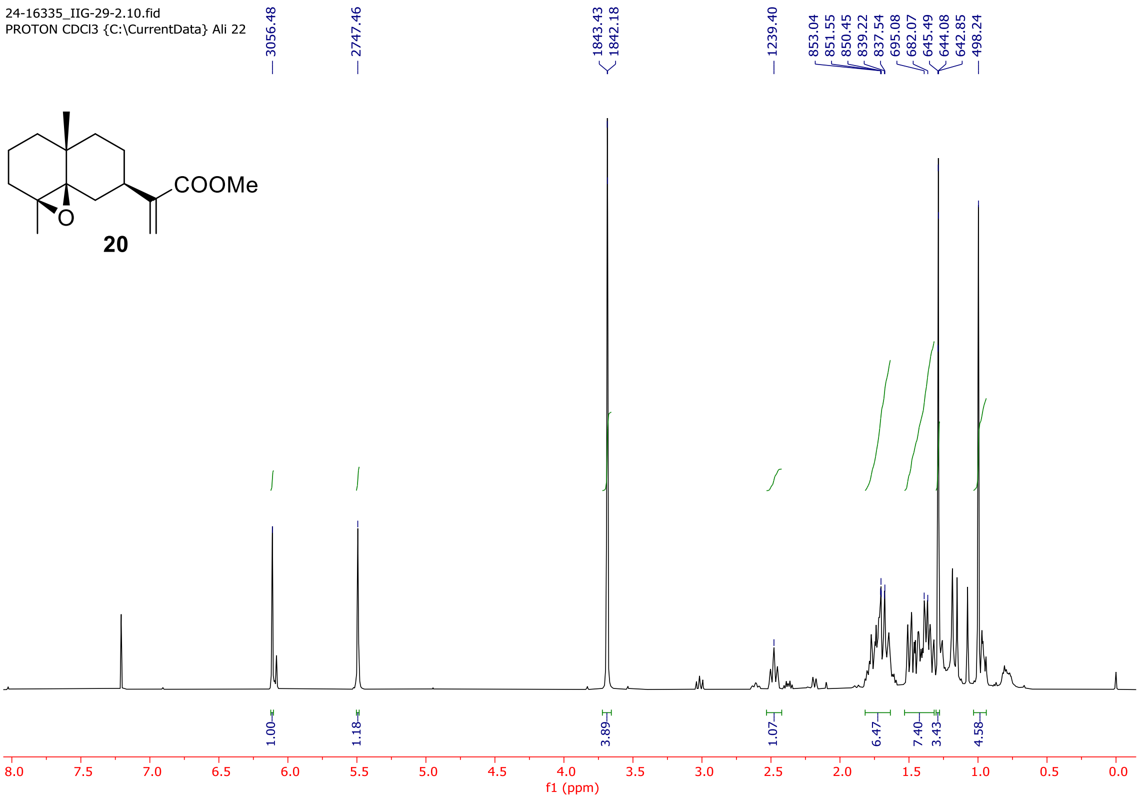

Figure S28. ^{1}H-NMR ($CDCl_3$, 400 MHz) spectrum of compound **20**

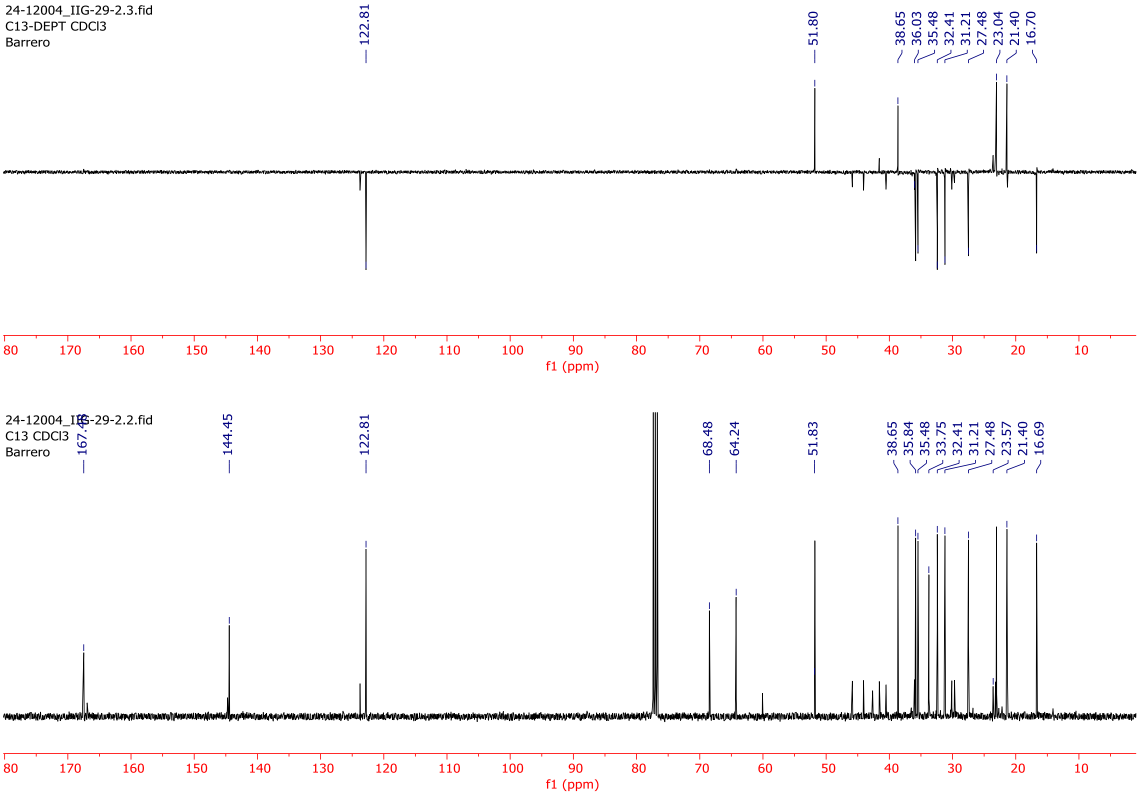

Figure S29. ^{13}C-NMR ($CDCl_3$, 100 MHz) spectrum of compound **20**

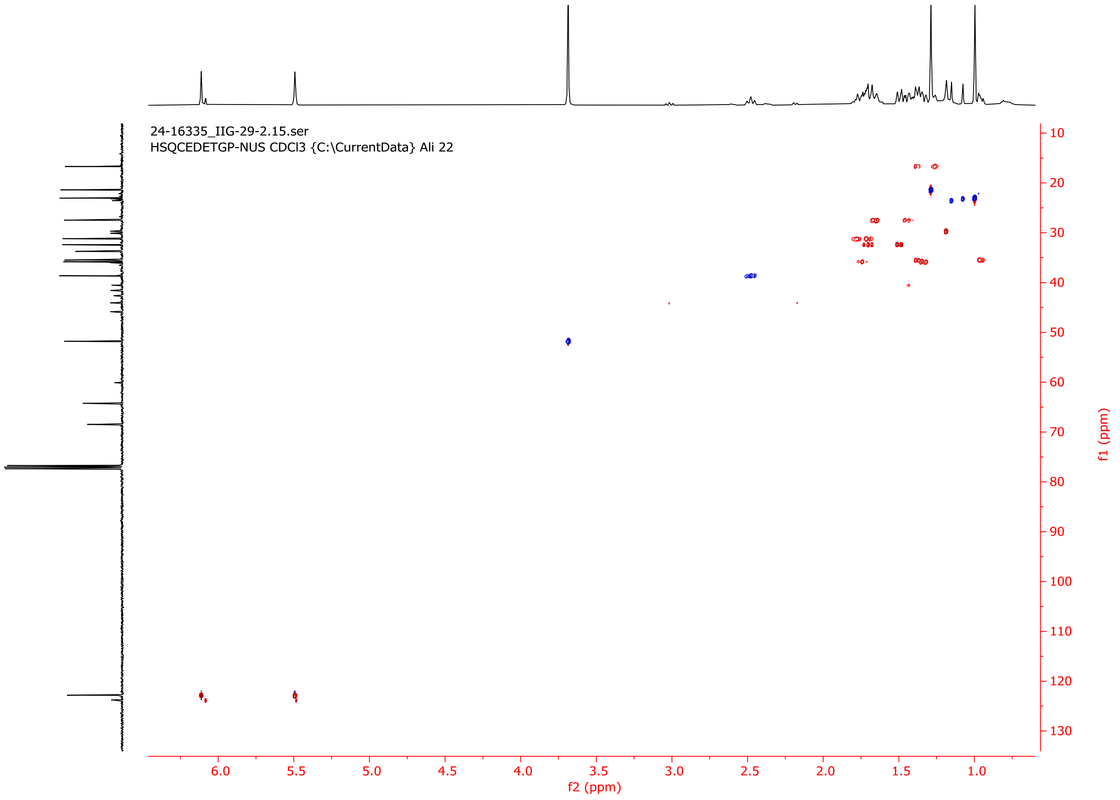

Figure S30. HSQC ($CDCl_3$, 400/100 MHz) of compound **20**

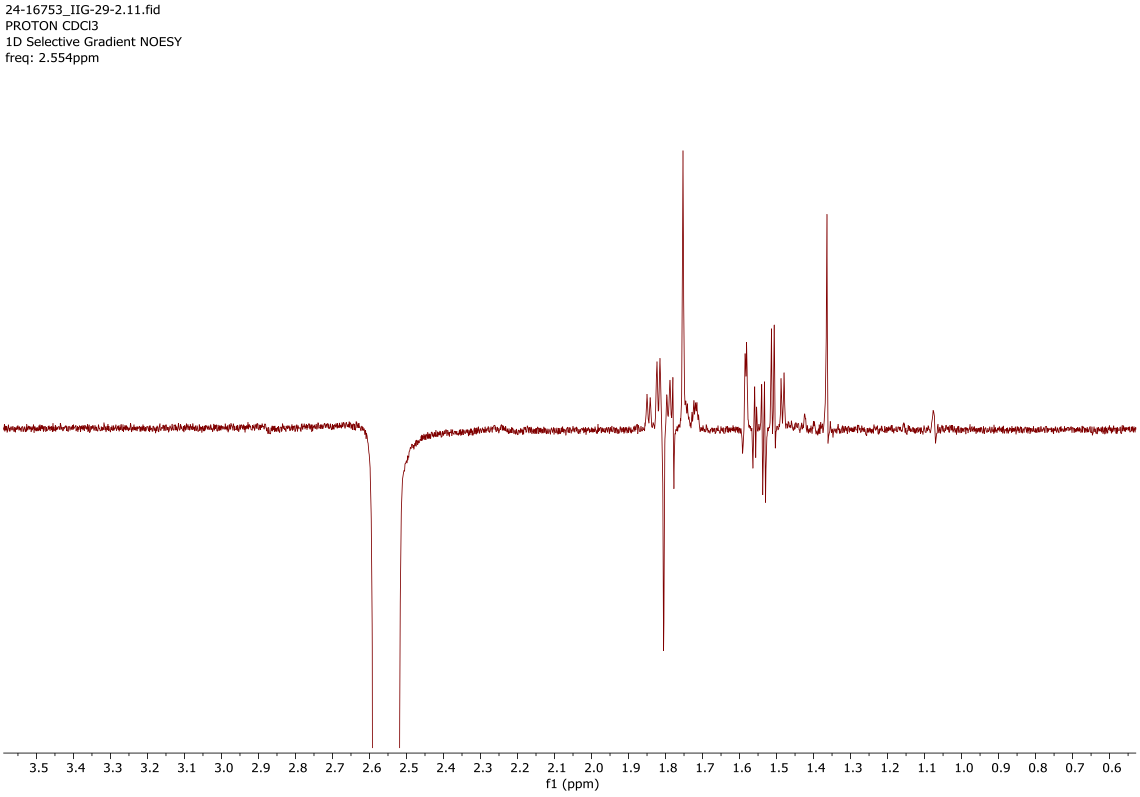

Figure S31. NOEDIFF ($CDCl_3$, 100 MHz) of compound **20**

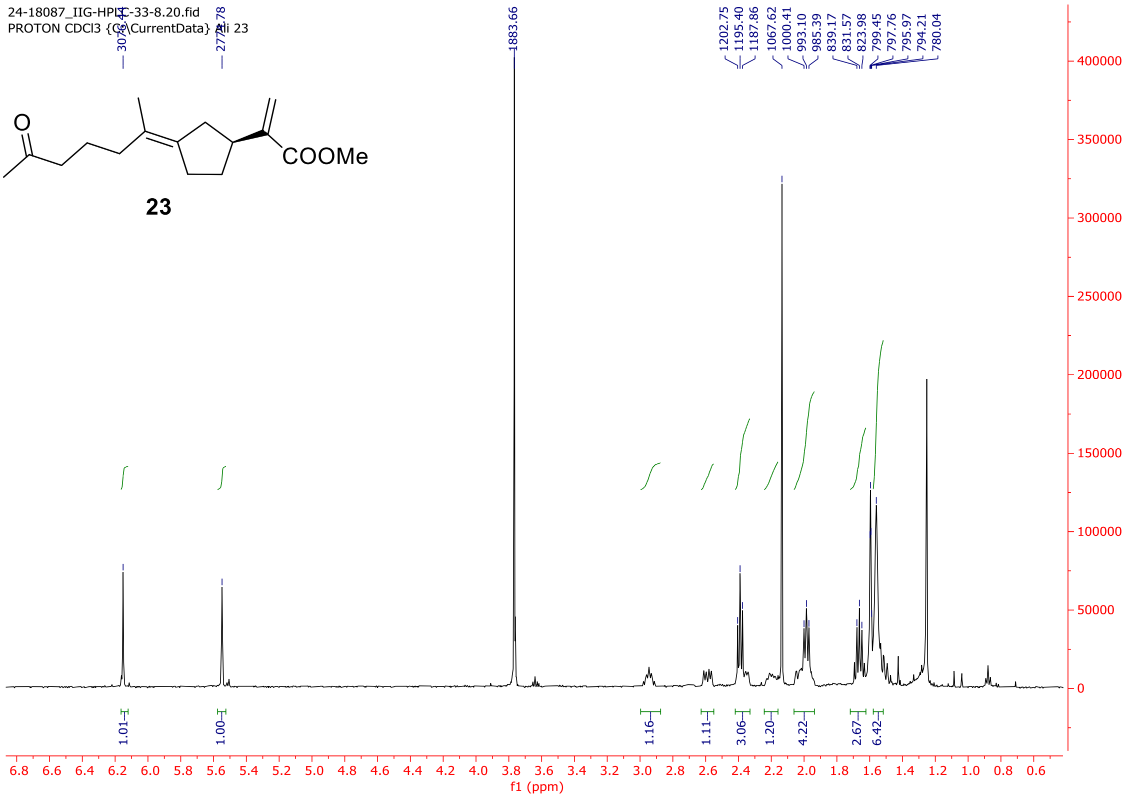

Figure S32. ^{1}H-NMR ($CDCl_3$, 400 MHz) spectrum of compound **23**

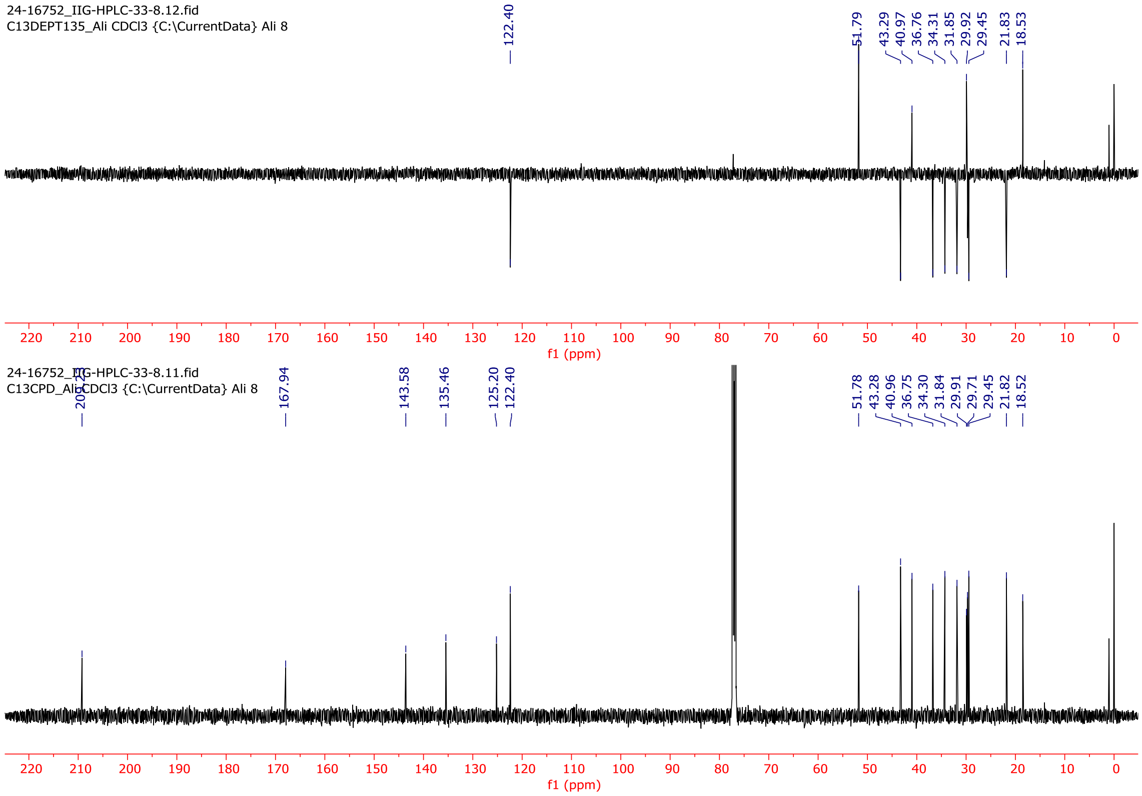

Figure S33. ^{13}C-NMR ($CDCl_3$, 100 MHz) spectrum of compound **23**

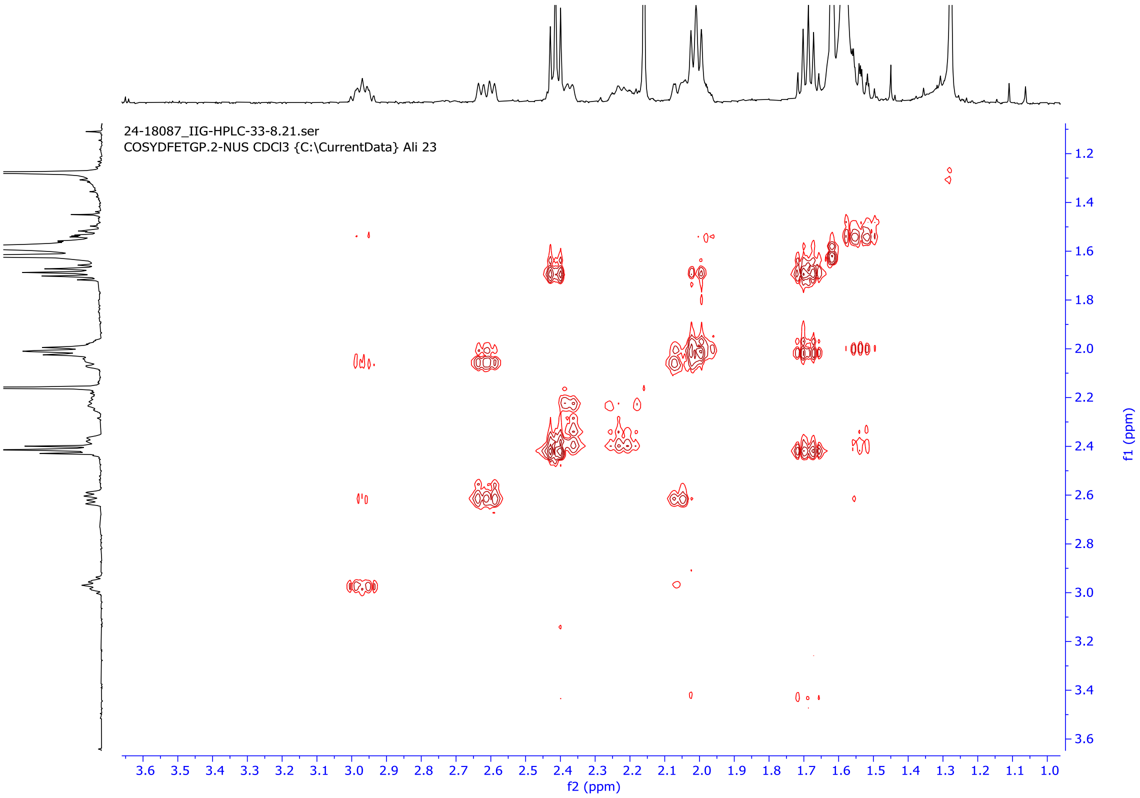

Figure S34. COSY ($CDCl_3$, 400/400 MHz) of compound **23**

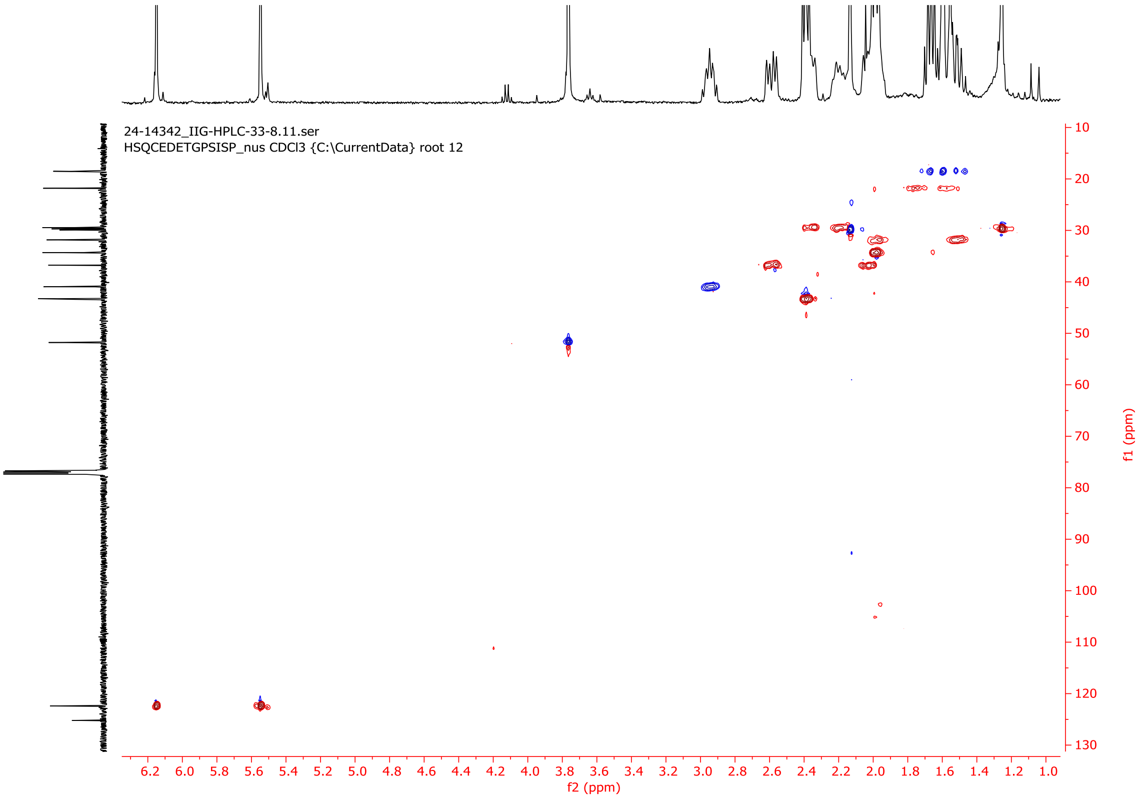

Figure S35. HSQC ($CDCl_3$, 400/100 MHz) of compound **23**

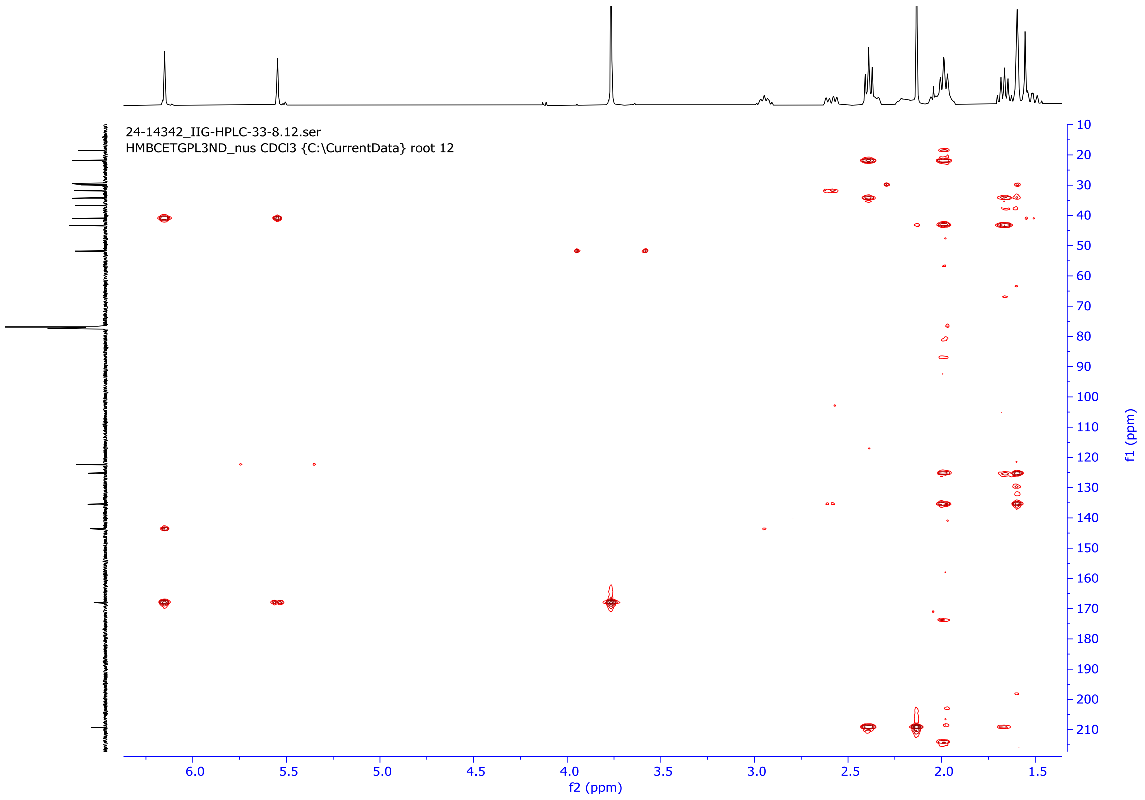

Figure S36. HMBC ($CDCl_3$, 400/100 MHz) of compound **23**

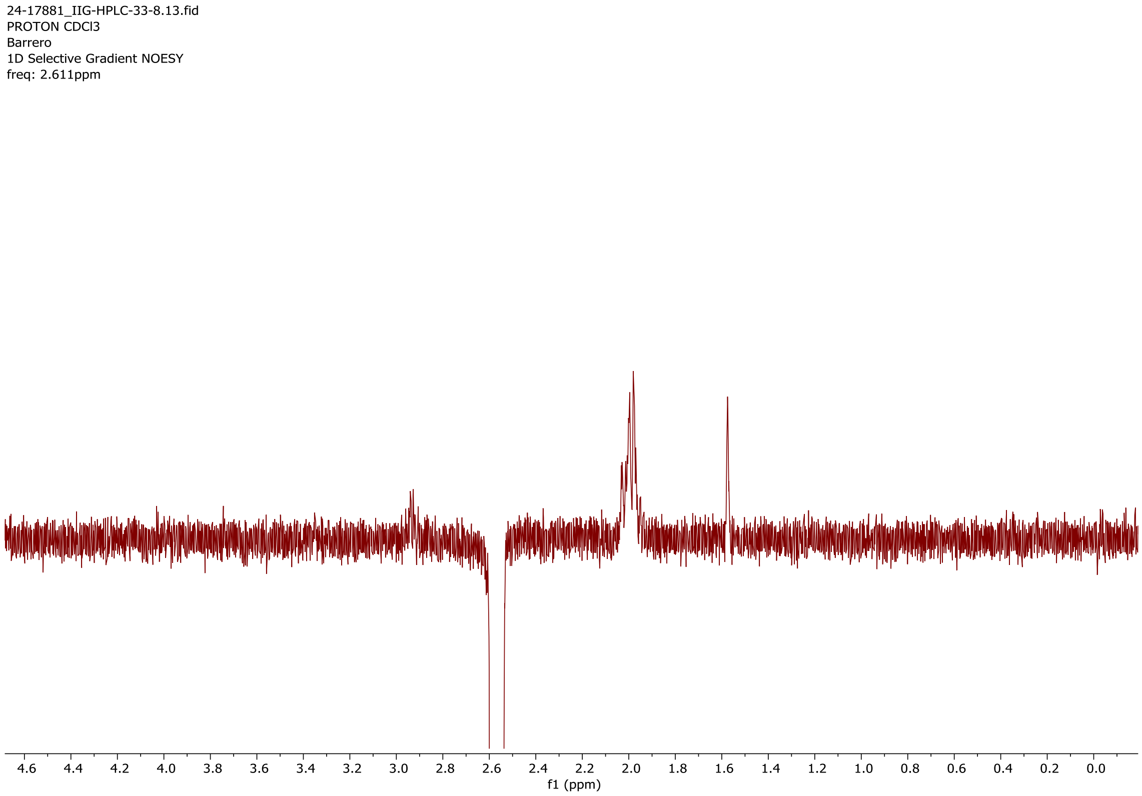

Figure S37. NOEDIFF ($CDCl_3$, 100 MHz) of compound **23**

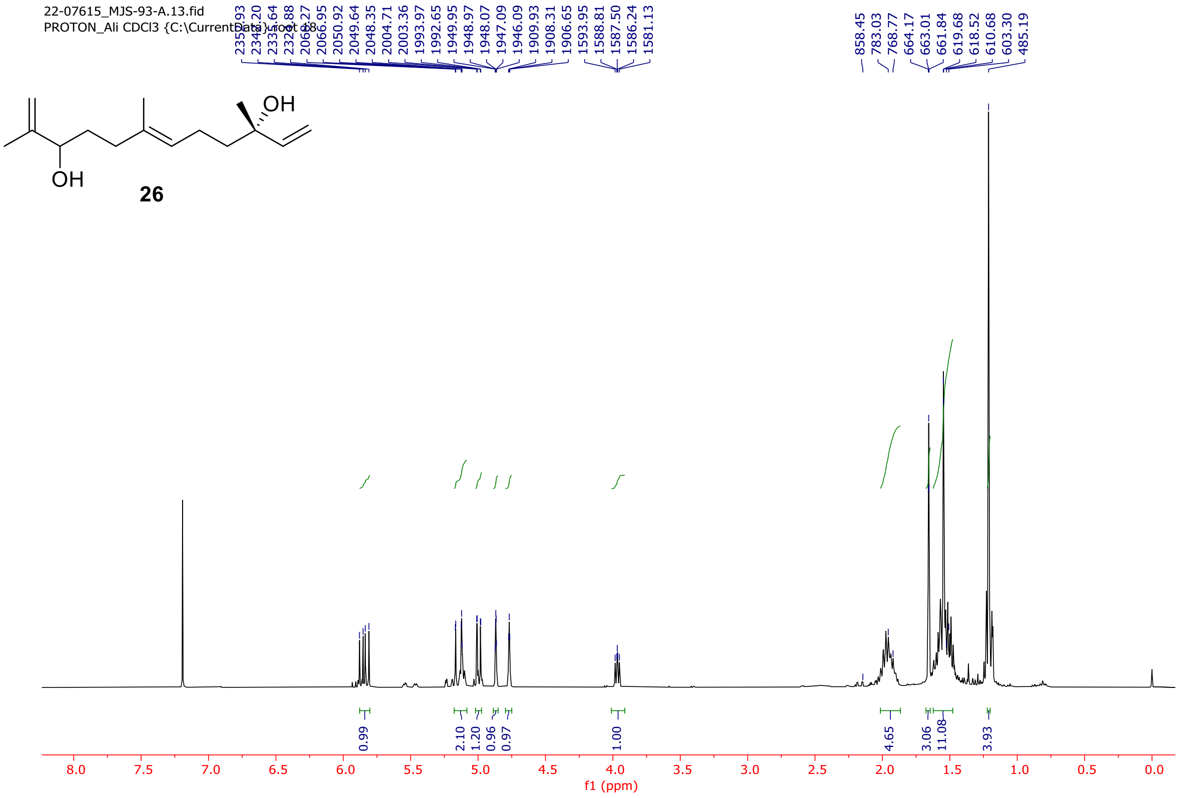

Figure S38. ^{1}H-NMR ($CDCl_3$, 400 MHz) spectrum of compound **26**

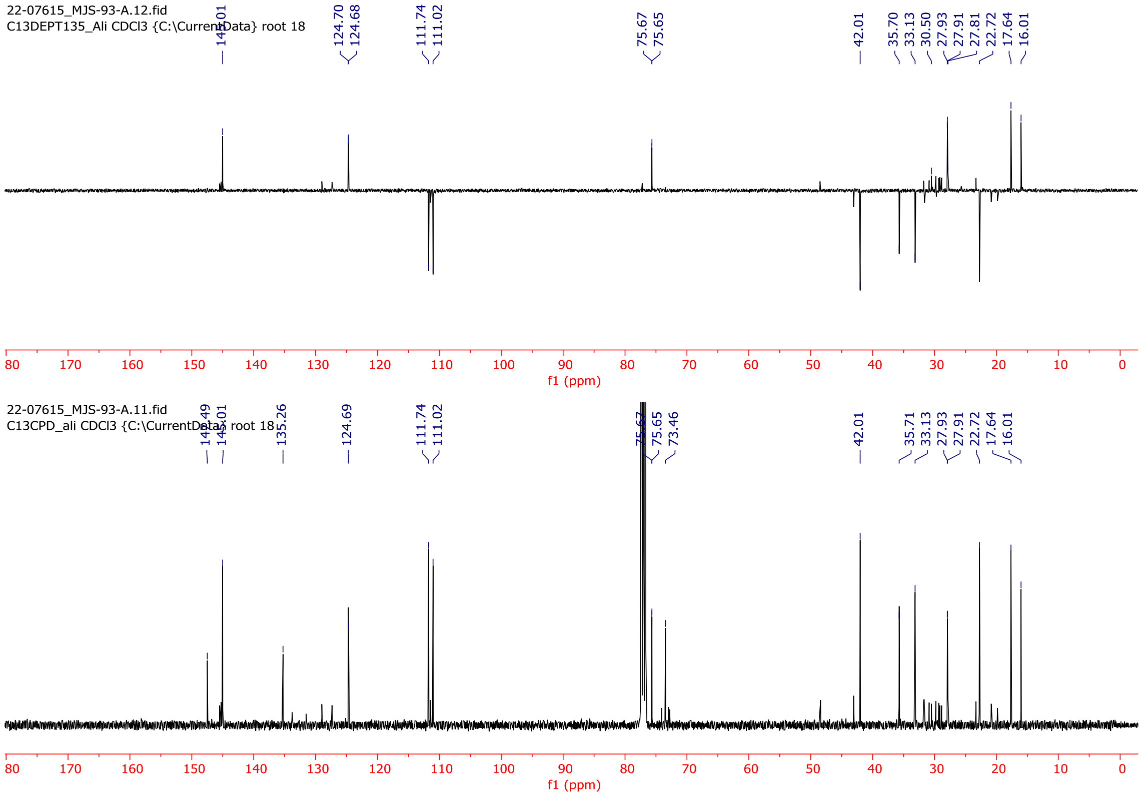

Figure S39. ^{13}C-NMR ($CDCl_3$, 100 MHz) spectrum of compound **26**

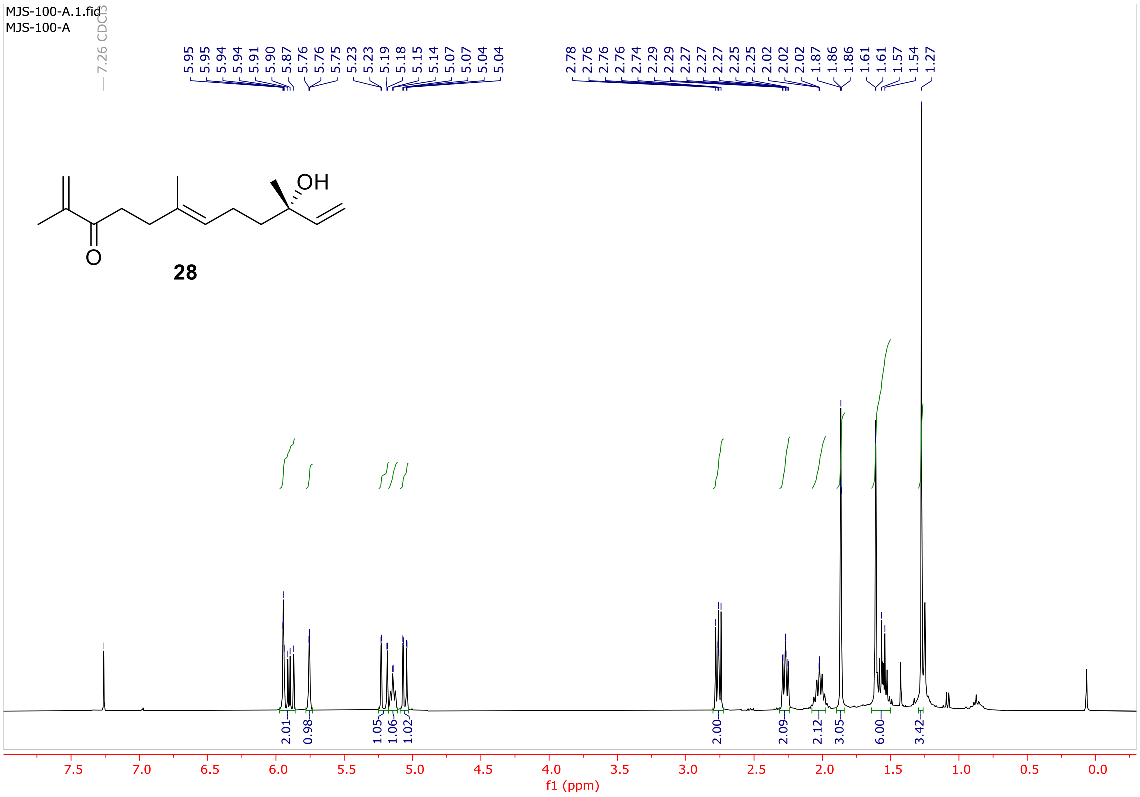

Figure S40. ^{1}H-NMR ($CDCl_3$, 400 MHz) spectrum of compound **28**

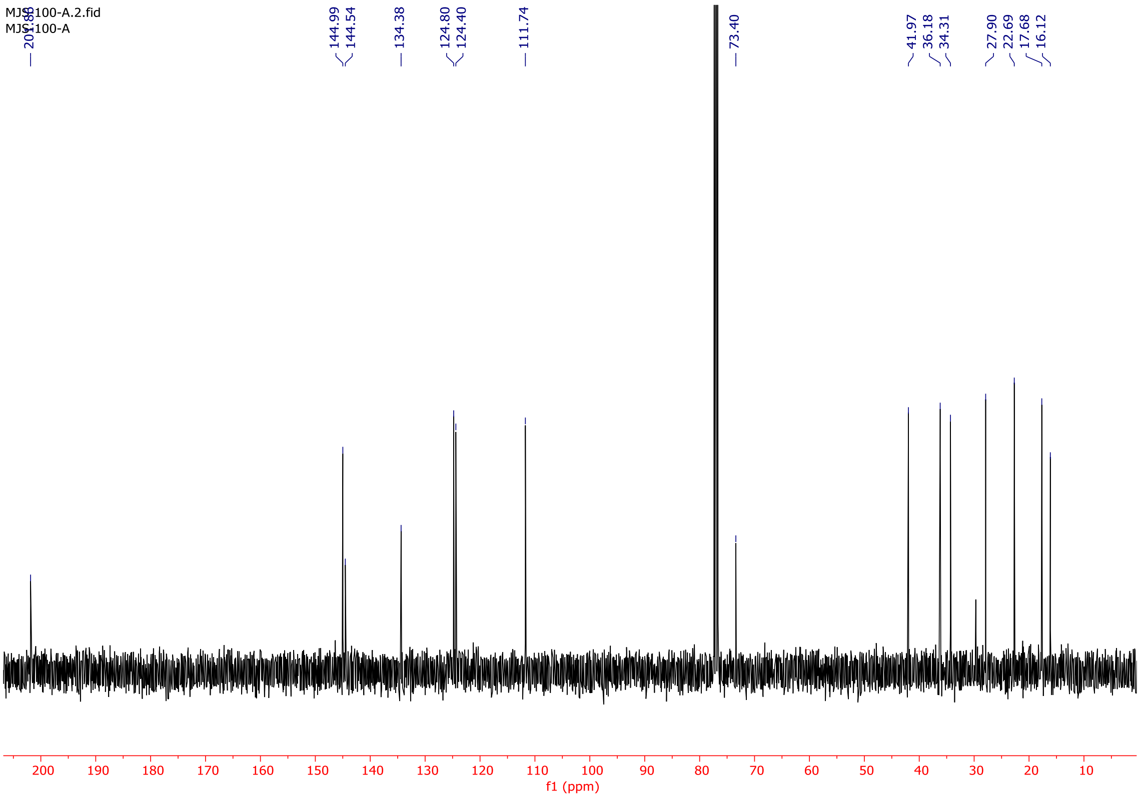

Figure S41. ^{13}C-NMR ($CDCl_3$, 100 MHz) spectrum of compound **28**

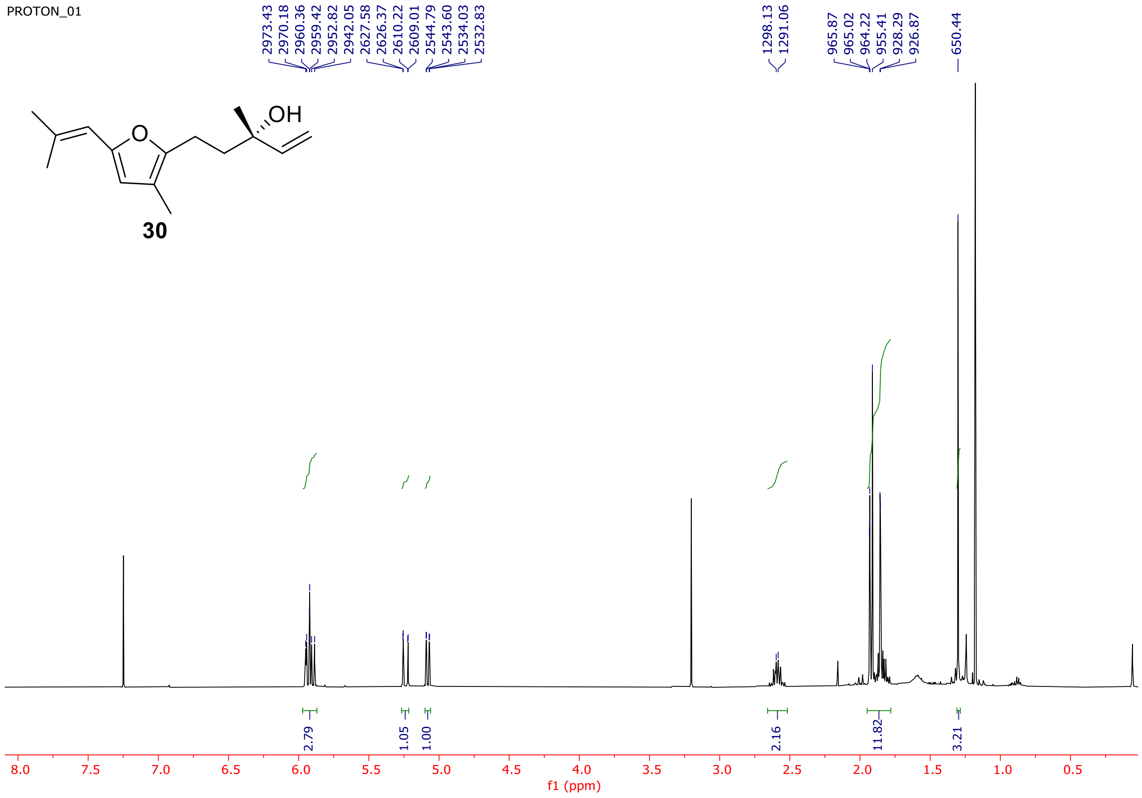

Figure S42. ^{1}H-NMR ($CDCl_3$, 400 MHz) spectrum of compound **30**

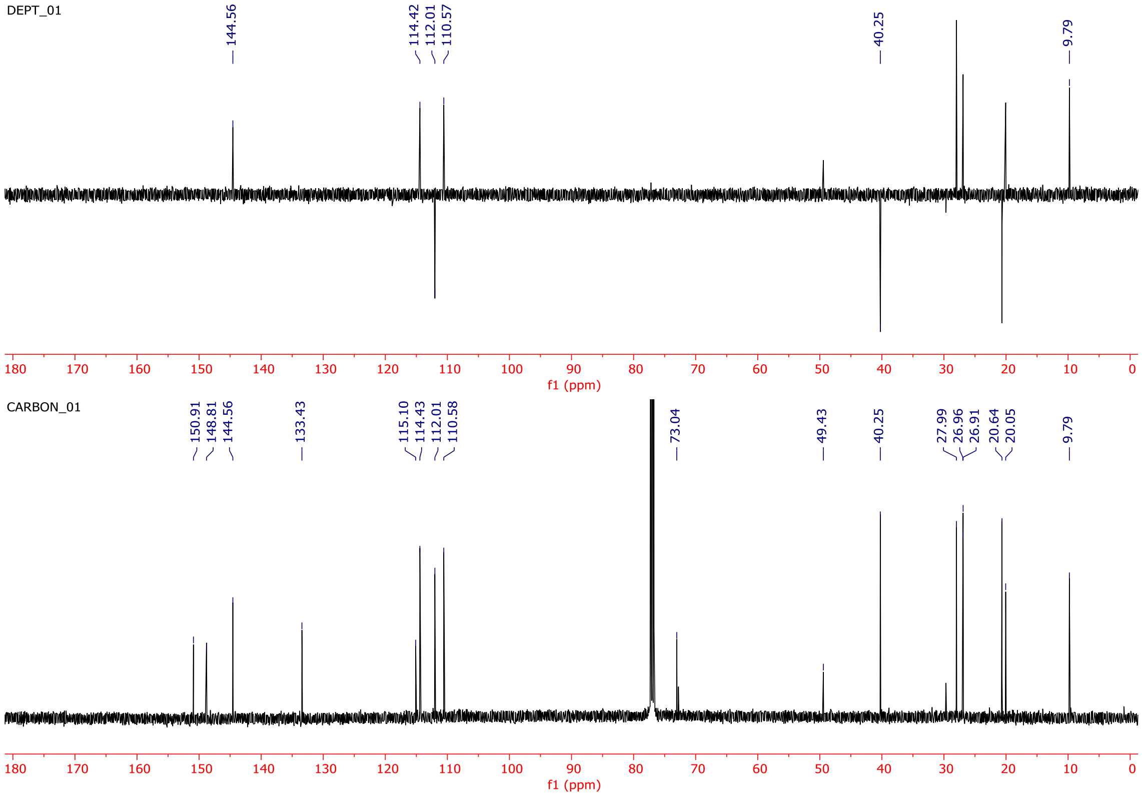

Figure S43. ^{13}C-NMR ($CDCl_3$, 100 MHz) spectrum of compound **30**

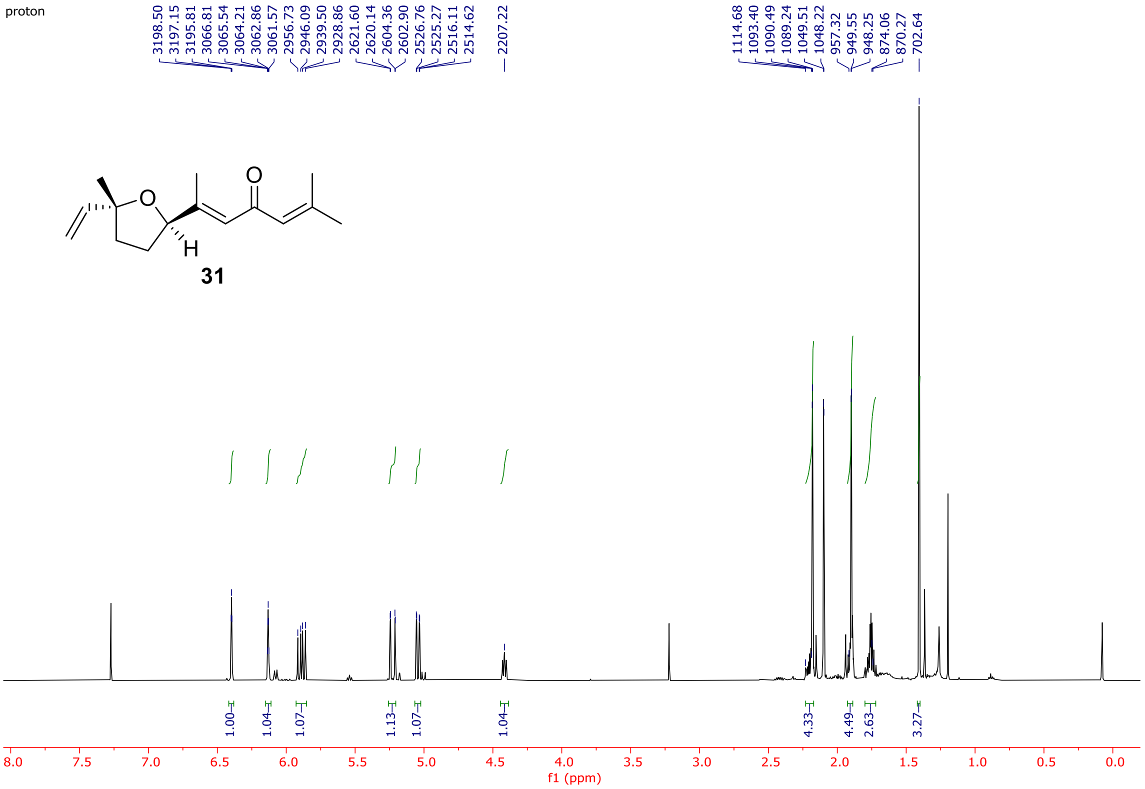

Figure S44. ^{1}H-NMR ($CDCl_3$, 400 MHz) spectrum of compound **31**

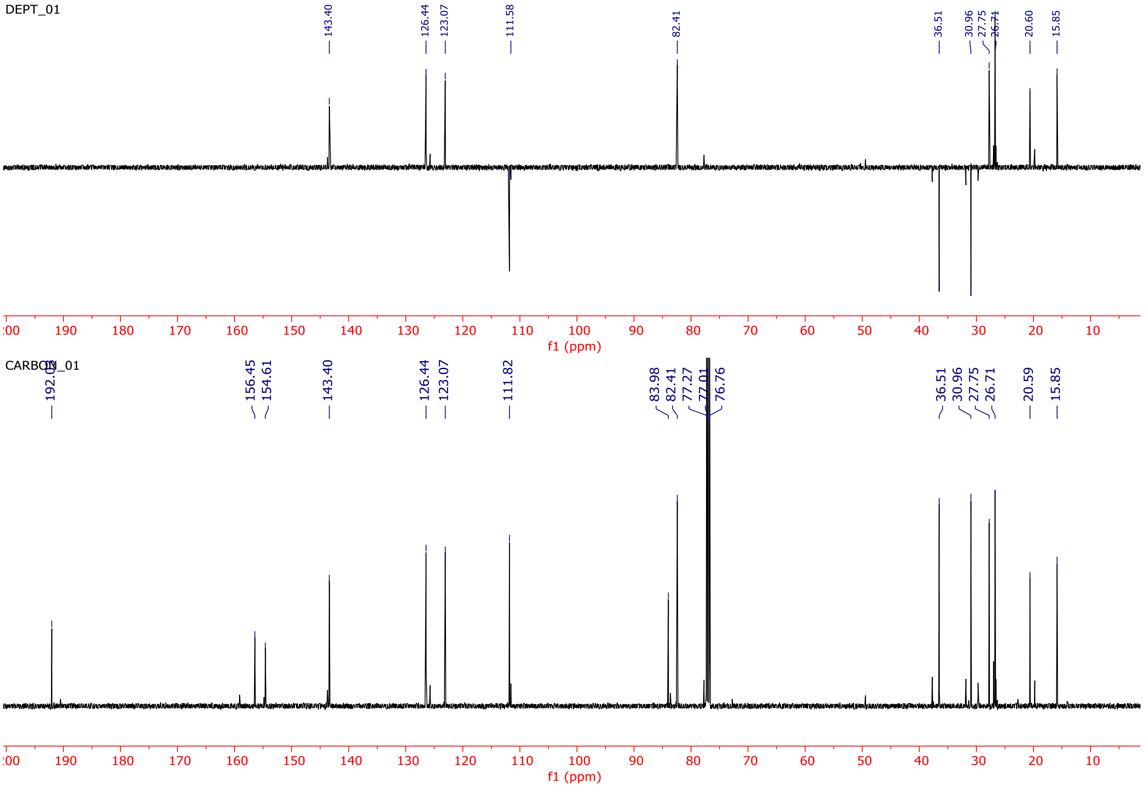

Figure S45. ^{13}C-NMR ($CDCl_3$, 100 MHz) spectrum of compound **31**

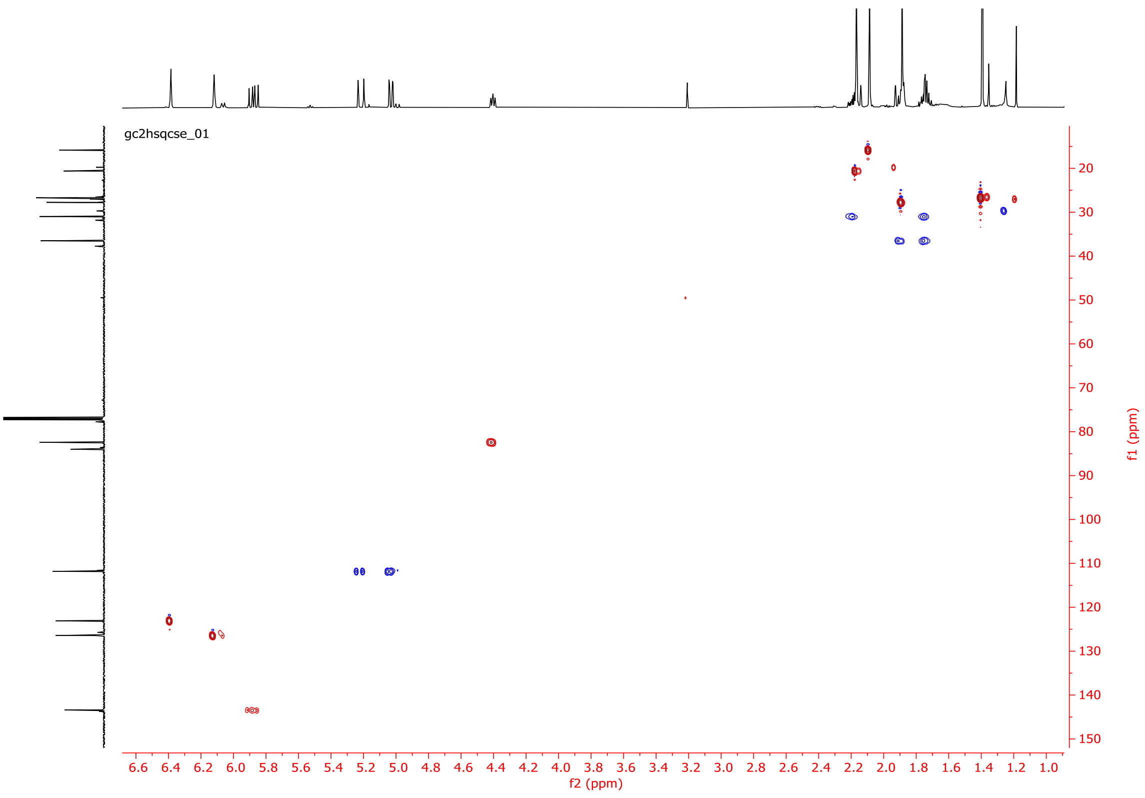

Figure S46. HSQC ($CDCl_3$, 400/100 MHz) of compound **31**

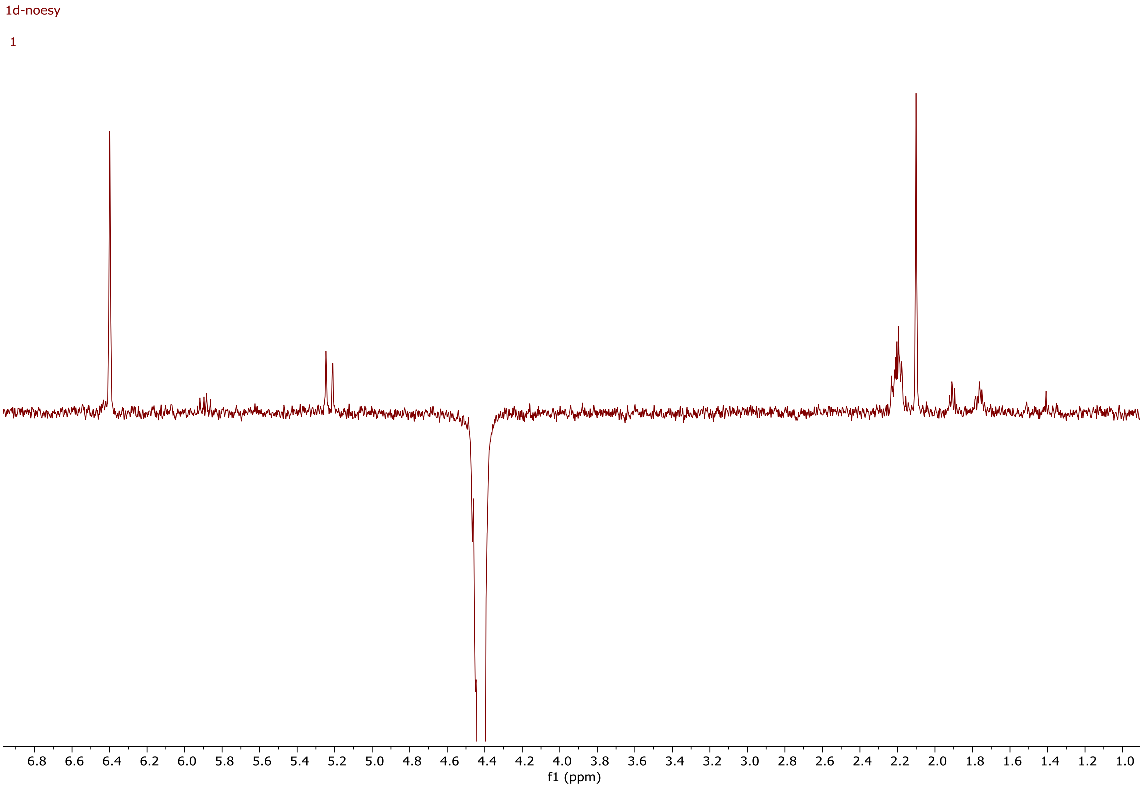

Figure S47. NOEDIFF ($CDCl_3$, 100 MHz) of compound **31**

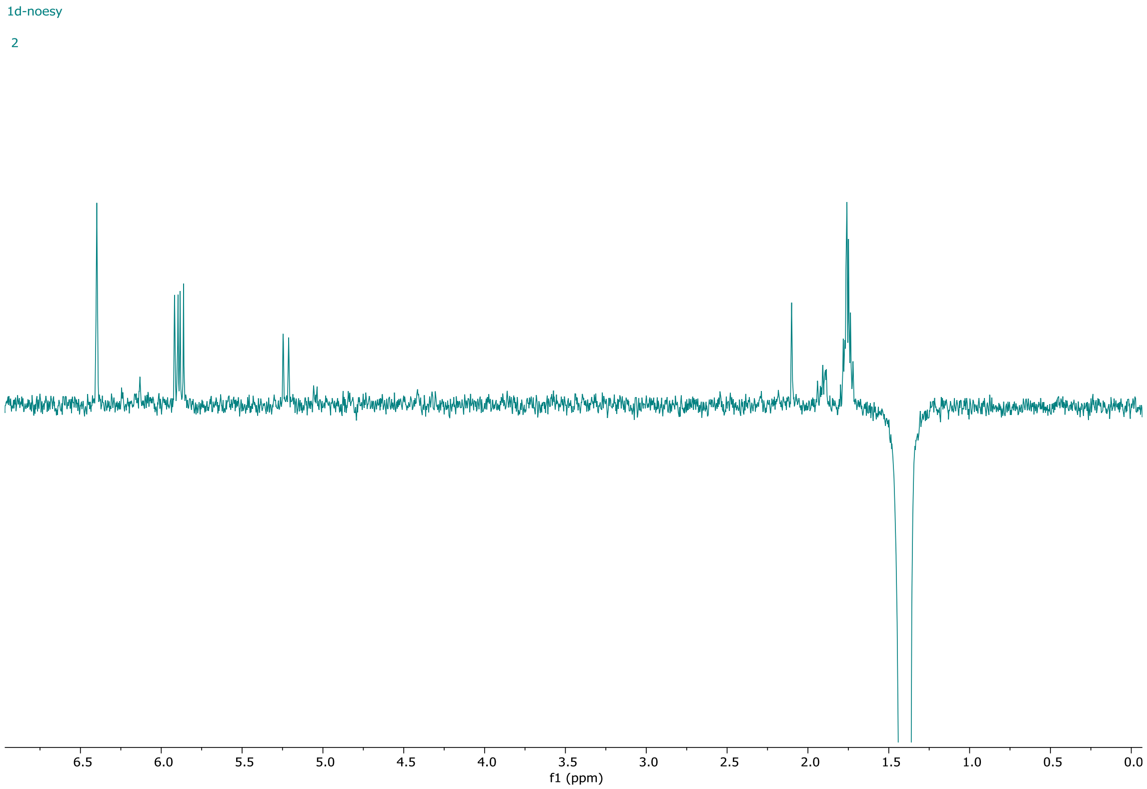

Figure S48. NOEDIFF ($CDCl_3$, 100 MHz) of compound **31**

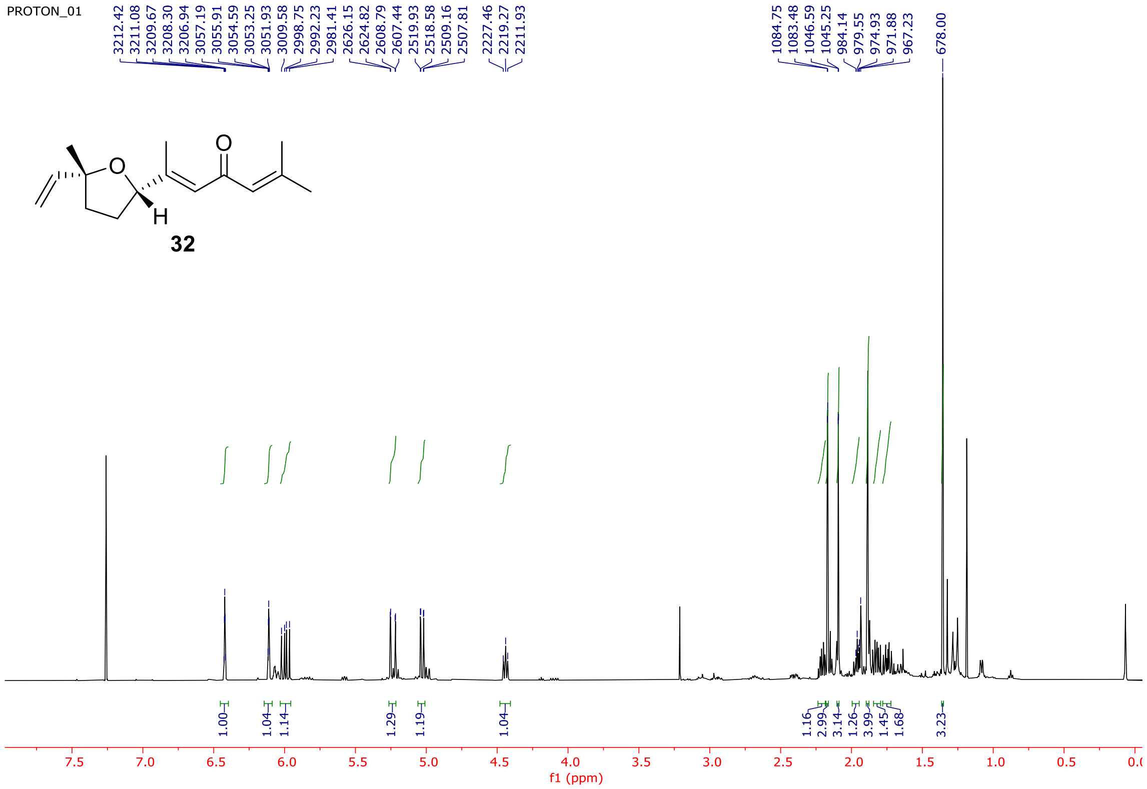

Figure S49. ^{1}H-NMR ($CDCl_3$, 500 MHz) spectrum of compound **32**

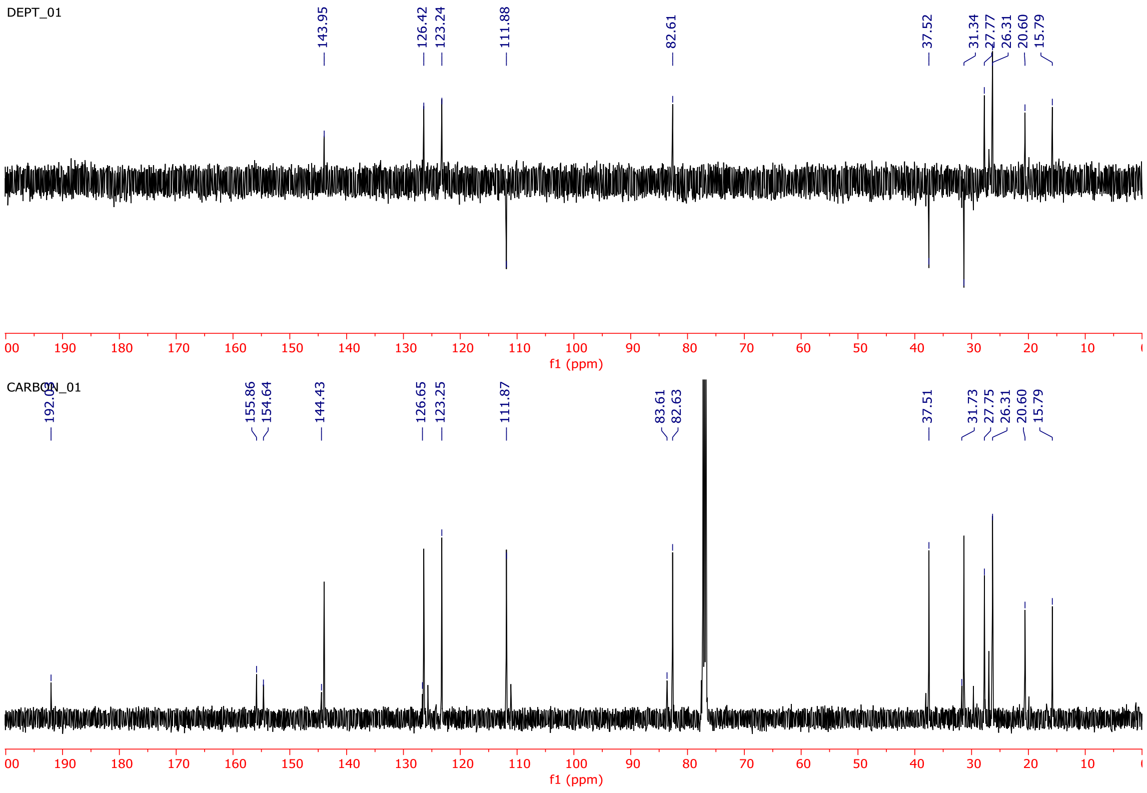

Figure S50. ^{13}C-NMR ($CDCl_3$, 125 MHz) spectrum of compound **32**

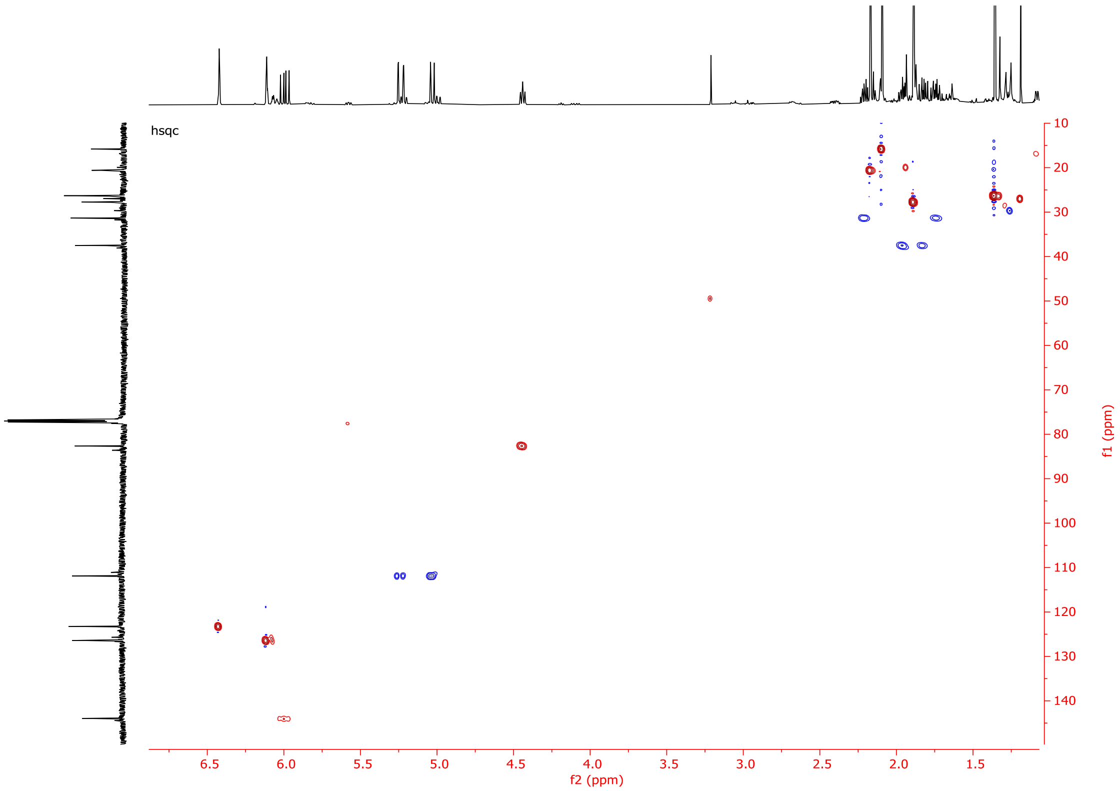

Figure S51. HSQC ($CDCl_3$, 500/125 MHz) of compound **32**

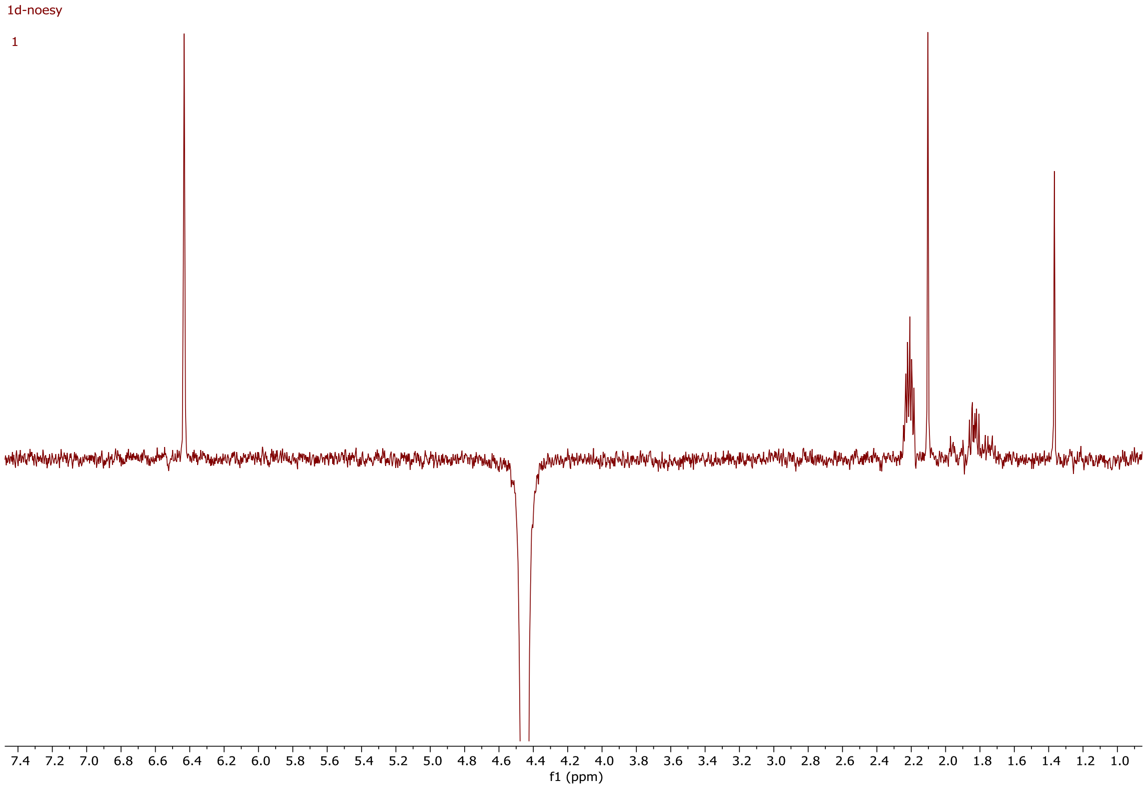

Figure S52. NOEDIFF ($CDCl_3$, 100 MHz) of compound **32**

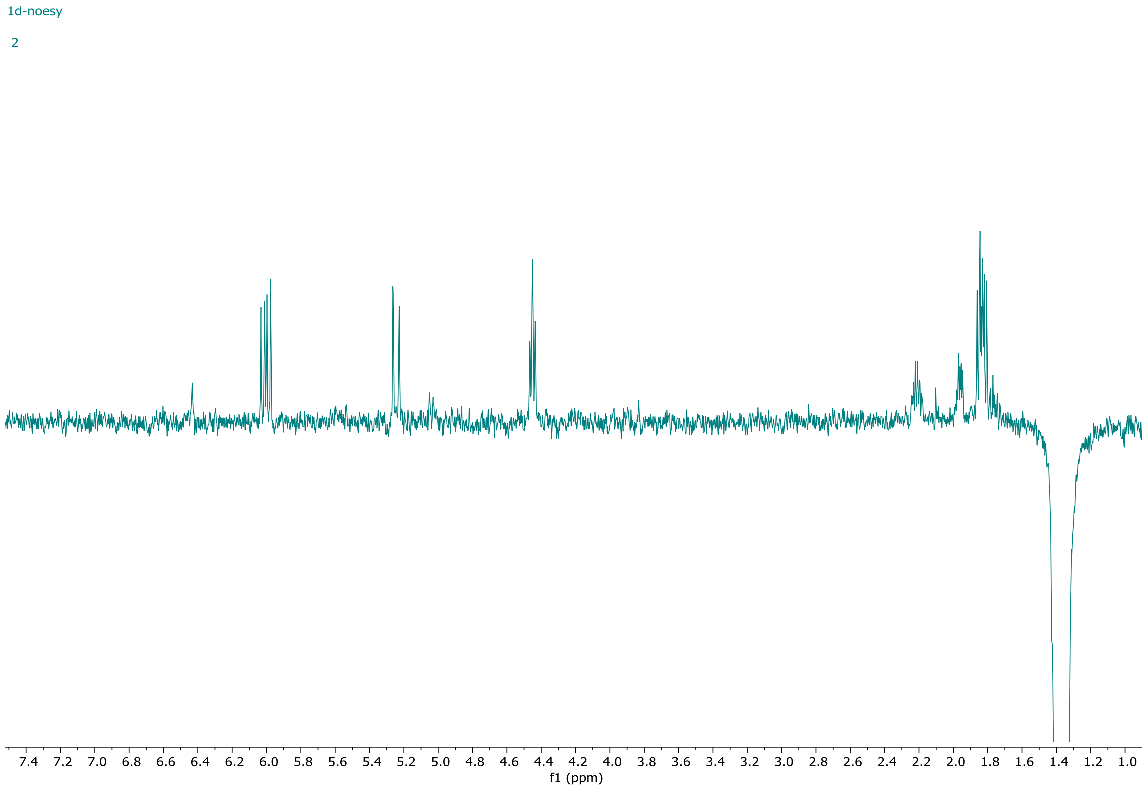

Figure S53. NOEDIFF ($CDCl_3$, 100 MHz) of compound **32**
